# Supplementary material for: Visual intracortical and transthalamic pathways carry distinct information to cortical areas
Source: Neuron. 2021 Jun 16;109(12):1996–2008.e6. doi: 10.1016/j.neuron.2021.04.017 (PMC8221812; doi:10.1016/j.neuron.2021.04.017)
Supplement: Document S1. Figures S1–S8 [file mmc1.pdf]

Neuron, Volume *109*

Supplemental information

Visual intracortical and transthalamic pathways carry distinct information to cortical areas

Antonin Blot, Morgane M. Roth, Ioana Gasler, Mitra Javadzadeh, Fabia Imhof, and Sonja B. Hofer

Visual intracortical and transthalamic pathways carry distinct information to cortical areas

Supplementary information

Acronyms

ACRONYM	FULL NAME	ACRONYM	FULL NAME
**a.u.**	Arbitrary units	**PM**	Posteromedial visual area
**AAV**	Adeno-associated virus	**POL**	Posterior limitans nucleus of the thalamus
**ACC**	Anterior cingulate cortex	**POR**	Postrhinal area
**AL**	Anterolateral visual area	**PPT**	Posterior pretectal area
**AM**	Anteromedial area	**PV**	Parvalbumin
**APN**	Anterior pretectal nucleus	**RHP**	Retrohippocampal region
**Apo**	Posterior auditory area	**RL**	Rostrolateral visual area
**AUD**	Auditory areas	**RN**	Red nucleus
**CTB**	CholeratoxinB	**RS**	Running speed
**CTX**	Cortex	**RSP**	Retrosplenial cortex
**DAPI**	4′,6-diamidino-2-phenylindole	**RSPagl**	Agranular retrosplenial cortex
**dLGN**	Dorsal lateral geniculate nucleus	**RSPd**	Dorsal part of the retrosplenial cortex
**ECT**	Ectorhinal areas	**RSPv**	Ventral part of the retrosplenial cortex.
**FWHM**	Full width half maximum	**RT**	Reticular thalamic nucleus.
**G**	Rabies virus glycoprotein	**SCdg**	Superior colliculus, deep gray layer
**GP**	Gaussian Process	**SCdw**	Superior colliculus, deep white layer
**HVA**	Higher visual areas	**SCig**	Superior colliculus, intermediate gray layer
**IGL**	Intrageniculate leaflet	**SCiw**	Superior colliculus, intermediate white layer
**INC**	Interstitial nucleus of Cajal	**SCop**	Superior colliculus, optic layer
**LD**	lateral dorsal nucleus of the thalamus	**SCsg**	Superior colliculus, superficial gray layer
**LI**	Laterointermediate area	**SCzo**	Superior colliculus, zonal layer
**LM**	Lateromedial area	**SF**	Spatial frequency
**LP**	Lateral posterior nucleus of the thalamus	**SS**	Somatosensory areas
**M1**	Primary motor cortex	**SubG**	Subgeniculate nucleus
**M2**	Secondary motor cortex	**TEa**	Temporal association area
**MPT**	Medial pretectal area	**TF**	Temporal frequency
**MRN**	Midbrain reticular nucleus	**TVA**	Tumor virus A protein
**ND**	Nucleus of Darkschewitsch	**V1**	Primary visual cortex
**NOT**	Nucleus of the optic tract	**VGluT2**	Vesicular glutamate transporter 2
**NPC**	Nucleus of the posterior commissure	**vLGN**	Ventral lateral geniculate nucleus
**OF**	Optic Flow	**WT**	Wild type
**OP**	Olivary pretectal nucleus	**ZI**	Zona incerta
**PL**	Prelimibic area		

Figure S1

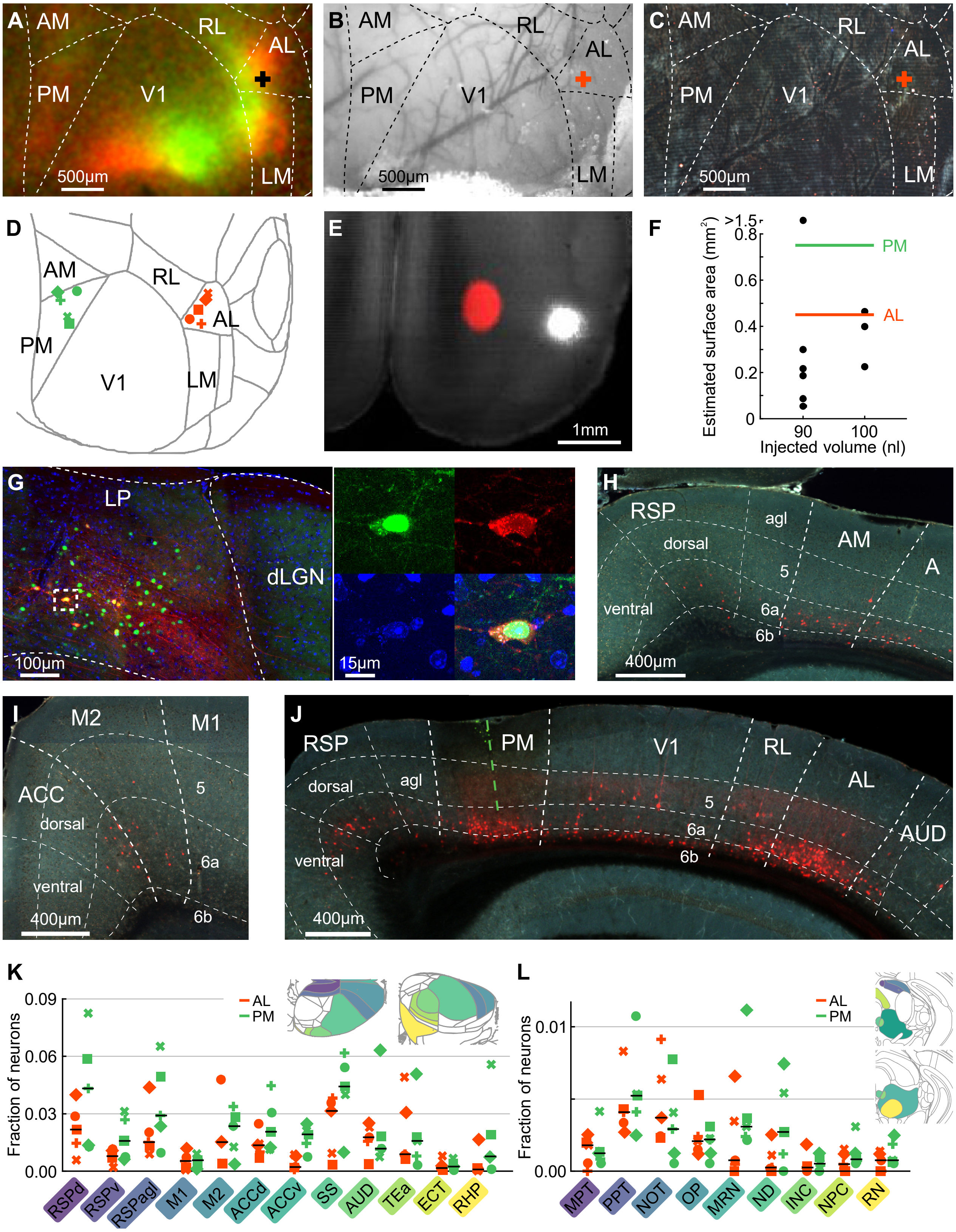

A
AM
RL
AL
PM
V1
LM
500µm
B
C
D
E
1mm
F
>1.5
Estimated surface area (mm²)
Injected volume (nl)
90
100
PM
AL
G
LP
dLGN
100µm
15µm
H
RSP
agl
dorsal
AM
A
ventral
5
6a
6b
400µm
I
M2
M1
ACC
dorsal
ventral
J
RSP
V1
RL
AL
AUD
K
Fraction of neurons
RSPd
RSPv
RSPagl
M1
M2
ACCd
ACCv
SS
AUD
TEa
ECT
RHP
L
MPT
PPT
NOT
OP
MRN
ND
INC
NPC
RN

Figure S1. Mono-synaptic rabies tracing: targeting of visual areas and additional data. Related to Figure 1.

(A) Example intrinsic signal imaging map used to identify and target higher visual area AL for injection of retroAAV-Cre. Intrinsic responses evoked by two spatially separated visual stimuli (see STAR Methods) are color-coded in green and red. The cross marks the injection site. AL: anterolateral area, AM: anteromedial area, LM: lateromedial area, PM: posteromedial area, RL: rostro lateral area, V1: primary visual cortex.

(B) Surface blood vessel pattern corresponding to the intrinsic imaging map in (A).

(C) Dorsal view of the same area shown in (A,B) in the perfused brain reconstructed after serial-section two-photon imaging. The brain was registered to the Allen common coordinate framework and area borders were aligned to the intrinsic map in (A) using the blood vessel pattern in (B).

(D) Dorsal view of cortical visual areas with retroAAV-Cre injection locations in AL and PM.

(E) Dorsal view of an example brain showing the extent of fluorescent protein expression after injections of two different AAV constructs (red: AAV-mCherry, injection of 100 nl; white: AAV-eBFP, injection of 90 nl). These experiments were performed to estimate the size of injections of retroAAV-Cre which could not be directly measured since the retroAAV construct was not coupled to a fluorescent protein.

(F) Surface area estimations of fluorescent protein expression after AAV injections of volumes similar to retroAAV-Cre injections. Surface areas are the full length at half maximum on the slice containing the core of the injection. Surface area sizes of AL and PM based on the Allen common coordinate framework are indicated by horizontal lines.

(G) Left: confocal image of a coronal slice through the lateral posterior thalamic nucleus (LP) injection site, showing starter neurons expressing G, TVA and rabies virus (green and red overlap), and neurons expressing G and TVA only (green). Dashed lines indicate the borders of LP and the dorsal lateral geniculate nucleus (dLGN). Right: magnified image of a rabies starter cell in LP. Nuclear green label indicates the presence of G protein, cytosolic green label reflects TVA expression and red label indicates the presence of rabies virus. DAPI staining is shown in blue.

(H,I) Example images of coronal slices showing rabies-labelled cells (red) presynaptic of AL-projecting LP neurons after retroAAV injection into AL. A: anterior visual area, ACC: anterior cingulate cortex, agl: agranular, AM: anteromedial area, M1: primary motor cortex, M2: secondary motor cortex, RSP: retrosplenial cortex.

(J) Example image of a coronal slice showing the cortical injection site of retroAAV-Cre in PM (pipette track marked in green) and rabies-expressing cells (red) presynaptic to PM-projecting LP neurons. AL: anterolateral area, agl: agranular, AUD: auditory areas, PM: postromedial area, RL: rostrolateral area, RSP: retrosplenial cortex, V1: primary visual cortex.

(K) Fraction of rabies-positive cells presynaptic to AL- (orange) and PM-projecting (green) LP neurons across cortical areas not detailed in Figure 1. ACCd: anterior cingulate cortex, dorsal part, ACCv: anterior cingulate cortex, ventral part, AUD: auditory areas, ECT: ectorhinal areas, M1: primary motor cortex, M2: secondary motor cortex, RHP: retrohippocampal region, RSPagl: agranular part of the retrosplenial cortex, RSPd: dorsal part of the retrosplenial cortex, RSPv: ventral part of the retrosplenial cortex, SS: somatosensory areas, TEa: temporal association areas.

(L) Fraction of rabies-positive presynaptic cells across midbrain areas, excluding the anterior pretectal nucleus and the superior colliculus (presented in Figure 1). INC: interstitial nucleus of Cajal, MPT: medial pretectal area, MRN: midbrain reticular nucleus, ND: nucleus of Darkschewitsch, NOT: nucleus of the optic tract, NPC: nucleus of the posterior commissure, OP: olivary pretectal nucleus, PPT: posterior pretectal area, RN; red nucleus.

Figure S2

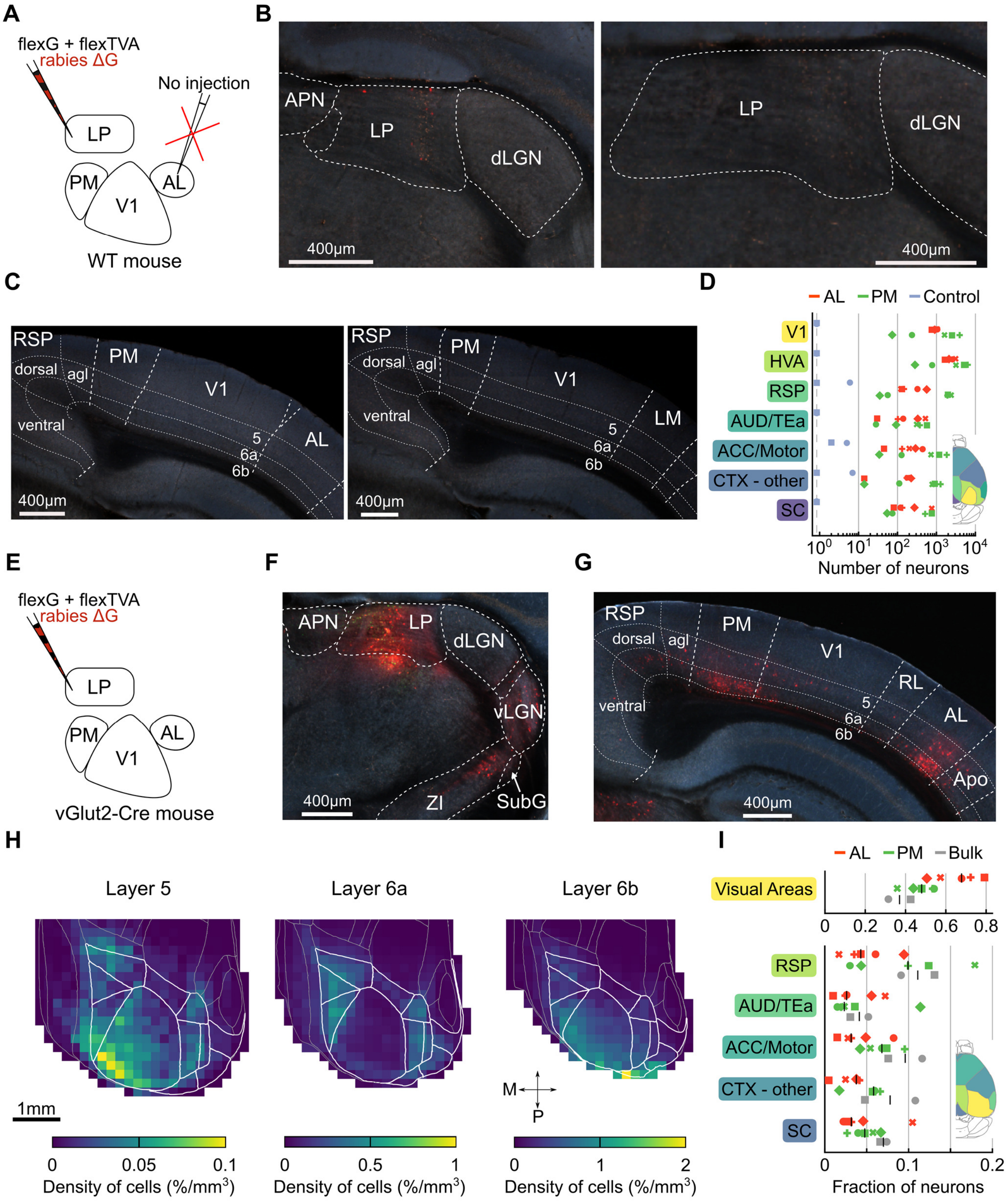
A
flexG + flexTVA
rabies ΔG
No injection
LP
PM
V1
AL
WT mouse
B
APN
LP
dLGN
400μm
C
RSP
dorsal
agl
ventral
PM
V1
AL
LM
5
6a
6b
400μm
D
AL
PM
Control
V1
HVA
RSP
AUD/TEa
ACC/Motor
CTX - other
SC
Number of neurons
E
flexG + flexTVA
rabies ΔG
vGlut2-Cre mouse
F
vLGN
ZI
SubG
G
RL
Apo
H
Layer 5
Layer 6a
Layer 6b
1mm
M
P
Density of cells (%/mm3)
I
Bulk
Visual Areas
Fraction of neurons

Figure S2. Mono-synaptic rabies tracing: control experiments and rabies tracing from LP neurons independent of projection target. Related to Figure 2.

(A) Design of rabies control experiment. The experimental protocol was similar to the one described in Figure 1, but no retroAAV-cre was injected. WT: wild-type

(B) Coronal sections through LP of two different animals showing no G or TVA expression.

(C) Coronal sections through the cortex from the same animals presented in (B) showing the absence of rabies-positive presynaptic cells.

(D) Numbers of rabies-positive cells detected by the automated cell counting software across brain areas in brains injected with retroAAV in AL (orange) in PM (green), and without injection of retroAAV-Cre (blue). Markers represent single animals. Dashed line indicates 0.

(E) Schematic of the experimental design. To label cells presynaptic to LP neurons without specific projection target, we injected AAV-flex-G, AAV-flex-TVA and rabies virus into LP of VGluT2-Cre mice.

(F) Coronal slice through the LP injection site showing rabies-positive cells in subcortical areas, presynaptic to LP neurons without specific projection target.

(G) Coronal slice showing rabies-positive cells presynaptic to LP neurons in cortical areas.

(H) Dorsal view of the average relative density of cells presynaptic to LP neurons per volume (see STAR Methods) in layer 5 (left), layer 6a (middle) and layer 6b (right); 2 mice. White lines indicate the border of cortical areas as in Figure 2A.

(I) Fraction of rabies-positive cells presynaptic to AL-projecting LP neurons (orange), PM-projecting LP neurons (green), and the general LP population (grey). Markers represent single animals. Bottom right corner: dorsal view of color-coded cortical areas.

ACC/Motor: anterior cingulate areas and motor areas, agl: agranular, AL: anterolateral area, APN: anterior pretectal nucleus, Apo: posterior auditory area, AUD/TEa: auditory areas and temporal association areas, CTX: cortex, dLGN: dorsal lateral geniculate nucleus, HVA: higher visual areas, LM: lateromedial area, LP: lateral posterior nucleus, PM: posteromedial area, RL: rostrolateral area, RSP: retrosplenial cortex, SC: superior colliculus, SubG: subgeniculate nucleus, V1: primary visual area, vLGN: ventral lateral geniculate nucleus, WT: wild type. ZI: zona incerta

Figure S3

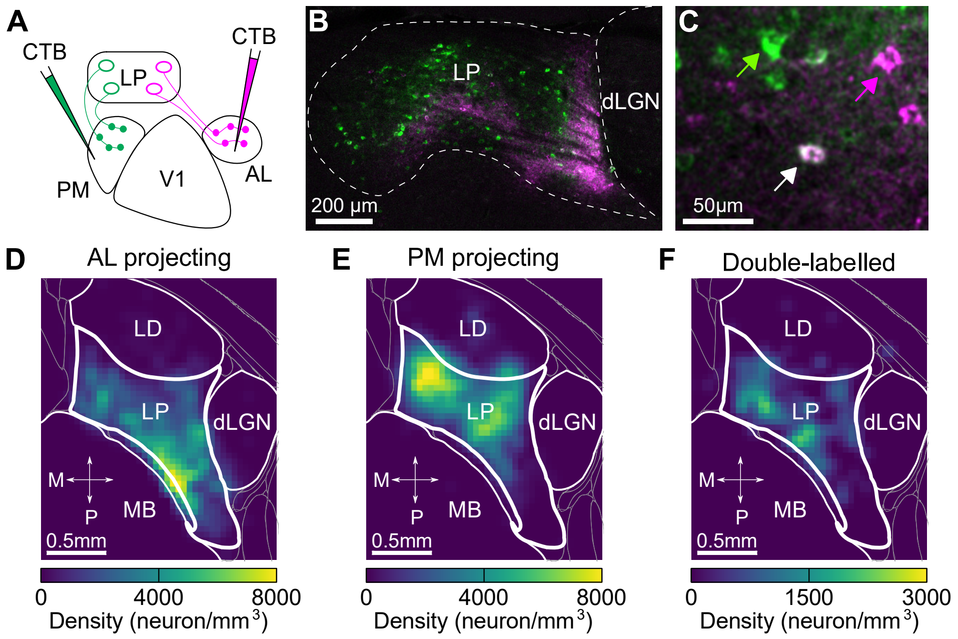

Figure S3. LP neurons projecting to AL and PM form two largely distinct populations. Related to Figures 1 and 2.

(A) Schematic of the experimental design. To determine the extent of overlap between AL and PM-projecting LP neurons, retrograde tracers of different colors (cholera toxin subunit B, CTB) were injected into AL and PM.

(B) Coronal section through LP showing the spatial distribution of PM-projecting (green) and AL-projecting (pink) neurons.

(C) Magnified area from (B). Arrows indicate examples of a PM-projecting LP neuron (green), an AL-projecting neuron (pink), and a neuron that targets both PM and AL (double-labelled, white).

(D-F) Dorsal view of the average relative density of AL-projecting (**D**, 3504 and 2102 neurons), PM-projecting (**E**, 2123 and 2406 neurons), and double-labelled neurons in LP (**F**, 442 and 414 neurons). Data from two mice. AL: anterolateral area, CTB: choleratoxin subunit B, dLGN: dorsal lateral geniculate nucleus, LD: lateral dorsal nucleus, LP: lateral posterior nucleus, M: medial, P: posterior, V1: primary visual cortex.

Figure S4

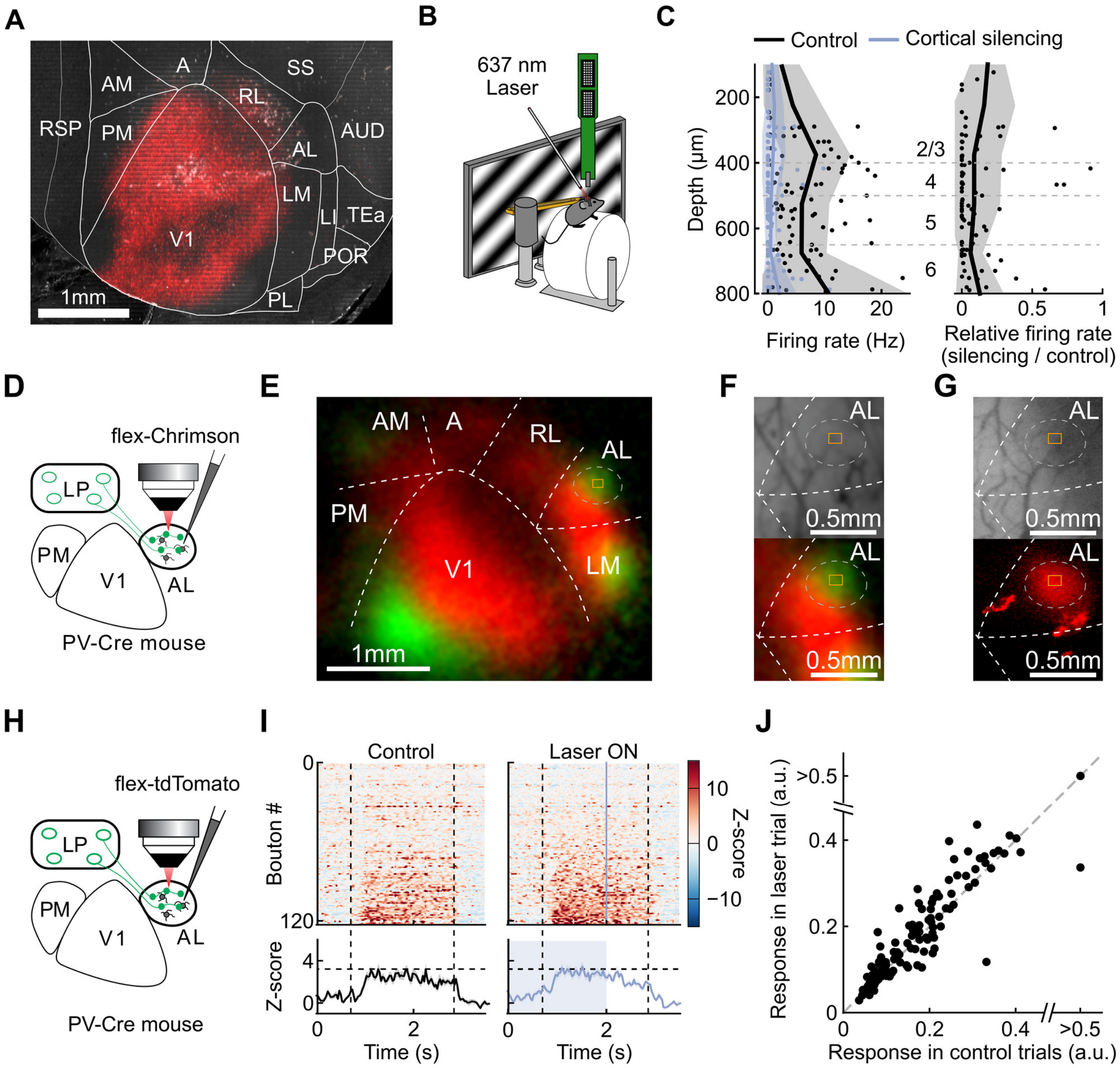

Figure S4. Optogenetic activation of PV neurons silences all cortical layers while laser light without Chrimson expression has no effect on LP responses. Related to Figure 3.

(A) Dorsal view of the cortex reconstructed after serial-section two-photon imaging showing the extent of Chrimson expression (red) in V1. The brain was registered to the Allen common coordinate framework (limits of cortical areas are indicated by white lines).

(B) Schematic of the experimental design for electrophysiological recordings. The cranial window was removed and a multi-channel silicon probe was inserted into a cortical location with Chrimson expression in AL, PM or V1. Awake mice were presented with gratings and a 637-nm laser was used to activate Chrimson-expressing PV neurons to silence cortical activity similar to experiments in Figure 3.

(C) Left: mean firing rate during visual stimulation with (blue) and without (black) laser; Right: proportion of remaining response in laser trials as a function of recording depth for single units not excited by the laser recorded from a silicon probe in a cortical location with Chrimson expression

during optogenetic activation of PV neurons. Dots depict individual units. Shading indicates standard deviation. 42 single units, 10 mice.

(D) Schematic of the experiment: flex-Chrimson was injected in AL of PV-Cre mice. GCaMP6f was expressed in LP and the activity of LP boutons was recorded in AL.

(E) Example intrinsic imaging map obtained to determine the location of cortical area AL for two-photon imaging and injection of AAV-flex-Chrimson showing responses to two spatially separated visual stimuli (red and green). Yellow square indicates the imaging site.

(F) Bottom: zoomed-in cutout from image in (E). Top: corresponding surface blood vessel pattern.

(G) Top: surface blood vessel pattern used to find the imaging site corresponding to (F). Bottom: image showing the extent of Chrimson expression (red) in AL, approximated by the dotted circle. Red specks outside the circle are imaging artifacts. Yellow square indicates the imaging site.

(H) Schematic of the laser control experiment. PV-Cre mice were injected with AAV-flex-tdTomato in AL and GCaMP6f in LP. Visually-evoked activity of LP boutons in AL was imaged with and without laser stimulation over AL.

(I) Top: time course of z-scored neuronal activity of individual boutons. For each bouton, activity was averaged across grating stimuli evoking a response (see STAR Methods) in control trials (left) and laser trials (right). Responses are aligned to the onset of the laser. Blue shading indicates time of laser stimulation. Dashed lines show duration of moving grating. Bottom: Average response across all boutons. Grey shading indicates sem. 123 boutons from 4 sessions in 4 mice

(J) Relationship between the average response of individual boutons with and without laser stimulation.

Figure S5

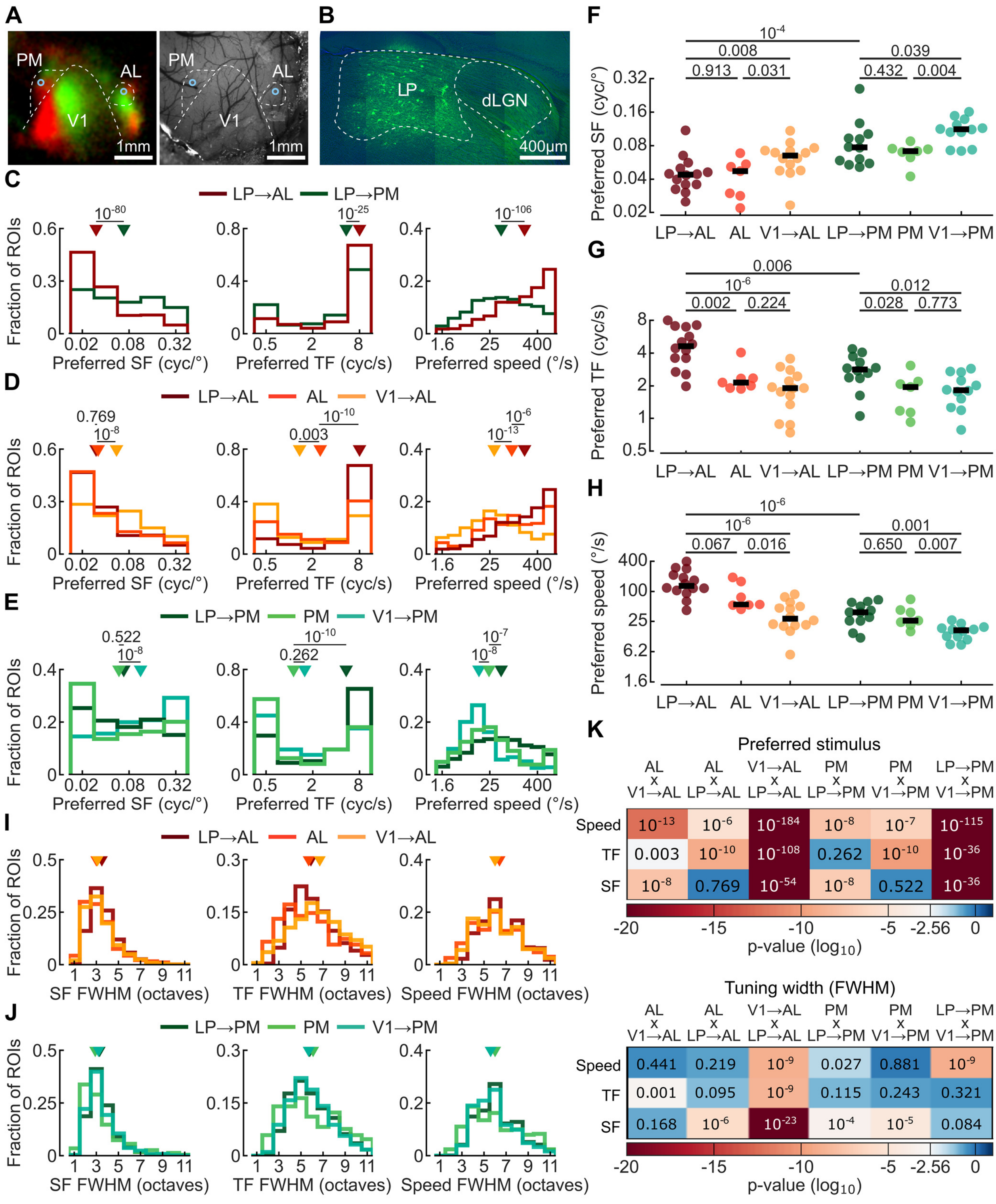
A
B
C
D
E
F
G
H
I
J
K
PM
AL
V1
1mm
LP
dLGN
400μm
LP→AL
LP→PM
AL
V1→AL
PM
V1→PM
Fraction of ROIs
Preferred SF (cyc/°)
Preferred TF (cyc/s)
Preferred speed (°/s)
SF FWHM (octaves)
TF FWHM (octaves)
Speed FWHM (octaves)
Preferred stimulus
Tuning width (FWHM)
Speed
TF
SF
p-value (log10)

Figure S5. Further characterization of visual responses of LP inputs, V1 inputs and local neurons recorded in areas AL and PM. Related to Figure 4.

(A) Example intrinsic imaging map (left) showing responses to two spatially separated visual stimuli in green and red, and image of blood vessel pattern of the imaging area under the cranial window (right). Blue circles indicate the two imaging sites recorded in the posteromedial area (PM) and the anterolateral area (AL).

(B) Example image of GCamP6f injection site. dLGN: dorsal lateral geniculate nucleus, LP: lateral posterior nucleus.

(C) Distribution of preferred spatial frequency (left, 2237 LP boutons in AL and 2059 LP boutons in PM), preferred temporal frequency (middle, 1333 and 1128 LP boutons for AL and PM) and preferred speed (right, 2468 and 2342 boutons for AL and PM respectively) of grating stimuli for significantly modulated LP boutons recorded in AL (dark red) and in PM (green). Triangles indicate the median.

(D) Same as (C) for AL neurons (orange, 172, 126, and 231 neurons for SF, TF and speed respectively), LP boutons (dark red) and V1 boutons (yellow, 2555, 1637, and 2928 boutons for SF, TF and speed respectively) recorded in AL.

(E) Same as (C) for PM neurons (green, 214, 181, and 288 neurons for SF, TF and speed respectively), LP boutons (dark green) and V1 boutons (blue, 2327, 1659, and 2535 boutons for SF, TF and speed respectively) recorded in PM.

(F) Median preferred spatial frequencies of grating stimuli per imaging session of LP boutons in AL and PM, AL and PM neurons, and V1 boutons in AL and PM. Circles represent individual sessions. Black dashes indicate the median of all sessions.

(G) Same as (F) for preferred temporal frequencies of grating stimuli.

(H) Same as (F) for preferred grating speed.

(I) Distribution of full width half maxima (FWHM) of GP fit prediction (see STAR Methods) of spatial frequency (SF) tuning curves at the preferred temporal frequency and preferred direction of the grating (left), of temporal frequency (TF) tuning curves at the preferred spatial frequency and preferred direction (middle), and of speed tuning curves at the preferred grating direction (right).

(J) Same as (I) for full width half maxima of tuning curves of LP boutons in PM, PM neurons and V1 boutons in PM.

(K) P-values of Wilcoxon rank-sum test for relevant pairwise comparisons of stimulus preference (top) and tuning width (bottom). Numbers show raw p-values, color code indicates significance level, adjusted for multiple comparison using Bonferroni correction: white is the threshold, shades of red indicate significant p-values, shades of blue non-significant p-values. In all panels data from 7 sessions in 5 mice for AL neurons, 8 sessions in 5 mice for PM neurons, 14 sessions in 14 mice for LP boutons in AL, 12 sessions in 10 mice for LP boutons in PM, 14 sessions in 7 mice for V1 boutons in AL and 12 sessions in 7 mice for V1 boutons in PM.

Figure S6

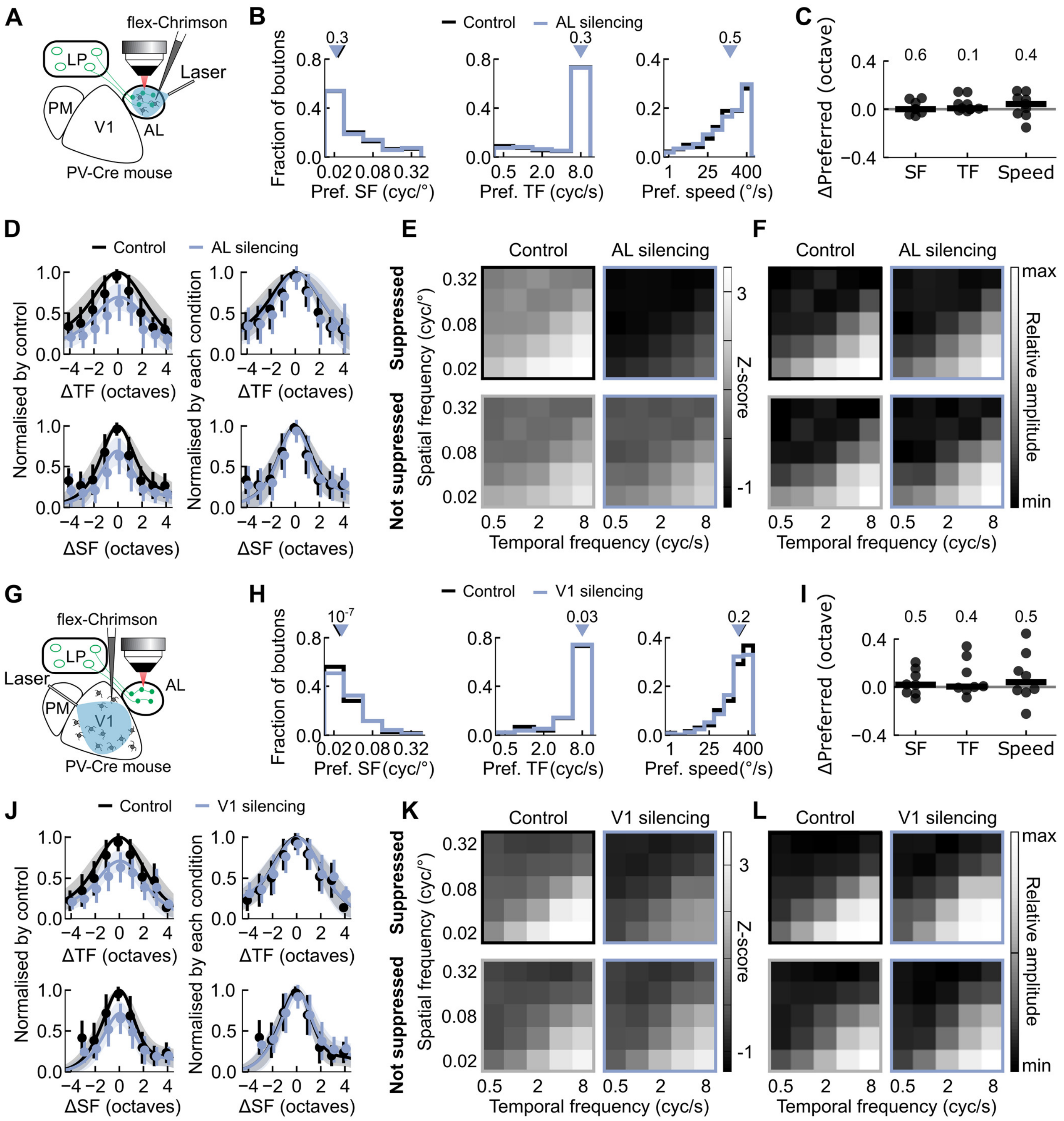

Figure S6. Cortical silencing of AL or V1 has minor effects on the response preferences and selectivity of LP inputs to AL. Related to Figures 3 and 4.

(A) Schematic of the experimental design, as in Figure 3F. Two-photon imaging of LP boutons was performed in AL while PV cells expressing Chrimson in cortical area AL were optogenetically activated to suppress local cortical activity.

(B) Distribution of preferred spatial frequency (left, 631 boutons), preferred temporal frequency (middle, 348 boutons) and preferred speed (right, 758 boutons) for boutons suppressed by AL silencing in control trials and AL silencing trials. Triangles indicate medians. P-values are indicated above. In panels (B-F) data from 9 sessions in 6 mice.

(C) Changes of stimulus preferences (laser trials – control trials) averaged by session. Black dots indicate single sessions, horizontal bar shows the median, p-values are indicated above.

(D) Left: Mean response curves to varying temporal (top) and spatial (bottom) grating frequencies of boutons significantly, but not fully suppressed during AL silencing (see STAR Methods, 181 and 269 boutons for TF and SF) in control trials (black) and laser trials (blue), normalized by the response to the preferred stimulus in control trials, plotted centered on and relative to the preferred frequency. Right: same as on the left, but normalized by the maximum response within each condition. Dots and error bars indicate means and the standard deviation of the raw data across boutons, while the lines and shading indicate means and standard deviation obtained from the prediction of the GP fit (see STAR Methods).

(E) Average spatial and temporal frequency population response matrices as in Figure 4 for LP boutons that were suppressed (top, 1009 boutons) or not suppressed (bottom, 1278 boutons) by AL silencing, in control trials (left) and silencing trials (right). Pearson correlation coefficient between the average response matrix of suppressed boutons in control trials and response matrices of individual sessions: 0.9 +/- 0.2 and 0.8 +/- 0.1 for suppressed and not suppressed boutons respectively, p = 0.3.

(F) Average population response matrices with values normalized to the maximum response amplitude during control trials (left) and AL silencing trials (right) for boutons that were suppressed (top) or not suppressed (bottom) by AL silencing.

(G) Schematic of the experimental design, as in Figure 3A. Two-photon imaging of LP boutons was performed in AL while PV cells expressing Chrimson in cortical area V1 were optogenetically activated to suppress local cortical activity.

(H) Distribution of preferred spatial frequency (left, 386 boutons), preferred temporal frequency (middle, 265 boutons) and preferred speed (right, 421 boutons) for boutons suppressed by V1 silencing in control trials and V1 silencing trials. Triangles indicate medians. In panels H-L data from 8 sessions in 3 mice.

(I) Same as (C) for experiments with V1 silencing.

(J) Same as (D) for experiments with V1 silencing.136 and 176 boutons for TF and SF respectively

(K) Average spatial and temporal frequency population response matrices of LP boutons in AL that were suppressed (top, 477 boutons) or not suppressed (bottom, 455 boutons) by V1 silencing, in control trials (left) and silencing trials (right). Pearson correlation coefficient between the average response matrix of suppressed boutons in control trials and response matrices of individual sessions: 0.9 +/- 0.2 and 0.9 +/- 0.1 for suppressed and not suppressed boutons respectively, p = 0.9.

(L) Average population response matrices for LP boutons in AL with values normalized to the maximum response amplitude during control trials (left) and V1 silencing trials (right) for boutons that were suppressed (top) or not suppressed (bottom) by V1 silencing.

Figure S7

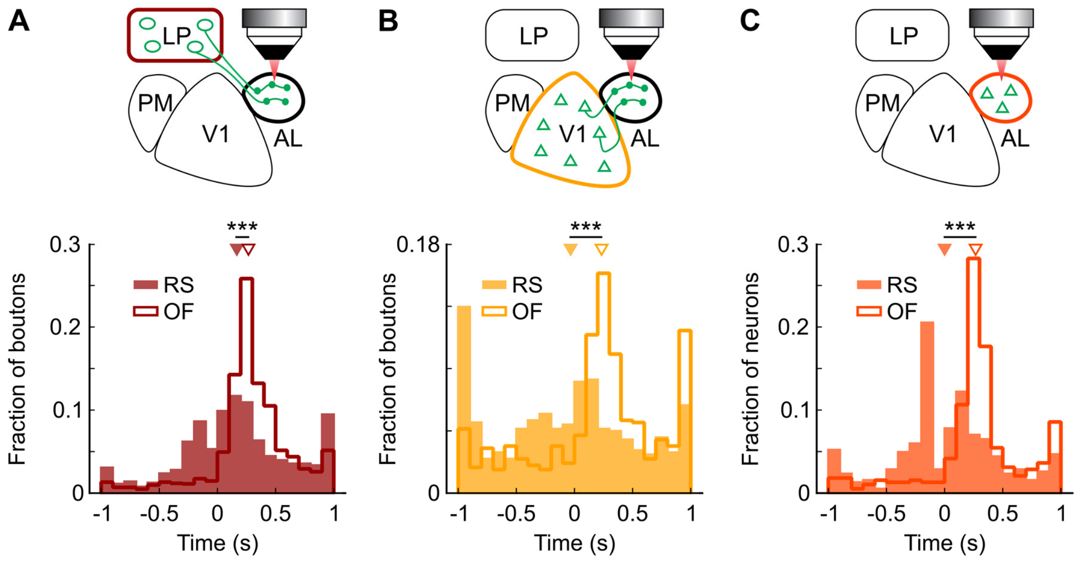

Figure S7. Distribution of lag times with highest absolute correlation coefficients between activity and running speed or optic flow speed. Related to STAR Methods.

(A-C) Top: schematic of the experimental design. Bottom: distribution of lag times between running speed (RS, filled histogram) or optic flow (OF, line) and the activity of LP boutons imaged in AL (A, 1437 boutons with correlation coefficients above the circular threshold of 0.1 from 43 sessions in 18 mice), V1 boutons in AL (B, 647 boutons with a correlation above the circular threshold of 0.1 from 6 sessions in 3 mice) and AL neurons (C, 385 neurons with correlation coefficients above the circular threshold of 0.1 from 15 sessions in 5 mice) for which the absolute cross-correlation was maximal for each neuron.***: $p < 10^{-15}$.

Figure S8

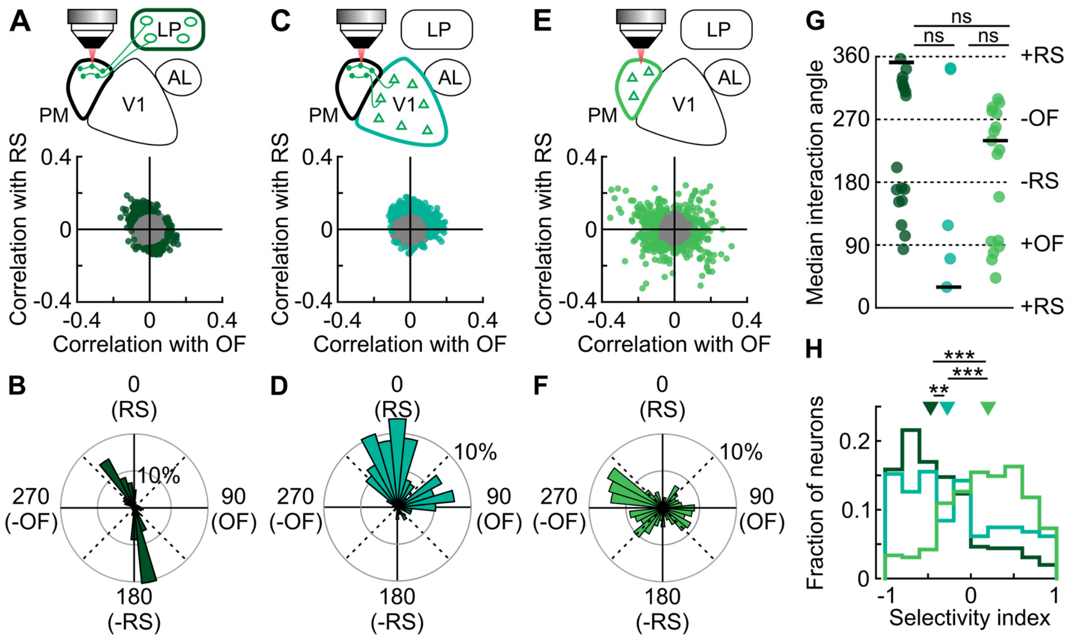

Figure S8. Thalamic and cortical inputs convey distinct visuo-motor information to area PM. Related to Figure 5.

(A) Top: schematic of the recording configuration. GCaMP6f was expressed in LP while calcium activity of LP boutons in cortical area PM was recorded using two-photon imaging. Bottom: relationship between the mean cross-correlation coefficients (see STAR Methods) of neuronal activity with running speed and optic flow speed for all responsive LP boutons (5678 boutons from 19 sessions in 8 mice). Only boutons with mean cross-correlation greater than 0.1 (colored points in scatter plot) were included in the analysis shown in (B), (G) and (H).

(B) Histogram in polar coordinates showing the distribution of interaction angles between the mean cross-correlation of activity with running speed (RS) and optic flow speed (OF) for LP boutons imaged in PM (425 boutons from 19 sessions in 8 mice).

(C) Same as (A) for V1 boutons imaged in PM (1556 boutons from 5 sessions in 3 mice).

(D) Same as (B) for V1 boutons imaged in PM (309 boutons from 5 sessions in 3 mice).

(E) Same as (A) for cortical neurons imaged in PM (976 neurons from 17 sessions in 5 mice).

(F) Same as (B) for cortical neurons imaged in PM (356 neurons from 17 sessions in 5 mice)

(G) Median interaction angles across single sessions for LP boutons in PM (left), V1 boutons in PM (middle) and PM neurons (right). Black horizontal lines represent the circular median across sessions. ns: non-significant, LP vs V1 $p = 0.011$, LP vs PM $p = 0.73$, V1 vs PM $p = 0.19$

(H) Distribution of selectivity indices (difference of the absolute mean cross-correlation of neuronal activity with optic flow and running speed divided by their sum) for individual LP boutons in PM (dark green), PM neurons (green) and V1 boutons in PM (blue). -1 indicates a high correlation only with running speed, 1 a high correlation only with optic flow and 0 indicates equally high correlation with running speed and optic flow of individual boutons/neurons. **: $p < 10^{-4}$; ***: $p < 10^{-16}$.
