# Supplementary material for: Visual intracortical and transthalamic pathways carry distinct information to cortical areas
Source: Neuron. 2021 Jun 16;109(12):1996–2008.e6. doi: 10.1016/j.neuron.2021.04.017 (PMC8221812; doi:10.1016/j.neuron.2021.04.017)
Supplement: Document S2. Article plus supplemental information [file mmc2.pdf]

Neuron

Article

Visual intracortical and transthalamic pathways carry distinct information to cortical areas

Highlights

- Transthalamic pathway through pulvinar indirectly connects lower to higher cortical areas
- This pathway combines input from V1 with that of many cortical and subcortical areas
- Pulvinar conveys distinct visual and motor information to different higher visual areas
- Direct intracortical and transthalamic pathways convey different information

Authors

Antonin Blot, Morgane M. Roth, Ioana Gasler, Mitra Javadzadeh, Fabia Imhof, Sonja B. Hofer

Correspondence

s.hofer@ucl.ac.uk

In brief

Sensory perception relies on information flow between cortical areas that are connected not only via direct intracortical pathways but also indirect transthalamic pathways. Here, Blot et al. report that these transthalamic pathways transmit specific visual and contextual information to visual cortical areas, different from the signals conveyed by intracortical feedforward pathways.

Blot et al., 2021, Neuron *109*, 1996–2008
June 16, 2021 © 2021 The Author(s). Published by Elsevier Inc.
https://doi.org/10.1016/j.neuron.2021.04.017

CellPress

CellPress
OPEN ACCESS

Neuron

Article

Visual intracortical and transthalamic pathways carry distinct information to cortical areas

Antonin Blot,[1,2,3] Morgane M. Roth,[2,3,4] Ioana Gasler,[1,2] Mitra Javadzadeh,[1,2] Fabia Imhof,[2] and Sonja B. Hofer[1,2,5,*]
[1]Sainsbury Wellcome Centre for Neural Circuits and Behaviour, University College London, London, UK
[2]Biozentrum, University of Basel, Basel, Switzerland
[3]These authors contributed equally
[4]Present address: Institute of Molecular and Clinical Ophthalmology Basel, 4031 Basel, Switzerland
[5]Lead contact
*Correspondence: s.hofer@ucl.ac.uk
https://doi.org/10.1016/j.neuron.2021.04.017

SUMMARY

Sensory processing involves information flow between neocortical areas, assumed to rely on direct intracortical projections. However, cortical areas may also communicate indirectly via higher-order nuclei in the thalamus, such as the pulvinar or lateral posterior nucleus (LP) in the visual system of rodents. The fine-scale organization and function of these cortico-thalamo-cortical pathways remains unclear. We find that responses of mouse LP neurons projecting to higher visual areas likely derive from feedforward input from primary visual cortex (V1) combined with information from many cortical and subcortical areas, including superior colliculus. Signals from LP projections to different higher visual areas are tuned to specific features of visual stimuli and their locomotor context, distinct from the signals carried by direct intracortical projections from V1. Thus, visual transthalamic pathways are functionally specific to their cortical target, different from feedforward cortical pathways, and combine information from multiple brain regions, linking sensory signals with behavioral context.

INTRODUCTION

Our perception of the environment is thought to rely on neuronal interactions within the cerebral cortex, where sensory information is processed by hierarchical pathways involving many cortical areas (Van Essen, 1979). However, all cortical areas are also highly interconnected with the thalamus, from which the cortex receives the majority of its input. First-order thalamic nuclei convey information from the sense organs to primary sensory areas in the neocortex and have been extensively characterized (Guillery and Sherman, 2002; Jones, 1985). However, the larger part of the sensory thalamus consists of so-called higher-order nuclei, which form extensive and intricate circuits with cortical areas (Guillery and Sherman, 2002; Jones, 1985; Sherman, 2016).

The higher-order thalamic nucleus of the visual system is the pulvinar complex, also known as the lateral posterior nucleus (LP) in rodents (Baldwin et al., 2017; Bennett et al., 2019; Roth et al., 2016; Zhou et al., 2017). Pulvinar projections to primary visual cortex (V1) target mostly cortical layers 1 and 5a and have been shown to convey contextual information (Roth et al., 2016) that can sharpen visual representations (Fang et al., 2020; Hu et al., 2019). However, the pulvinar provides more pronounced input to higher visual areas, where it also targets the cortical input layer 4 and can strongly impact cortical activity (Beltramo and Scanziani, 2019; Soares et al., 2004; Zhou et al., 2016).

The pulvinar receives most of its input from visual brain areas. While some of its subdivisions are innervated by the superior colliculus, the main input to large parts of the pulvinar comes from visual areas in the neocortex (Baldwin et al., 2017; Beltramo and Scanziani, 2019; Bennett et al., 2019; Roth et al., 2016; Rovó et al., 2012; Shipp, 2003; Zhou et al., 2017). Therefore, this higher-order thalamic complex has been proposed to form transthalamic pathways, whereby layer 5 cortical cells of a lower-order area drive thalamocortical cells that project to a higher-order cortical area (Sherman, 2016; Sherman and Guillery, 2011). These indirect feedforward pathways via the thalamus would parallel direct intracortical feedforward connections, for instance from V1 to a higher visual area. While anatomical projection patterns are compatible with this hypothesis (Bennett et al., 2019; Shipp, 2003), the fine-scale input and output connectivity of pulvinar neurons has not been determined. It is therefore still unresolved if they are part of transthalamic feedforward pathways between cortical areas. Alternatively, pulvinar circuits could provide additional visual pathways from the retina to the cortex via the superior colliculus or form specific, reciprocal loops with individual cortical areas (Beltramo and Scanziani, 2019; Bennett et al., 2019; Guo et al., 2017, 2020; Wurtz et al., 2011). Furthermore, it is unclear what information these pathways through the pulvinar bring to cortical visual areas and how the signals they convey differ from those carried by direct intracortical projections.

 © 2021 The Author(s). Published by Elsevier Inc.
This is an open access article under the CC BY license (http://creativecommons.org/licenses/by/4.0/).

CellPress
OPEN ACCESS

To address these questions, we focused on higher-order thalamic circuits of the mouse visual system. More than a dozen higher visual areas have been described in the mouse neocortex (Wang and Burkhalter, 2007; Zhuang et al., 2017), including the anterolateral (AL) area and the posteromedial (PM) area. The visual response properties of AL and PM are different from V1 and distinct from each other. The function of these visual areas is still unclear, but AL may be specialized to process visual motion, as neurons in AL preferentially respond to moving stimuli of low spatial and high temporal frequency, while PM neurons on average prefer high spatial and low temporal frequency stimuli (Andermann et al., 2011; Marshel et al., 2011; Roth et al., 2012; de Vries et al., 2020). Both areas receive prominent input from V1 and the mouse homolog of the pulvinar, LP (Bennett et al., 2019; Glickfeld et al., 2013; Roth et al., 2016; Wang and Burkhalter, 2007).

Using monosynaptic rabies tracing, we found that the population of LP neurons projecting to either of these cortical areas combines information from V1 layer 5 cells with signals from many other cortical and subcortical areas, including superior colliculus. Optogenetic silencing of different cortical areas confirmed that LP neurons projecting to higher visual areas are strongly influenced by V1 activity but also receive significant input from the area they are projecting to. Two-photon calcium imaging of axonal boutons revealed that LP sends specific signals to higher visual areas that differ from those carried by the direct cortical feedforward pathway from V1. In behaving animals, direct projections from V1 to cortical area AL carry information about visual motion in the environment, while LP input to AL combines information about visual motion and the animals' own movement. In summary, our results indicate that LP is a key node of feedforward transthalamic pathways that convey information distinct from V1 intracortical feedforward projections and may link sensory signals with the behavioral context in which they are encountered.

RESULTS

LP neurons projecting to higher visual areas receive inputs from many cortical and subcortical areas

LP is interconnected with all visual areas. However, the sources of inputs to LP neurons projecting to a specific higher visual area are unknown. LP could potentially form strong reciprocal loops with the cortex, whereby thalamic neurons receive most of their input from their cortical target area (Guo et al., 2017, 2020). Alternatively, LP could be part of transthalamic pathways whereby thalamic neurons projecting to a higher visual area receive their inputs from other structures, such as V1 or the superior colliculus (Beltramo and Scanziani, 2019; Sherman, 2016; Sherman and Guillery, 2011). To address this question, we performed projection-specific monosynaptic rabies tracing, specifically labeling cells presynaptic of AL-projecting LP neurons (Figures 1, S1, and S2A–S2D; Reardon et al., 2016; Schwarz et al., 2015; Wall et al., 2010; Wickersham et al., 2007; see STAR Methods).

We found that LP neurons projecting to AL received synaptic input from a large number of brain areas (Figures 1B–1H, S1H, S1I, S1K, and S1L), resembling the general pattern of inputs to LP (Figures S2E–S2I; Bennett et al., 2019; Roth et al., 2016). Cells providing input to AL-projecting LP neurons were particularly abundant in visual cortical areas (Figures 1B–1D, 2A, and 2B). Presynaptic neurons were also located in the ipsilateral superior colliculus (Figures 1E and 1F) as well as in cortical association areas, in particular the retrosplenial cortex and anterior cingulate and secondary motor cortices (Figure 1D, S1H, S1I, and S1K). Notably, LP neurons received input from several areas containing mainly inhibitory neurons, including the thalamic reticular nucleus, the zona incerta, the ventral lateral geniculate nucleus, and the anterior pretectal nucleus (Figures 1G and 1H, Halassa and Acsády, 2016; Sabbagh et al., 2020), revealing LP as a target of multiple long-range inhibitory circuits.

To determine if this brain-wide pattern of input connectivity is specific to AL-projecting LP neurons or a general feature of LP thalamocortical pathways, we investigated the connectivity of LP neurons projecting to a different visual cortical area, PM. First, to test if LP projections to AL and PM originate from distinct populations of neurons, we injected differently colored retrograde tracers into the two cortical areas (Figure S3). In agreement with a previous study (Juavinett et al., 2020), we found that only a small subset of LP neurons was double labeled (7.8%; Figure S3F), indicating that the projections from LP to AL and PM are largely distinct. We then determined the sources of presynaptic input to PM-projecting LP neurons, employing projection-specific monosynaptic rabies tracing as described above (Figure S1J). PM-projecting LP neurons had a distribution of presynaptic inputs that was largely similar to that of AL-projecting neurons (Figures 1D, 1F, 1H, S1K, and S1L), suggesting that the pattern of inputs to LP thalamocortical pathways generalize across higher visual target areas. Notably, PM- and AL-projecting neurons had comparable fractions of presynaptic cells in AL and PM (Figure 2A and 2B), suggesting that LP neurons do not preferentially receive reciprocal input from their cortical target area. Slight differences in the distribution of inputs to PM- and AL-projecting neurons were, however, apparent; PM-projecting neurons tended to be innervated to a larger extent by nonvisual cortical areas (Figures 1D and S1K) and deeper layers of SC (Figure 1F).

Distribution of cortical input to LP shows the hallmarks of feedforward transthalamic pathways

Cortical efferents have been described to differentially affect their target neurons, depending on the cortical layer they originate from. The main driving input onto thalamic neurons from the cortex is thought to arise from layer 5 cells, while layer 6 cells are assumed to provide weaker or modulatory feedback (Crick and Koch, 1998; Jones, 1985; Rockland, 1996; Sherman, 2016; Sherman and Guillery, 2011). To determine the cortical origin of putative driving and modulatory inputs onto AL- and PM-projecting LP neurons, we quantified the number of presynaptic cells in each layer of visual cortical areas (Figures 2C–2E). We found that presynaptic layer 5 cells were not predominantly located in the cortical target area of either AL- or PM- projecting LP neurons but were by far most numerous in V1. In contrast, the density of presynaptic layer 6a cells was much higher in higher visual areas than in V1. Presynaptic layer 6b cells showed a distribution similar to layer 5 inputs to LP and may therefore represent a cell class distinct from layer 6a (Hoerder-Suabedissen

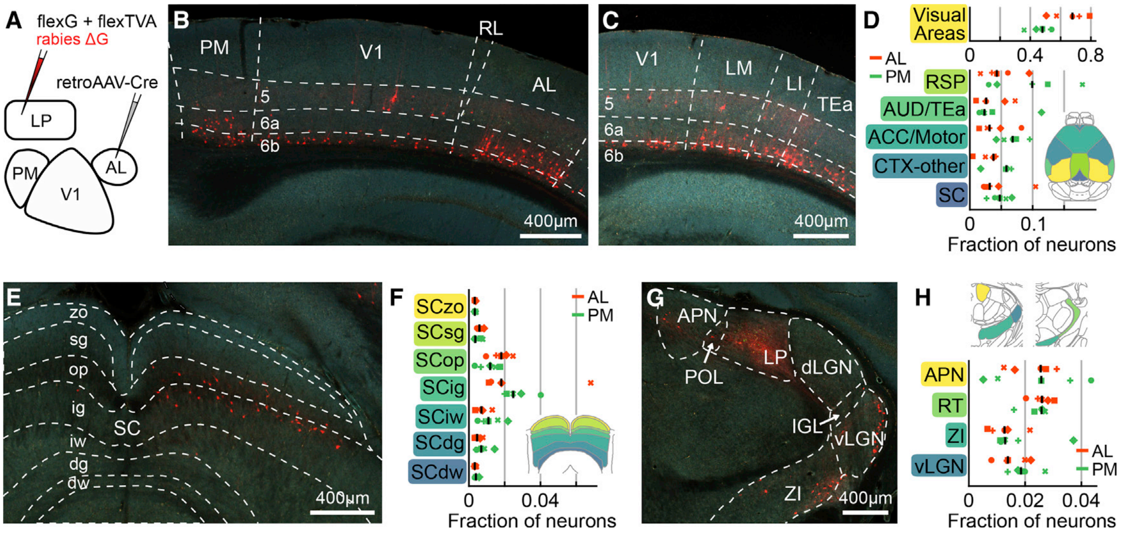

Figure 1. LP neurons projecting to higher visual areas receive input from many cortical and subcortical regions

(A) Schematic of the experimental design to specifically label cells presynaptic to AL- or PM-projecting LP neurons.

(B and C) Example images showing rabies-labeled neurons (red) presynaptic of AL-projecting LP neurons in visual areas. Numbers indicate the cortical layers. AL, anterolateral area; LI, latero-intermediate area; LM, lateromedial area; PM, posteromedial area; RL, rostrolateral area; TEa, temporal association areas; V1, primary visual cortex.

(D) Relative distribution of cells presynaptic to AL-projecting LP neurons (orange, 5 mice) and PM-projecting LP neurons (green, 5 mice) as a fraction of total cells per brain. Symbols denote individual brains (similar across all plots). Here, and in all figures, black lines indicate median values. Inset: dorsal view of color-coded mouse brain. ACC/Motor, anterior cingulate cortex and motor areas; AUD/TEa, auditory and temporal association areas; CTX-other, remaining cortical areas; SC, superior colliculus; RSP, retrosplenial cortex.

(E) Example image of cells presynaptic to AL-projecting LP neurons in the superior colliculus. SC, superior colliculus; zo, zonal layer; sg, superficial gray layer; op, optic layer; ig, intermediate gray layer; iw, intermediate white layer; dg, deep gray layer; dw, deep white layer.

(F) Distribution of cells presynaptic to AL-projecting (orange) and PM-projecting LP neurons (green) across layers of the superior colliculus as a fraction of total cells per brain. Inset: coronal view of color-coded superior colliculus layers.

(G) Example image of cells presynaptic to AL-projecting LP neurons in inhibitory prethalamic and pretectal structures. APN, Anterior pretectal nucleus; dLGN, dorsal lateral geniculate nucleus; IGL, intrageniculate leaflet; POL, posterior limitans nucleus of the thalamus; vLGN, ventral lateral geniculate nucleus; ZI, zona incerta.

(H) Distribution of presynaptic cells across inhibitory structures in the prethalamus and pretectum as a fraction of total cells per brain. Top shows coronal view of color-coded areas. RT, reticular thalamic nucleus.

See also Figures S1 and S3.

et al., 2018). Together, these results suggest that LP pathways appear to have a strong feedforward component, whereby LP neurons integrate driving inputs from V1 layer 5 cells with information from many other cortical and subcortical areas.

Functional influence of cortical inputs on LP pathways

Monosynaptic retrograde rabies tracing can provide an indication of anatomical connectivity, but this method does not determine the functional influence of presynaptic inputs on target neurons. To test how different cortical areas affect activity in LP projection pathways, we optogenetically suppressed activity in these areas *in vivo* by activating the depolarizing opsin Chrimson (Klapoetke et al., 2014), delivered via adeno-associated virus (AAV) injection, in parvalbumin-positive interneurons (Figures 3 and S4A–S4C). Previous studies have shown that silencing V1 strongly suppresses activity in the frontal subregions of LP (Beltramo and Scanziani, 2019; Bennett et al., 2019). To confirm that this holds true for LP neurons projecting to higher visual area AL, we suppressed activity in V1 while simultaneously imaging visual responses of LP boutons in AL (Figures 3A–3E). Silencing V1 strongly decreased visually evoked activity in a large fraction of LP boutons in AL (61.8% ± 30.6% of suppressed boutons per session and 32.9% ± 23.0% decrease in average response amplitude per session [median ± interquartile range]), confirming that V1 activity has a strong influence on LP neurons projecting to higher visual area AL.

To test the functional importance of reciprocal loops in visual thalamocortical circuits, we next silenced AL while imaging LP boutons in AL (Figures 3F–3J and S4D–S4G). Optogenetic silencing of AL also had a surprisingly strong suppressive effect on LP bouton activity (39.4% ± 28.6% of suppressed boutons in each session and 21.5% ± 22.4% decrease in average response amplitude per session). This decrease in activity was not observed in control animals without opsin expression (Figures S4H–S4J). Therefore, AL provides significant input onto LP neurons projecting back to this cortical area. We repeated the same experiment for LP-PM thalamocortical circuits and silenced cortical visual area PM while imaging activity of LP boutons in PM (Figures 3K–3O). Silencing PM had a smaller but significant effect on visually evoked activity (8.7% ± 11.6% decrease in average response amplitude per session). However, LP boutons that were significantly affected (14.4% ± 17.8% of boutons per

CellPress
OPEN ACCESS

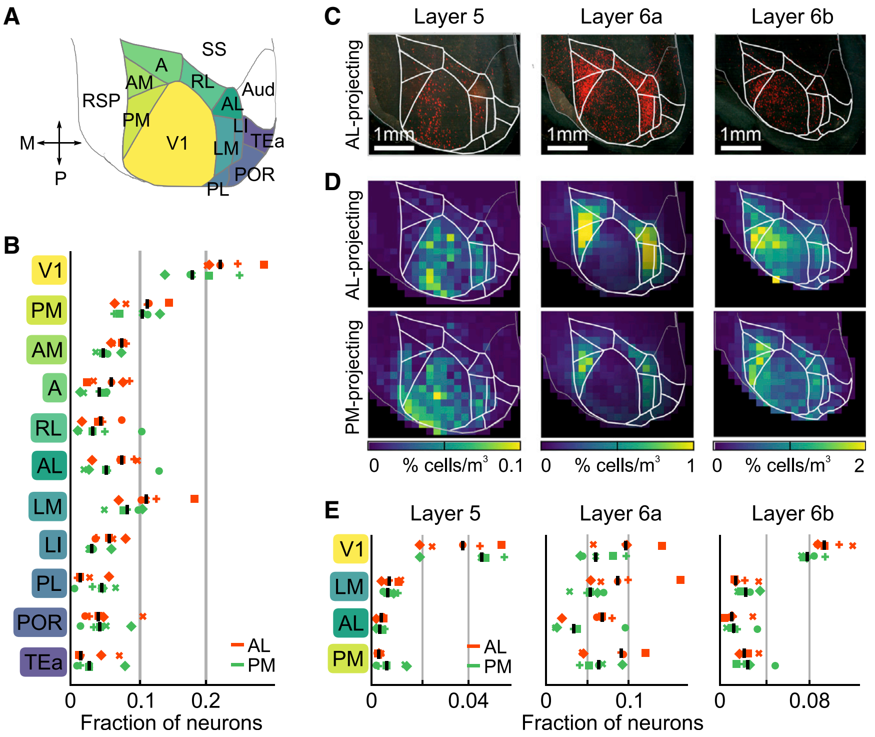

Figure 2. Distribution of cortical input to LP shows the hallmarks of feedforward transthalamic pathways

(A) Schematic dorsal view of color-coded cortical visual areas as shown in (C) and (D). A, anterior area; AL, anterior-lateral area; AM, anterior medial area; Aud, auditory areas; LI, laterointermediate area; LM, lateromedial area; PL, prelimbic area; PM, posteromedial area; POR, postrhinal area; RL, rostrolateral area; RSP, retrosplenial cortex; SS, somatosensory areas; TEa, temporal association areas; V1, primary visual cortex.

(B) Fraction of cells in visual areas presynaptic to AL-projecting (orange, 5 mice) and PM-projecting (green, 5 mice) LP neurons. Symbols denote individual brains (similar across all plots).

(C) Dorsal view of an example brain showing cells presynaptic to AL-projecting LP neurons in deep cortical layers.

(D) Average relative density of cells presynaptic to AL-projecting (top) or PM-projecting (bottom) LP neurons per volume in deep cortical layers.

(E) Fraction of presynaptic cells in four cortical visual areas divided by layer.

See also Figures S2 and S3.

session; Figure 3O) were strongly inhibited (46.8% ± 21.1% decrease in response amplitude; Figure 3L left example), indicating that a subset of LP neurons was strongly modulated by the activity in their target area. Together, these results indicate that transthalamic pathways integrate signals from V1 with information from higher visual areas.

Thalamic and cortical inputs convey distinct visual information to higher visual areas

The above results indicate that LP neurons projecting to higher visual areas receive prominent feedforward input from V1. These feedforward transthalamic pathways parallel the direct feedforward intracortical projections from V1 to higher visual areas. However, it is unknown if intracortical and transthalamic pathways are functionally distinct or convey similar information to a cortical target area. To address this question, we used *in vivo* two-photon microscopy in awake, head-fixed mice and imaged calcium signals of axonal projections from either LP or V1 expressing the calcium indicator GCaMP6f (Chen et al., 2013) within higher visual areas (Figures 4A–4H, S5A, and S5B). We extracted fluorescence signals from micrometer-sized regions in cortical layer 1, corresponding to putative axonal boutons, and inferred spiking probability from calcium transients (Figures 4B–4D; see STAR Methods; Glickfeld et al., 2013; Petreanu et al., 2012; Roth et al., 2016).

We first determined the visual response properties of LP boutons recorded in AL by presenting drifting gratings with different spatial and temporal frequencies and of varying orientations (Figures 4C and 4D). To visualize the spatiotemporal tuning of LP input to AL at the population level, we averaged the *Z*-scored responses of all single boutons to each combination of spatial and temporal grating frequency at their preferred grating direction, resulting in a spatial and temporal frequency population response matrix (Figure 4E, left). We then compared the population response of LP boutons to that of direct intracortical projections from V1 by measuring visual responses of V1 boutons in the same cortical area AL (Figure 4E, right). Population response matrices of LP and V1 boutons were markedly different. LP population activity was strongest in response to stimuli with low spatial and high temporal frequency, while V1 population activity was less specific, responding to a wider range of stimuli. To determine whether this difference was apparent at the single-bouton level, we fitted bouton responses with a Gaussian process regression model (see STAR Methods; Kim et al., 2018). We thus obtained single-bouton tuning curves for temporal and spatial frequencies as well as for the ratio between the two, the speed of the grating drift (Figure 4D, bottom). Both V1 and LP boutons in AL had diverse spatiotemporal frequency preferences. LP boutons in AL preferred stimuli with lower spatial frequency, higher temporal frequency, and therefore higher speed than V1 boutons (Figure 4F; all p values $< 10^{-50}$, Wilcoxon rank-sum test, all p values for data grouped by recording session < 0.01; see Figures S5F–S5H). LP thus conveys specific visual information to AL, different from the visual signals carried by V1 projections to the same cortical area.

To determine if the above results were specific for cortical area AL or if transthalamic and intracortical pathways are in general functionally distinct, we repeated our experimental protocol while imaging LP and V1 boutons in higher visual area PM. We found that the population response of LP boutons in PM was again different from that of V1 boutons recorded in the same area (Figure 4G). Individual LP boutons responded best to stimuli of higher temporal

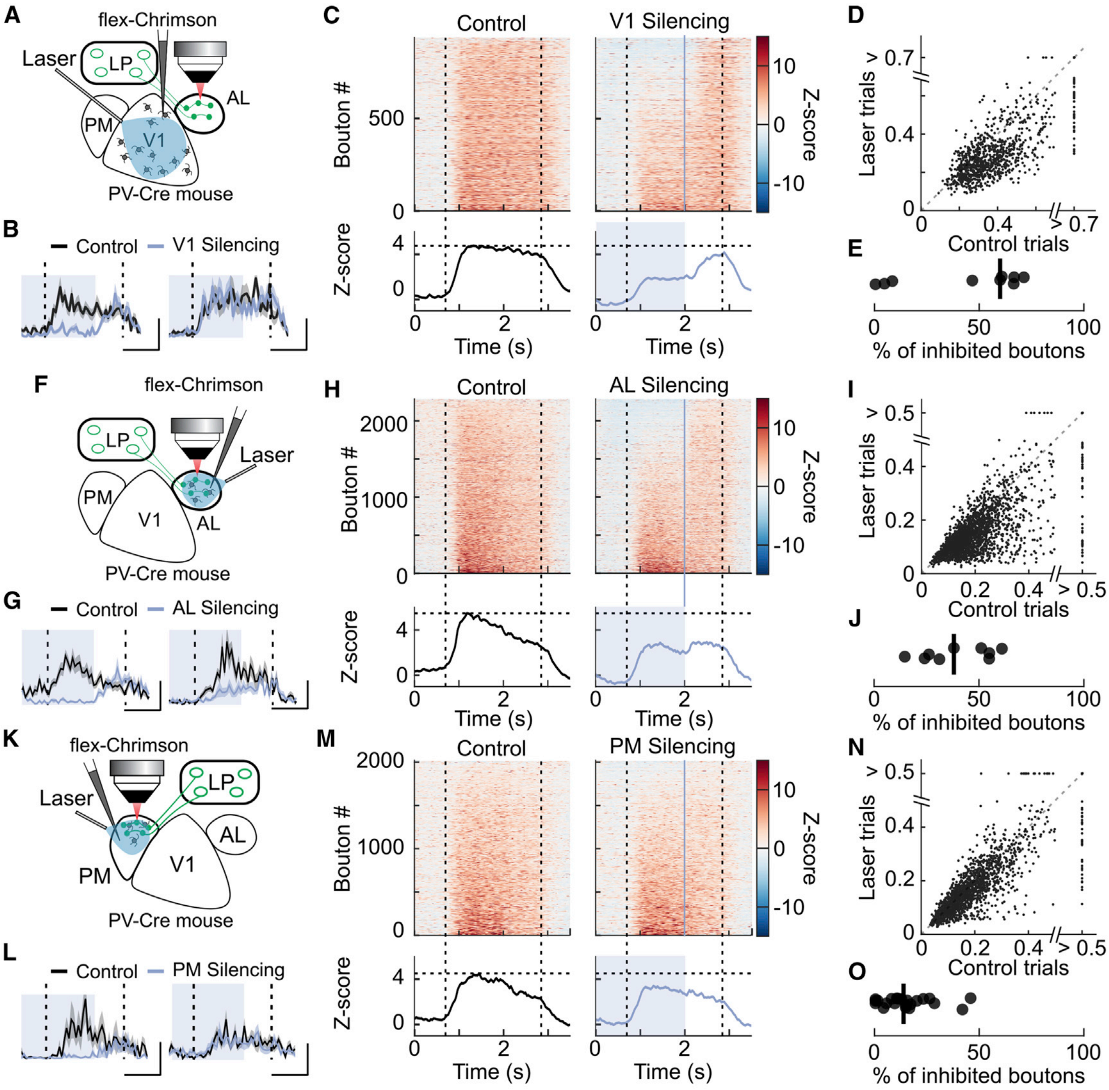

Figure 3. Functional influence of cortical inputs on LP pathways

(A) Schematic of the experimental design to suppress local cortical activity in V1 while imaging LP boutons in AL.

(B) Average responses of two example LP boutons in control trials (black) and during V1 silencing (blue). Here, and in all panels, dotted vertical lines indicate the duration of grating presentation, and blue shading indicates the time of optogenetic activation. Gray shading indicates SEM. Scale bars: left, 1 s and 0.5 a.u.; right, 1 s and 0.6 a.u.

(C) Top: time course of the *Z*-scored activity of individual LP boutons. For each bouton, activity was averaged across grating stimuli evoking a response (see STAR Methods) in control trials (left) and V1 silencing trials (right). The blue line indicates the end of optogenetic activation. Bottom: averaged *Z*-scored activity across all boutons.

(D) Relationship between the average visual responses of individual boutons with and without V1 silencing (932 boutons from 8 sessions in 3 mice).

(E) Percentage of boutons significantly inhibited per session. Black dots indicate individual recording sessions.

(F) Schematic of the experimental design as in (A) to suppress local cortical activity in AL while imaging LP boutons in AL.

(G–J) Same as (B)–(E) but for LP boutons recorded in AL during AL silencing. 2,287 boutons from 9 sessions in 6 mice; scale bars in (G): left, 1 s and 0.2 a.u.; right, 1 s and 0.5 a.u.

(K) Schematic of the experimental design as in (A) to suppress local cortical activity in PM while imaging LP boutons in PM.

(L–O) Same as (A)–(E) but for LP boutons recorded in PM during PM silencing. 1,987 boutons from 20 sessions in 12 mice; scale bars in (L): left, 1 s and 0.2 a.u.; right, 1 s and 0.4 a.u.

See also Figures S4 and S6.

frequency, lower spatial frequency, and higher speed than V1 boutons in the same area (Figure 4H; all p values < 10^{-30}, all p values for data grouped by recording session < 0.04; see Figures S5F–S5H), similar to what we had observed for LP and V1 boutons in AL. Therefore, cortical and thalamic inputs convey distinct visual information to the same higher visual area.

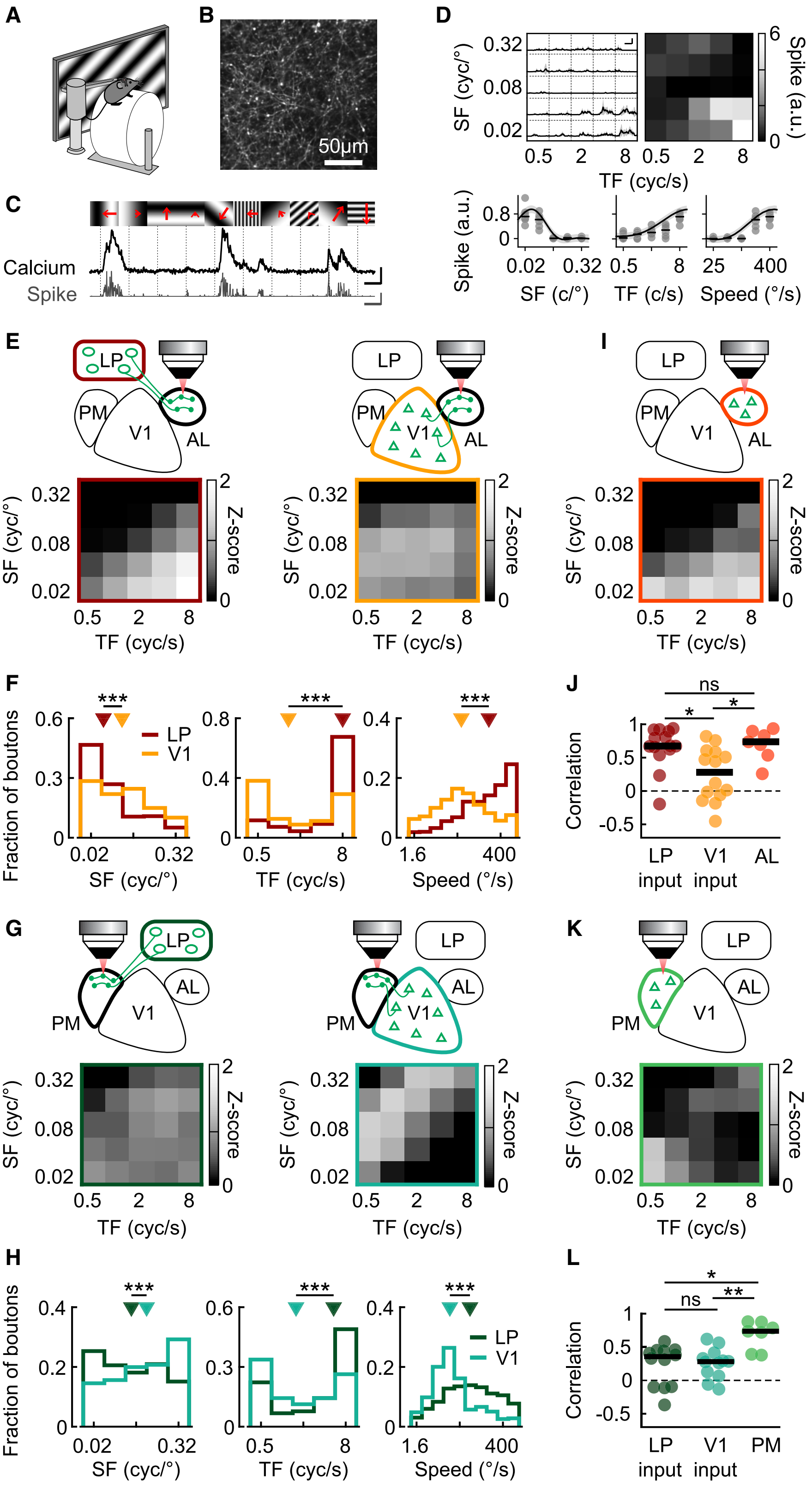

Figure 4. Thalamic and cortical inputs convey distinct visual information to higher visual areas

(A) Schematic of the experimental design.

(B) Example image of GCaMP6f-expressing LP axons in AL.

(C) Top: gratings of different orientations and spatial frequencies (SFs) were presented at various temporal frequencies (TFs; length of the red arrows). Bottom: example ΔF/F traces (black, scale bars represent 200%, 2 s) and inferred spike rate (gray, scale bars represent 2.5 a.u., 2 s).

(D) Top left: mean inferred spike rate across trials for the bouton shown in (C). Gray shading represents SD. Scale bar, 1 s and 1 a.u. Top right: response matrix obtained by averaging grating responses. Bottom: SF (left), TF (middle), and speed (right, ratio between TF and SF) response curves of the same bouton. Gray dots represent single trials, black dashes depict medians, and black curves and gray shading are predictions from the Gaussian process (GP) fit of responses and their SD (see STAR Methods).

(E) Top: schematic of the experimental design to image LP boutons (left) or V1 boutons (right) in AL. Bottom: matrix of average population responses to gratings of different TFs (x axis) and SFs (y axis) of LP boutons (left) and V1 boutons (right) in AL. 3,732 and 3,371 boutons from 14 and 14 sessions in 14 and 7 mice for LP and V1 boutons, respectively.

(F) Distribution of preferred SF (left, 2,237 LP and 2,555 V1 boutons modulated by SF), preferred TF (middle, 1,333 LP and 1,637 V1 boutons modulated by TF), and preferred speed (2,468 LP and 2,928 V1 boutons modulated by speed) of LP boutons (dark red) and V1 boutons (yellow) recorded in AL. Triangles indicate medians. All p values $< 10^{-50}$.

(G) Same as (E) but for LP boutons (left) or V1 boutons (right) in PM; 3,361 and 3,235 boutons, from 12 and 12 sessions in 10 and 7 mice for LP and V1, respectively.

(H) Distribution of preferred SF (left, 2,059 LP and 2,327 V1 boutons), preferred TF (1,128 LP and 1,659 V1 boutons) and preferred speed (2,342 LP and 2,535 V1 boutons) of LP boutons (dark green) and V1 boutons (blue) recorded in PM. Triangles indicate medians. All p values $< 10^{-30}$.

(I) Matrix of average population responses to gratings of different TF (x axis) and SF (y axis) of neurons recorded in AL (265 neurons from 7 sessions in 5 mice).

(J) Pearson correlation coefficient between the average response matrix of the population of AL neurons shown in (I) and those of individual recording sessions of LP boutons in AL (dark red), V1 boutons in AL (yellow) and AL neurons (orange). Circles represent individual recording sessions. ns, nonsignificant, $p = 0.7$;$^*p < 0.01$.

(K) Same as (I) for neurons recorded in PM (341 neurons from 8 sessions in 5 mice).

(L) Same as (J) for the correlation between the average response matrix of PM neurons (K) and those of individual recording sessions of LP boutons in PM (dark green), V1 boutons in PM (blue), and PM neurons (green). ns, nonsignificant, $p = 0.7$;$^*p < 0.01$; $^{**}p < 0.005$.

See also Figures S5 and S6.

LP projections to different visual areas consistently preferred higher temporal and lower spatial frequency stimuli than V1 projections. However, the information conveyed by LP projections was nevertheless specific to their cortical target area, as LP boutons in AL and PM showed significantly different visual response properties. LP boutons in AL preferred gratings of higher temporal frequency, higher speed, and lower spatial frequency than LP boutons in PM (Figure S5C; all p values < 10^{-20}, all p values for data grouped by recording session < 0.007; see Figures S5F–S5H). Moreover, the selectivity of LP bouton responses to different stimulus features, quantified by measuring the widths of response tuning curves, was not systematically broader than that of V1 boutons or neurons recorded in the same area (Figures S5I–S5K). For instance, LP boutons in AL were more sharply tuned for temporal frequency than V1 boutons in AL and as sharply tuned as neurons in AL (Figures S5I and S5K). These results indicate that different higher visual areas receive distinct and specific information from LP, tuned to particular visual features.

AL neurons and thalamic inputs to AL share similar response properties

Our results show that transthalamic and intracortical pathways converging onto the same visual area carry distinct visual information. This raises the question of how the response properties of these two pathways relate to those of their target areas. We therefore measured visual response properties of neurons in layers 2/3 of cortical areas AL and PM and compared them to the properties of LP and V1 inputs to these areas. Surprisingly, in area AL, the population response of cortical neurons to gratings of different spatial and temporal frequencies (Figure 4I) was more similar to LP than to V1 input (Figure 4E). We quantified this similarity as the correlation coefficient between the average population response matrix of all AL neurons and that of individual recording sessions of AL neurons, LP boutons or V1 boutons (Figure 4J). The correlation between the population response of LP boutons in different recording sessions, and the average AL population response matrix was high (Figure 4J; 0.67 ± 0.24 [median ± interquartile range]), in fact as high as when comparing individual recording sessions of AL neurons with the AL population average (Figure 4J; 0.74 ± 0.25; LP versus AL, p = 0.7). In contrast, the population responses of V1 boutons in AL were poorly correlated with the AL population response matrix (Figure 4J; 0.28 ± 0.61; AL versus V1: p = 0.01, LP versus V1: p = 0.003). Analyzing response properties of individual boutons and neurons revealed that AL neurons and LP boutons in AL were particularly well matched in their spatial frequency preferences, while AL neurons showed distributions of temporal frequency and speed preferences that lay in between distributions of V1 and LP boutons in AL (Figures S5D and S5F–S5H). These results suggest that while the total population response in AL is better matched to LP input than to V1 input, AL is likely to integrate information from both LP and V1.

Such potential integration of thalamic and cortical inputs by their target area was also apparent in PM. Population responses of PM neurons diverged from both LP and V1 input but encompassed aspects of both (Figures 4G, 4K, and 4L). Indeed, the average PM population response matrix was similarly weakly correlated with the population responses of V1 and LP boutons in PM (Figure 4L; V1: 0.28 ± 0.24; LP: 0.36 ± 0.54, p = 0.7). In both AL and PM, LP input consistently carried visual information about higher temporal frequencies, while V1 input conveyed information about higher spatial frequencies relative to their cortical target areas. These distinct signals from thalamic and intracortical projections may be combined in various ways by different higher visual areas in the cortex.

LP neurons do not inherit their tuning from their target area

Our results show that population responses of LP boutons in AL are close to those of their target area AL (Figures 4E and 4I). This could be a result of particularly strong reciprocal loops between these areas, such that response properties of LP neurons projecting to AL are inherited from AL. However, the response properties of LP boutons in AL were not altered when AL was silenced (Figures S6A–S6F). Boutons whose responses were suppressed during AL silencing showed residual population responses, response preferences, and response specificity similar to their responses without silencing (Figures S6A–S6F). Furthermore, population responses of LP boutons suppressed by AL silencing were not different from the rest of the population in control trials without optogenetic stimulation (Figures S6E and S6F). Therefore, while AL exerts a strong influence on LP activity, the visual response properties of LP neurons projecting to AL are not only inherited from AL but closely resemble responses of AL neurons, even when the input from this cortical area is removed. We obtained relatively similar results during V1 silencing (Figures S6G–S6L). Population responses and response specificity of LP boutons in AL were unchanged when V1 was silenced (Figures S6J–S6L), while the distributions of individual LP bouton response preferences were only slightly altered in suppressed LP boutons (Figures S6H and S6I). These results indicate that inputs to LP neurons from different brain areas are matched in their visual response properties.

Thalamic and cortical inputs convey distinct visuomotor information to higher visual areas

Our results indicate that LP is a key node in transthalamic pathways that send specific visual information to higher visual cortical areas. Other studies additionally suggest that transthalamic pathways convey nonvisual information (Komura et al., 2013; Roth et al., 2016; Saalmann and Kastner, 2015). LP may therefore integrate visual with contextual information, for instance about an animal's own actions. To explore this possibility, we used two-photon calcium imaging of LP boutons in AL and compared their responses to those of V1 boutons imaged in the same cortical area in head-fixed mice running on a cylinder through a virtual environment (Figure 5). We habituated mice to a virtual linear corridor, where the motion of visual patterns displayed on monitors was controlled by the running speed of the animal, similar to previous studies (Poort et al., 2015; Roth et al., 2016). During calcium recordings, we then uncoupled the virtual optic flow from the animals' locomotion by replaying movies of the virtual corridor recorded in previous sessions (see STAR Methods). This allowed us to separately assess the effect of locomotion and visual motion on neuronal activity

CellPress
OPEN ACCESS

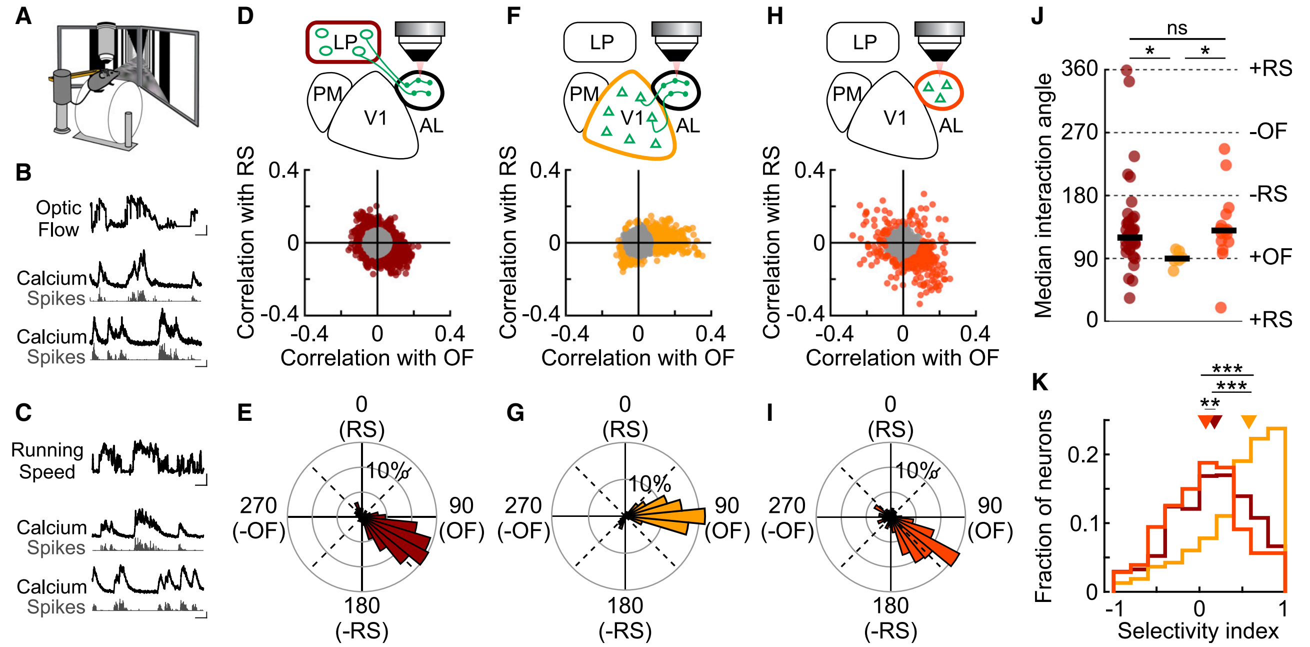

Figure 5. Thalamic and cortical inputs convey distinct visuo-motor information to higher visual areas

(A) Schematic of the experimental design to allow uncoupling of optic flow feedback from the animal's locomotion while recording neuronal responses.

(B) Top trace: optic flow speed (OF); below: example activity traces of two AL neurons positively (middle; correlation coefficient R = 0.16) and negatively correlated (bottom; R = −0.23) with OF. Black, calcium trace; gray, inferred spikes. Scale bar for OF represents 5 s and 10 cm/s, scale bar for neuronal activity represents 5 s and 100% ΔF/F/ 1 a.u.

(C) Same as (B) for running speed (RS). Example of positively (middle; R = 0.24) and negatively correlated (bottom; R = −0.23) neurons with RS.

(D) Top: schematic of the recording configuration. Bottom: relationship between the mean cross-correlation coefficients (see STAR Methods) of neuronal activity with RS and OF for all responsive LP boutons imaged in AL (12,369 boutons from 43 sessions in 18 mice). Only boutons with mean cross-correlation greater than 0.1 (colored points in scatterplot) were included in the analysis shown in (E), (J), and (K).

(E) Histogram in polar coordinates showing the distribution of interaction angles between the mean cross-correlation of activity with RS and OF for LP boutons imaged in AL (919 boutons from 43 sessions in 18 mice).

(F) Same as (D) for V1 boutons imaged in AL (2,363 boutons from 6 sessions in 3 mice).

(G) Same as (E) for V1 boutons imaged in AL (338 boutons from 6 sessions in 3 mice).

(H) Same as (D) for cortical neurons imaged in AL (735 neurons from 15 sessions in 5 mice).

(I) Same as (E) for cortical neurons imaged in AL (289 neurons from 15 sessions in 5 mice).

(J) Median interaction angles across single sessions for LP boutons in AL (left), V1 boutons in AL (middle), and AL neurons (right). Black horizontal lines represent the circular median across sessions. *$p < 0.01$ Circular nonparametric multi-sample test for equal medians; ns, nonsignificant, $p = 0.13$.

(K) Distribution of selectivity indices (see STAR Methods) for individual LP boutons in AL (dark red), AL neurons (orange), and V1 boutons in AL (yellow). −1 indicates a high correlation only with RS, 1 indicates a high correlation only with OF, and 0 indicates equally high correlation with RS and OF. **$p < 10^{-3}$; ***$p < 10^{-27}$.

See also Figure S8.

(Figures 5B and 5C). We quantified how strongly neuronal activity inferred from calcium signals was modulated by either running speed or optic flow by computing mean cross-correlation coefficients between neuronal activity and these variables for each bouton (Figure S7; see STAR Methods). These correlation coefficients revealed diverse relationships, including positive and negative correlations (Figures 5B and 5C).

To estimate the degree to which information about visual motion and running speed is integrated at the level of individual boutons, we plotted the mean correlation coefficients between neuronal activity and the two variables against each other for each bouton (Figures 5D and 5F). For boutons carrying information about running speed or optic flow (mean cross-correlation coefficient ≥ 0.1), we then derived an interaction angle θ (see STAR Methods). Values of θ close to 0° indicate a bouton not modulated by optic flow speed but positively correlated with running speed, increasing its responses with increasing running speed. Values close to 180° indicate a bouton whose activity was negatively correlated with running speed and decreased its responses with increasing running speed. Accordingly, θ values of 90° or 270° indicate that a bouton was not modulated by running speed, but its activity was positively or negatively correlated with optic flow speed, respectively. Finally, oblique angles correspond to neurons informative about both variables.

The large majority of V1 boutons recorded in cortical area AL had interaction angles of ~90°, denoting that their activity was positively correlated with optic flow speed but was not correlated with running speed (Figure 5G). Therefore, while the activity of V1 neurons has been shown to be modulated by locomotion (Niell and Stryker, 2010), under our experimental conditions, V1

projections to AL convey mainly visual information about the speed of visual motion. In contrast, the activity of many LP boutons was modulated both by optic flow and running speed. Neuronal activity in these boutons was predominantly positively correlated with optic flow speed and negatively correlated with running speed, indicating that they were activated by visual motion but suppressed by running (Figures 5D and 5E). Accordingly, LP boutons exhibited mainly interaction angles between 90° and 180°, significantly different from the distribution of angles of V1 boutons (Figure 5J). These data suggest that while V1 projections to AL mostly provide a channel for visual information, LP neurons projecting to AL are informative about both optic flow and running speed. To explicitly depict the degree to which different boutons integrate visual and motor signals, we computed a selectivity index, where values of −1 or 1 denote boutons modulated by only one variable (running or optic flow speed, respectively), while values close to 0 denote that the activity of a bouton was equally well correlated with both variables (Figure 5K; see STAR Methods). As expected, selectivity indices of LP boutons in AL were distributed around 0 (median 0.18), significantly different from the distribution of V1 boutons in AL, which was biased toward 1 (median 0.58, $p < 10^{-27}$). Therefore, V1 and LP convey different information to higher visual area AL in behaving animals; while V1 projections carry predominantly optic flow signals, the transthalamic pathway integrates these visual motion signals with information about the animals' own movement speed.

In addition, we performed similar analyses on neurons in cortical area AL recorded during the same experimental conditions. The activity of most AL neurons was modulated by both optic flow and running speed, similar to their inputs from LP, but not from V1 (Figures 5H–5K). Notably, these visuo-motor signals were very distinct from the activity we observed in neurons in cortical area PM and its inputs from LP and V1, which showed a wide variety of visual and motor-related signals (Figure S8). Only AL neurons and LP boutons in AL both showed predominantly positive correlations with optic flow speed but negative correlations with running speed, implying that they are activated by visual flow but suppressed by locomotion. These response characteristics could give rise to the suppression of running-induced optic flow, suggesting that visual area AL and LP-to-AL thalamocortical circuits may be specialized to process visual motion relative to self-motion.

DISCUSSION

We studied the anatomical and functional organization of higher-order thalamic circuits in the visual system. We found that LP neurons are part of feedforward transthalamic pathways that integrate signals from V1 with input from a large number of cortical and subcortical regions. These pathways convey target-specific visuo-motor information to different higher visual areas, distinct from the visual signals carried by V1 projections to the same cortical target, highlighting the functional difference between transthalamic and intracortical pathways.

LP is a key node of feedforward transthalamic pathways

LP neurons projecting to cortical areas AL or PM formed largely segregated populations, yet they received relatively similar distributions of inputs from the same brain areas according to monosynaptic rabies tracing. Both populations of LP projection neurons received input from all higher visual cortical areas, in particular from layer 6 cells. Axons from layer 6 cells form small synapses contacting distal dendrites in the thalamus and are thought to have a modulatory effect on thalamic neurons (Alitto and Usrey, 2003; Bickford, 2016; Li et al., 2003; Rockland, 1996; Sherman, 2016; Sherman and Guillery, 2011). In contrast, cortical layer 5 cells form large synapses on proximal dendrites of higher-order thalamic neurons (Bickford, 2016; Groh et al., 2014; Li et al., 2003; Mathers, 1972), evoking large postsynaptic currents that can strongly influence action potential firing (Groh et al., 2008, 2014; Reichova and Sherman, 2004). Accordingly, layer 5 cells are likely to provide the main driving input from the cortex to higher-order thalamus. By far the largest number of presynaptic layer 5 cells of both AL- and PM-projecting LP neurons were located in V1, indicating that transthalamic pathways through LP encompass a prominent feedforward component. This was corroborated by the strong influence exerted by V1 on the visual responses of thalamocortical projections to higher visual areas. Together, our results support the long-standing hypothesis that sensory higher-order thalamic circuits form indirect feedforward pathways (Sherman, 2016; Sherman and Guillery, 2011). However, our findings also indicate that these feedforward thalamocortical pathways are significantly affected by layer 6 corticofugal projections from higher visual areas and likely integrate information from many sources.

The observed effect of V1 manipulation on LP activity is likely an underestimation, since because of its large size (surface area > 4 mm^2), we probably only silenced parts of V1 (Chrimson was delivered via AAV injections and light through one 200- to 400-μm-diameter fiber). This may have also been the case for area PM, as Chrimson was only expressed in frontal PM (targeting of injections similar to Figure S1D). In contrast, optogenetic activation of parvalbumin-positive interneurons in the small cortical area AL (~0.4 mm^2) likely also suppressed activity in surrounding cortical areas (Li et al., 2019), including small parts of LM and V1.

Functional specificity of transthalamic pathways

LP conveys functionally distinct and specific information to cortical areas AL and PM. Therefore, even though some LP neurons have very large axonal projection fields (Clascá et al., 2016; Nakamura et al., 2015), LP thalamocortical projections do not broadcast identical, nonspecific signals across the cortex. These pathways carry specific visual information, tuned to spatial and temporal attributes of the visual input. For most stimulus parameters, the average tuning widths of LP boutons were not broader than those of V1 boutons or neurons in higher visual areas. This contrasts with LP projections to V1, which convey less selective signals about the visual scene (Roth et al., 2016) and may therefore constitute a modulatory feedback pathway. While LP neurons projecting to different cortical target areas have distinct response properties, they integrate information from the same set of cortical and subcortical areas. Thus, they likely receive input from distinct sets of neurons within those areas, in particular in V1. Similarly, V1 neurons projecting to areas AL and PM constitute separate populations with distinct response properties (Glickfeld et al., 2013; Kim et al., 2018).

Nevertheless, most LP neurons likely project to multiple cortical areas (Clascá et al., 2016; Juavinett et al., 2020; Nakamura et al., 2015), resulting in combinatorial distribution of information along thalamocortical pathways. Determining the detailed projection motifs of single LP neurons with high-throughput methods, for instance using genetic barcoding and *in situ* sequencing (Chen et al., 2019), will be an essential future step toward a better understanding of higher-order thalamocortical processing.

Transthalamic and intracortical pathways carry distinct information to the same target area

We find that transthalamic feedforward pathways through LP convey information distinct from that carried by direct cortical projections from V1. LP and higher visual areas are innervated by separate populations of V1 cells (pyramidal tract and intratelencephalic neurons, respectively), with potentially different functional properties (Harris and Mrsic-Flogel, 2013). Importantly, LP neurons receive input from many other cortical and subcortical areas, notably from the superior colliculus. The superior colliculus provides particularly dense, driving input to the caudal part of rodent LP, which is mainly conveyed to lateral visual cortical areas (Beltramo and Scanziani, 2019; Bennett et al., 2019; Zhou et al., 2017), forming a second feedforward visual pathway from retina to cortex (Beltramo and Scanziani, 2019). Our study focused mainly on the anterior-lateral part of LP (Baldwin et al., 2017; Bennett et al., 2019; Zhou et al., 2017), which is innervated by the ipsilateral superior colliculus (Zhou et al., 2017; see also Figure 1) but receives its main input from visual cortical areas, providing an ideal substrate to integrate information from cortical and midbrain sources.

In our study, LP projections to higher visual areas showed a notable preference for visual stimuli with high temporal frequencies and high speed compared to V1 projections. A previous study suggested that responses to high-velocity stimuli in higher visual areas are decreased after lesioning superior colliculus (Tohmi et al., 2014), indicating that the preference of LP neurons for high temporal frequency and speed could, at least in part, be inherited from the superior colliculus and may in turn influence responses in higher visual areas. This visual channel for high temporal frequency information provided by the transthalamic LP pathway is particularly well matched with response properties of cortical area AL. AL is strongly activated by visual motion (Orsolic et al., 2021; de Vries et al., 2020) and may be specialized to process visual motion of higher temporal frequencies (Andermann et al., 2011; Marshel et al., 2011). It may therefore rely particularly strongly on the input from LP. In contrast, projections from V1 provide a visual channel with higher spatial resolution, which may be more relevant for cortical area PM, which preferentially processes visual stimuli with high spatial frequency (Andermann et al., 2011; Marshel et al., 2011; Roth et al., 2012). Accordingly, our data suggest that higher visual areas integrate cortical and thalamic information differently depending on their function.

Recent studies showed that input from higher-order thalamus is critical for driving activity in motor cortex and postrhinal visual cortex (Beltramo and Scanziani, 2019; Guo et al., 2017; Sauerbrei et al., 2020). To what degree pulvinar and LP affect activity in other higher visual areas is still debated (Minville and Casanova, 1998; Soares et al., 2004; de Souza et al., 2020; Zhou et al., 2016), and further studies with silencing of specific LP pathways are crucial to resolve this question.

Transthalamic pathways through LP integrate visual and contextual information

LP conveys not only visual signals to the cortex but also contextual information about the animals' movement. While intracortical projections from V1 to AL carried mainly visual information about optic flow speed in animals traversing a virtual corridor, projections from LP to AL showed responses that integrated these visual signals with information about the animals' running speed. Such motor information could originate from superior colliculus or secondary motor and anterior cingulate cortex (Leinweber et al., 2017). Optic flow and running speed had opposing effects on the activity of LP projections to AL. These projections could therefore signal discrepancies between the expected optic flow based on the animal's movement and the actual visual motion in the environment. Indeed, in an earlier study, we showed that LP neurons projecting to V1 preferentially compute the degree of difference between running and optic flow speed (Roth et al., 2016). LP neurons projecting to V1 increase their responses with locomotion and are suppressed by optic flow. They are thus most active when an animal is running but the optic flow stops, similar to the response characteristics of a subset of V1 neurons (Keller et al., 2012). In contrast, neurons in AL and LP projections to AL are suppressed by locomotion and activated by optic flow, suggesting they contribute to processing of visual motion relative to self-motion. These neurons would be most active when the speed of optic flow is higher than expected based on the animal's running speed or when visual stimuli move in the environment while the animal is stationary. Therefore, LP-AL circuits could be specialized to distinguish external visual stimuli from self-generated visual feedback.

Our results indicate that sensory thalamocortical circuits are important to integrate cortical information with subcortical signals, in particular from the midbrain. They can thus contribute additional information that is relevant for a specific cortical area, but not already present in neocortical circuits, about both specific aspects of a sensory stimulus and the behavioral context it is encountered in. Moreover, information about the behavioral relevance of a stimulus and the animal's priorities, prominently represented in the superior colliculus (Basso and May, 2017; Krauzlis et al., 2013), could thus be combined with visual information from the cortex and regulate activity in higher visual areas accordingly. This hypothesis is in keeping with previous reports of a variety of nonsensory signals in pulvinar neurons, such as the animal's focus of attention or its uncertainty about the stimulus content (Halassa and Kastner, 2017; Komura et al., 2013; Saalmann et al., 2012; Zhou et al., 2016).

Pulvinar circuits have been hypothesized to regulate communication between cortical areas (Halassa and Kastner, 2017; Jaramillo et al., 2019; Saalmann et al., 2012). Our data confirm that pulvinar circuits connect different cortical areas and show that these cortico-thalamo-cortical pathways concurrently receive signals from many other visual and nonvisual brain regions. Furthermore, several long-range inhibitory circuits provide input to pulvinar neurons (Figures 1G and 1H) that may have the capacity to differentially regulate specific transthalamic pathways

(Crabtree, 2018; Halassa and Acsády, 2016; Trageser and Keller, 2004) and thereby affect cortical information processing. We propose that higher-order thalamic circuits can both regulate sensory processing within cortical areas and control information transfer between areas (Halassa and Kastner, 2017), depending on the nature of sensory input, the behavioral circumstances, and the animal's behavioral state.

STAR★METHODS

Detailed methods are provided in the online version of this paper and include the following:

- KEY RESOURCES TABLE
- RESOURCE AVAILABILITY
  - Lead contact
  - Materials availability
  - Data and code availability
- EXPERIMENTAL MODEL AND SUBJECT DETAILS
- METHOD DETAILS
  - Surgical procedures and virus injections
  - Intrinsic signal imaging
  - Histology
  - Two-photon calcium imaging
  - Visual stimulation
  - Visual response characterization
  - Visuo-motor response characterization
  - Optogenetic manipulation
  - Electrophysiology
- QUANTIFICATION AND STATISTICAL ANALYSIS
  - CTB and mono-synaptic rabies tracing
  - Two-photon imaging
  - Electrophysiology
  - Analysis of visual responses
  - Analysis of visuo-motor responses
  - Statistics

SUPPLEMENTAL INFORMATION

Supplemental information can be found online at https://doi.org/10.1016/j.neuron.2021.04.017.

ACKNOWLEDGMENTS

We thank Tom Otis, Alison Barth, Maxime Rio, Petr Znamenskiy, Thomas Mrsic-Flogel, Andreas Keller, and Riccardo Beltramo for discussions and comments on the manuscript. We thank Sara Mederos, Rob Campbell, and Alice Koltchev for help with data acquisition; Mean-Hwan Kim and Petr Znamenskiy for sharing data from CTB injection experiments; Troy Margrie and Adam Tyson for sharing the cellfinder software; Andrew Murray and Molly Strom for providing rabies and AAV helper viruses; Michelle Li for animal husbandry; and Maxime Rio, Petr Znamenskiy, and Dylan Muir for developing software. This work was supported by the Sainsbury Wellcome Centre Core Grant from the Gatsby Charitable Foundation and Wellcome (090843/F/09/Z), an ERC Starting Grant (S.B.H., HigherVision 337797), and an SNSF Project Grant (S.B.H., 31003A_169525).

AUTHOR CONTRIBUTIONS

S.B.H., M.M.R., and A.B. designed the experiments. I.G., F.I., and M.J. performed pilot experiments. I.G. performed rabies tracing experiments, and A.B. conducted post-hoc tissue processing and analysis and electrophysiological recordings. A.B., M.R., and I.G. conducted two-photon imaging and optogenetic experiments. A.B. and M.M.R. analyzed the data with help from I.G. S.B.H., M.M.R., and A.B. wrote the manuscript with help from I.G.

DECLARATION OF INTERESTS

The authors declare no competing interests.

Received: July 1, 2020
Revised: February 28, 2021
Accepted: April 15, 2021
Published: May 11, 2021

REFERENCES

Alitto, H.J., and Usrey, W.M. (2003). Corticothalamic feedback and sensory processing. Curr. Opin. Neurobiol. *13*, 440–445.

Andermann, M.L., Kerlin, A.M., Roumis, D.K., Glickfeld, L.L., and Reid, R.C. (2011). Functional specialization of mouse higher visual cortical areas. Neuron *72*, 1025–1039.

Attinger, A., Wang, B., and Keller, G.B. (2017). Visuomotor coupling shapes the functional development of mouse visual cortex. Cell *169*, 1291–1302.e14.

Baldwin, M.K.L., Balaram, P., and Kaas, J.H. (2017). The evolution and functions of nuclei of the visual pulvinar in primates. J. Comp. Neurol. *525*, 3207–3226.

Basso, M.A., and May, P.J. (2017). Circuits for action and cognition: a view from the superior colliculus. Annu. Rev. Vis. Sci. *3*, 197–226.

Beltramo, R., and Scanziani, M. (2019). A collicular visual cortex: Neocortical space for an ancient midbrain visual structure. Science *363*, 64–69.

Bennett, C., Gale, S.D., Garrett, M.E., Newton, M.L., Callaway, E.M., Murphy, G.J., and Olsen, S.R. (2019). Higher-order thalamic circuits channel parallel streams of visual information in mice. Neuron *102*, 477–492.e5.

Berens, P. (2009). CircStat: A MATLAB Toolbox for Circular Statistics. J. Stat. Softw. *31*, 1–21.

Bickford, M.E. (2016). Thalamic circuit diversity: modulation of the driver/modulator framework. Front. Neural Circuits *9*, 86.

Campbell, R. (2020). SainsburyWellcomeCentre/BakingTray: Jan 2020.. https://zenodo.org/record/3631610#.YIh6f7VKiUk.

Caswell, T.A., Droettboom, M., Hunter, J., Firing, E., Lee, A., Klymak, J., Stansby, D., Sales de Andrade, E., Hedegaard Nielsen, J., Varoquaux, N., et al. (2019), v3.1.0 https://matplotlib/matplotlib.

Chabrol, F.P., Blot, A., and Mrsic-Flogel, T.D. (2019). Cerebellar contribution to preparatory activity in motor neocortex. Neuron *103*, 506–519.e4.

Chen, T.-W., Wardill, T.J., Sun, Y., Pulver, S.R., Renninger, S.L., Baohan, A., Schreiter, E.R., Kerr, R.A., Orger, M.B., Jayaraman, V., et al. (2013). Ultrasensitive fluorescent proteins for imaging neuronal activity. Nature *499*, 295–300.

Chen, X., Sun, Y.-C., Zhan, H., Kebschull, J.M., Fischer, S., Matho, K., Huang, Z.J., Gillis, J., and Zador, A.M. (2019). High-throughput mapping of long-range neuronal projection using in situ sequencing. Cell *179*, 772–786.e19.

Clascá, F., Porrero, C., Galazo, M.J., Rubio-Garrido, P., and Evangelio, M. (2016). Anatomy and Development of Multispecific Thalamocortical Axons. In Axons and Brain Architecture (Elsevier), pp. 69–92.

Crabtree, J.W. (2018). Functional diversity of thalamic reticular subnetworks. Front. Syst. Neurosci. *12*, 41.

Crick, F., and Koch, C. (1998). Constraints on cortical and thalamic projections: the no-strong-loops hypothesis. Nature *391*, 245–250.

Dana, H., Sun, Y., Mohar, B., Hulse, B.K., Kerlin, A.M., Hasseman, J.P., Tsegaye, G., Tsang, A., Wong, A., Patel, R., et al. (2019). High-performance calcium sensors for imaging activity in neuronal populations and microcompartments. Nat. Methods *16*, 649–657.

CellPress
OPEN ACCESS

de Souza, B.O.F., Cortes, N., and Casanova, C. (2020). Pulvinar modulates contrast responses in the visual cortex as a function of cortical hierarchy. Cereb. Cortex *30*, 1068–1086.

de Vries, S.E.J., Lecoq, J.A., Buice, M.A., Groblewski, P.A., Ocker, G.K., Oliver, M., Feng, D., Cain, N., Ledochowitsch, P., Millman, D., et al. (2020). A large-scale standardized physiological survey reveals functional organization of the mouse visual cortex. Nat. Neurosci. *23*, 138–151.

Dyer, E.L., Studer, C., Robinson, J.T., and Baraniuk, R.G. (2013). A robust and efficient method to recover neural events from noisy and corrupted data. In 2013 6th International IEEE/EMBS Conference on Neural Engineering (NER), pp. 593–596.

Fang, Q., Chou, X.L., Peng, B., Zhong, W., Zhang, L.I., and Tao, H.W. (2020). A differential circuit via retino-colliculo-pulvinar pathway enhances feature selectivity in visual cortex through surround suppression. Neuron *105*, 355–369.e6.

Glickfeld, L.L., Andermann, M.L., Bonin, V., and Reid, R.C. (2013). Cortico-cortical projections in mouse visual cortex are functionally target specific. Nat. Neurosci. *16*, 219–226.

Groh, A., de Kock, C.P.J., Wimmer, V.C., Sakmann, B., and Kuner, T. (2008). Driver or coincidence detector: modal switch of a corticothalamic giant synapse controlled by spontaneous activity and short-term depression. J. Neurosci. *28*, 9652–9663.

Groh, A., Bokor, H., Mease, R.A., Plattner, V.M., Hangya, B., Stroh, A., Deschenes, M., and Acsády, L. (2014). Convergence of cortical and sensory driver inputs on single thalamocortical cells. Cereb. Cortex *24*, 3167–3179.

Guillery, R.W., and Sherman, S.M. (2002). Thalamic relay functions and their role in corticocortical communication: generalizations from the visual system. Neuron *33*, 163–175.

Guo, Z.V., Inagaki, H.K., Daie, K., Druckmann, S., Gerfen, C.R., and Svoboda, K. (2017). Maintenance of persistent activity in a frontal thalamocortical loop. Nature *545*, 181–186.

Guo, K., Yamawaki, N., Barrett, J.M., Tapies, M., and Shepherd, G.M.G. (2020). Cortico-thalamo-cortical circuits of mouse forelimb S1 are organized primarily as recurrent loops. J. Neurosci. *40*, 2849–2858.

Halassa, M.M., and Acsády, L. (2016). Thalamic inhibition: diverse sources, diverse scales. Trends Neurosci. *39*, 680–693.

Halassa, M.M., and Kastner, S. (2017). Thalamic functions in distributed cognitive control. Nat. Neurosci. *20*, 1669–1679.

Harris, K.D., and Mrsic-Flogel, T.D. (2013). Cortical connectivity and sensory coding. Nature *503*, 51–58.

Hippenmeyer, S., Vrieseling, E., Sigrist, M., Portmann, T., Laengle, C., Ladle, D.R., and Arber, S. (2005). A developmental switch in the response of DRG neurons to ETS transcription factor signaling. PLoS Biol. *3*, e159.

Hoerder-Suabedissen, A., Hayashi, S., Upton, L., Nolan, Z., Casas-Torremocha, D., Grant, E., Viswanathan, S., Kanold, P.O., Clasca, F., Kim, Y., and Molnár, Z. (2018). Subset of cortical layer 6b neurons selectively innervates higher order thalamic nuclei in mice. Cereb. Cortex *28*, 1882–1897.

Hu, F., Kamigaki, T., Zhang, Z., Zhang, S., Dan, U., and Dan, Y. (2019). Prefrontal corticotectal neurons enhance visual processing through the superior colliculus and pulvinar thalamus. Neuron *104*, 1141–1152.e4.

Jaramillo, J., Mejias, J.F., and Wang, X.-J. (2019). Engagement of pulvino-cortical feedforward and feedback pathways in cognitive computations. Neuron *101*, 321–336.e9.

Jones, E.G. (1985). The Thalamus (Springer).

Juavinett, A.L., Kim, E.J., Collins, H.C., and Callaway, E.M. (2020). A systematic topographical relationship between mouse lateral posterior thalamic neurons and their visual cortical projection targets. J. Comp. Neurol. *528*, 95–107.

Kalatsky, V.A., and Stryker, M.P. (2003). New paradigm for optical imaging: temporally encoded maps of intrinsic signal. Neuron *38*, 529–545.

Keller, G.B., Bonhoeffer, T., and Hübener, M. (2012). Sensorimotor mismatch signals in primary visual cortex of the behaving mouse. Neuron *74*, 809–815.

Kim, M.-H., Znamenskiy, P., Iacaruso, M.F., and Mrsic-Flogel, T.D. (2018). Segregated subnetworks of intracortical projection neurons in primary visual cortex. Neuron *100*, 1313–1321.e6.

Klapoetke, N.C., Murata, Y., Kim, S.S., Pulver, S.R., Birdsey-Benson, A., Cho, Y.K., Morimoto, T.K., Chuong, A.S., Carpenter, E.J., Tian, Z., et al. (2014). Independent optical excitation of distinct neural populations. Nat. Methods *11*, 338–346.

Klein, S., Staring, M., Murphy, K., Viergever, M.A., and Pluim, J.P.W. (2010). elastix: a toolbox for intensity-based medical image registration. IEEE Trans. Med. Imaging *29*, 196–205.

Kleiner, M., Brainard, D., Pelli, D., Ingling, A., Murray, R., and Broussard, C. (2007). What's new in psychtoolbox-3. Perception *36*, 1–16.

Komura, Y., Nikkuni, A., Hirashima, N., Uetake, T., and Miyamoto, A. (2013). Responses of pulvinar neurons reflect a subject's confidence in visual categorization. Nat. Neurosci. *16*, 749–755.

Krauzlis, R.J., Lovejoy, L.P., and Zénon, A. (2013). Superior colliculus and visual spatial attention. Annu. Rev. Neurosci. *36*, 165–182.

Lein, E.S., Hawrylycz, M.J., Ao, N., Ayres, M., Bensinger, A., Bernard, A., Boe, A.F., Boguski, M.S., Brockway, K.S., Byrnes, E.J., et al. (2007). Genome-wide atlas of gene expression in the adult mouse brain. Nature *445*, 168–176.

Leinweber, M., Zmarz, P., Buchmann, P., Argast, P., Hübener, M., Bonhoeffer, T., and Keller, G.B. (2014). Two-photon calcium imaging in mice navigating a virtual reality environment. J. Vis. Exp. *20*, e50885.

Leinweber, M., Ward, D.R., Sobczak, J.M., Attinger, A., and Keller, G.B. (2017). A sensorimotor circuit in mouse cortex for visual flow predictions. Neuron *95*, 1420–1432.e5.

Li, J., Guido, W., and Bickford, M.E. (2003). Two distinct types of corticothalamic EPSPs and their contribution to short-term synaptic plasticity. J. Neurophysiol. *90*, 3429–3440.

Li, N., Chen, S., Guo, Z.V., Chen, H., Huo, Y., Inagaki, H.K., Chen, G., Davis, C., Hansel, D., Guo, C., and Svoboda, K. (2019). Spatiotemporal constraints on optogenetic inactivation in cortical circuits. eLife *8*, e48622.

Marshel, J.H., Garrett, M.E., Nauhaus, I., and Callaway, E.M. (2011). Functional specialization of seven mouse visual cortical areas. Neuron *72*, 1040–1054.

Mathers, L.H. (1972). The synaptic organization of the cortical projection to the pulvinar of the squirrel monkey. J. Comp. Neurol. *146*, 43–60.

Matthews, A.G., de, G., Wilk, M., van der, Nickson, T., Fujii, K., Boukouvalas, A., León-Villagrá, P., Ghahramani, Z., and Hensman, J. (2017). GPflow: A Gaussian Process Library using TensorFlow. Journal of Machine Learning Research *18*, 1–6.

Mayerich, D., Abbott, L., and McCormick, B. (2008). Knife-edge scanning microscopy for imaging and reconstruction of three-dimensional anatomical structures of the mouse brain. J. Microsc. *231*, 134–143.

Minville, K., and Casanova, C. (1998). Spatial frequency processing in posteromedial lateral suprasylvian cortex does not depend on the projections from the striate-recipient zone of the cat's lateral posterior-pulvinar complex. Neuroscience *84*, 699–711.

Muir, D.R., Roth, M., and Blot, A. (2020). TimeSeries analysis toolbox for Matlab. https://zenodo.org/record/3859433#.Ylh8HLVKiUk.

Nakamura, H., Hioki, H., Furuta, T., and Kaneko, T. (2015). Different cortical projections from three subdivisions of the rat lateral posterior thalamic nucleus: a single-neuron tracing study with viral vectors. Eur. J. Neurosci. *41*, 1294–1310.

Niell, C.M., and Stryker, M.P. (2010). Modulation of visual responses by behavioral state in mouse visual cortex. Neuron *65*, 472–479.

Orsolic, I., Rio, M., Mrsic-Flogel, T.D., and Znamenskiy, P. (2021). Mesoscale cortical dynamics reflect the interaction of sensory evidence and temporal expectation during perceptual decision-making. Neuron, In press. https://doi.org/10.1016/j.neuron.2021.03.031.

Pachitariu, M., Steinmetz, N., Kadir, S., Carandini, M., and Harris Kenneth, D. (2016). Kilosort: realtime spike-sorting for extracellular electrophysiology with hundreds of channels. bioRxiv. https://doi.org/10.1101/061481.

Petreanu, L., Gutnisky, D.A., Huber, D., Xu, N.L., O'Connor, D.H., Tian, L., Looger, L., and Svoboda, K. (2012). Activity in motor-sensory projections reveals distributed coding in somatosensation. Nature *489*, 299–303.

Pologruto, T.A., Sabatini, B.L., and Svoboda, K. (2003). ScanImage: flexible software for operating laser scanning microscopes. Biomed. Eng. Online *2*, 13.

Poort, J., Khan, A.G., Pachitariu, M., Nemri, A., Orsolic, I., Krupic, J., Bauza, M., Sahani, M., Keller, G.B., Mrsic-Flogel, T.D., and Hofer, S.B. (2015). Learning Enhances Sensory and Multiple Non-sensory Representations in Primary Visual Cortex. Neuron *86*, 1478–1490.

Ragan, T., Kadiri, L.R., Venkataraju, K.U., Bahlmann, K., Sutin, J., Taranda, J., Arganda-Carreras, I., Kim, Y., Seung, H.S., and Osten, P. (2012). Serial two-photon tomography for automated ex vivo mouse brain imaging. Nat. Methods *9*, 255–258.

Reardon, T.R., Murray, A.J., Turi, G.F., Wirblich, C., Croce, K.R., Schnell, M.J., Jessell, T.M., and Losonczy, A. (2016). Rabies Virus CVS-N2c(ΔG) strain enhances retrograde synaptic transfer and neuronal viability. Neuron *89*, 711–724.

Reichova, I., and Sherman, S.M. (2004). Somatosensory corticothalamic projections: distinguishing drivers from modulators. J. Neurophysiol. *92*, 2185–2197.

Rockland, K.S. (1996). Two types of corticopulvinar terminations: round (type 2) and elongate (type 1). J. Comp. Neurol. *368*, 57–87.

Roth, M.M., Helmchen, F., and Kampa, B.M. (2012). Distinct functional properties of primary and posteromedial visual area of mouse neocortex. J. Neurosci. *32*, 9716–9726.

Rossant, C., Kadir, S.N., Goodman, D.F.M., Schulman, J., Hunter, M.L.D., Saleem, A.B., Grosmark, A., Belluscio, M., Denfield, G.H., Ecker, A.S., Tolias, A.S., Solomon, S., Buzsáki, G., Carandini, M., and Harris, K.D. (2016). Spike sorting for large, dense electrode arrays. Nature Neuroscience *19* (4), 634–641. https://doi.org/10.1038/nn.4268.

Roth, M.M., Dahmen, J.C., Muir, D.R., Imhof, F., Martini, F.J., and Hofer, S.B. (2016). Thalamic nuclei convey diverse contextual information to layer 1 of visual cortex. Nat. Neurosci. *19*, 299–307.

Rovó, Z., Ulbert, I., and Acsády, L. (2012). Drivers of the primate thalamus. J. Neurosci. *32*, 17894–17908.

Rueden, C.T., Schindelin, J., Hiner, M.C., DeZonia, B.E., Walter, A.E., Arena, E.T., and Eliceiri, K.W. (2017). ImageJ2: ImageJ for the next generation of scientific image data. BMC Bioinformatics *18*, 529.

Saalmann, Y.B., and Kastner, S. (2015). The cognitive thalamus. Front. Syst. Neurosci. *9*, 39.

Saalmann, Y.B., Pinsk, M.A., Wang, L., Li, X., and Kastner, S. (2012). The pulvinar regulates information transmission between cortical areas based on attention demands. Science *337*, 753–756.

Sabbagh, U., Govindaiah, G., Somaiya, R.D., Ha, R.V., Wei, J.C., Guido, W., and Fox, M.A. (2020). Diverse GABAergic neurons organize into subtype-specific sublaminae in the ventral lateral geniculate nucleus. J. Neurochem. Published online June 4, 2020. https://doi.org/10.1111/jnc.15101.

Sauerbrei, B.A., Guo, J.-Z., Cohen, J.D., Mischiati, M., Guo, W., Kabra, M., Verma, N., Mensh, B., Branson, K., and Hantman, A.W. (2020). Cortical pattern generation during dexterous movement is input-driven. Nature *577*, 386–391.

Schindelin, J., Arganda-Carreras, I., Frise, E., Kaynig, V., Longair, M., Pietzsch, T., Preibisch, S., Rueden, C., Saalfeld, S., Schmid, B., et al. (2012). Fiji: an open-source platform for biological-image analysis. Nat. Methods *9*, 676–682.

Schuett, S., Bonhoeffer, T., and Hübener, M. (2002). Mapping retinotopic structure in mouse visual cortex with optical imaging. J. Neurosci. *22*, 6549–6559.

Schwarz, L.A., Miyamichi, K., Gao, X.J., Beier, K.T., Weissbourd, B., DeLoach, K.E., Ren, J., Ibanes, S., Malenka, R.C., Kremer, E.J., and Luo, L. (2015). Viral-genetic tracing of the input-output organization of a central noradrenaline circuit. Nature *524*, 88–92.

Shamonin, D.P., Bron, E.E., Lelieveldt, B.P.F., Smits, M., Klein, S., and Staring, M. (2014). Fast Parallel Image Registration on CPU and GPU for Diagnostic Classification of Alzheimer's Disease. Front. Neuroinform. *7*, 50.

Sherman, S.M. (2016). Thalamus plays a central role in ongoing cortical functioning. Nat. Neurosci. *19*, 533–541.

Sherman, S.M., and Guillery, R.W. (2011). Distinct functions for direct and transthalamic corticocortical connections. J. Neurophysiol. *106*, 1068–1077.

Shipp, S. (2003). The functional logic of cortico-pulvinar connections. Philos. Trans. R. Soc. Lond. B Biol. Sci. *358*, 1605–1624.

Siegle, J.H., López, A.C., Patel, Y.A., Abramov, K., Ohayon, S., and Voigts, J. (2017). Open Ephys: an open-source, plugin-based platform for multichannel electrophysiology. J. Neural Eng. *14*, 045003.

Soares, J.G.M., Diogo, A.C.M., Fiorani, M., Souza, A.P.B., and Gattass, R. (2004). Effects of inactivation of the lateral pulvinar on response properties of second visual area cells in Cebus monkeys. Clin. Exp. Pharmacol. Physiol. *31*, 580–590.

Tervo, D.G.R., Hwang, B.-Y., Viswanathan, S., Gaj, T., Lavzin, M., Ritola, K.D., Lindo, S., Michael, S., Kuleshova, E., Ojala, D., et al. (2016). A Designer AAV Variant Permits Efficient Retrograde Access to Projection Neurons. Neuron *92*, 372–382.

Tohmi, M., Meguro, R., Tsukano, H., Hishida, R., and Shibuki, K. (2014). The extrageniculate visual pathway generates distinct response properties in the higher visual areas of mice. Curr. Biol. *24*, 587–597.

Trageser, J.C., and Keller, A. (2004). Reducing the uncertainty: gating of peripheral inputs by zona incerta. J. Neurosci. *24*, 8911–8915.

Tyson, A., Rousseau, C.V., Niedworok, C.J., and Margrie, T.W. (2020). SainsburyWellcomeCentre/cellfinder: Version 0.3.7. https://zenodo.org/record/3665329#.YIh-M7VKiUk.

Van Essen, D.C. (1979). Visual areas of the mammalian cerebral cortex. Annu. Rev. Neurosci. *2*, 227–263.

Vong, L., Ye, C., Yang, Z., Choi, B., Chua, S., Jr., and Lowell, B.B. (2011). Leptin action on GABAergic neurons prevents obesity and reduces inhibitory tone to POMC neurons. Neuron *71*, 142–154.

Wall, N.R., Wickersham, I.R., Cetin, A., De La Parra, M., and Callaway, E.M. (2010). Monosynaptic circuit tracing in vivo through Cre-dependent targeting and complementation of modified rabies virus. Proc. Natl. Acad. Sci. USA *107*, 21848–21853.

Walt, S., van der, Schönberger, J.L., Nunez-Iglesias, J., Boulogne, F., Warner, J.D., Yager, N., Gouillart, E., and Yu, T. (2014). scikit-image: image processing in Python. PeerJ. *2*, e453.

Wang, Q., and Burkhalter, A. (2007). Area map of mouse visual cortex. J. Comp. Neurol. *502*, 339–357.

Wang, Q., Ding, S.-L., Li, Y., Royall, J., Feng, D., Lesnar, P., Graddis, N., Naeemi, M., Facer, B., Ho, A., et al. (2020). The Allen Mouse Brain Common Coordinate Framework: A 3D Reference Atlas. Cell *181*, 936–953.e20.

Wickersham, I.R., Finke, S., Conzelmann, K.-K., and Callaway, E.M. (2007). Retrograde neuronal tracing with a deletion-mutant rabies virus. Nat. Methods *4*, 47–49.

Wurtz, R.H., McAlonan, K., Cavanaugh, J., and Berman, R.A. (2011). Thalamic pathways for active vision. Trends Cogn. Sci. *15*, 177–184.

Zhou, H., Schafer, R.J., and Desimone, R. (2016). Pulvinar-cortex interactions in vision and attention. Neuron *89*, 209–220.

Zhou, N.A., Maire, P.S., Masterson, S.P., and Bickford, M.E. (2017). The mouse pulvinar nucleus: organization of the tectorecipient zones. Vis. Neurosci. *34*, E011.

Zhuang, J., Ng, L., Williams, D., Valley, M., Li, Y., Garrett, M., and Waters, J. (2017). An extended retinotopic map of mouse cortex. eLife *6*, e18372.

CellPress
OPEN ACCESS

STAR★METHODS

KEY RESOURCES TABLE

REAGENT or RESOURCE	SOURCE	IDENTIFIER
Bacterial and virus strains		
AAV1-Flex-nGFP-2A-G (G)	A.J. Murray	Reardon et al., 2016
ssAAV-retro/2-hSyn1-chI-iCre-WPRE-SV40p(A)	ETH Zurich VVF – plasmid from addgene	Cat# 24593
AAV8-flex-GT (TVA)	Addgene	Cat# 26198
CVS-N2cΔG-mCherry	A.J. Murray	Reardon et al., 2016
rAAV1/Sync-Flex-ChrimsonR-tdT	UNC vector core	Klapoetke et al., 2014
AAV1.CAG.flex.tdTomato	Addgene	Cat# 28306
AAV1.hSyn.GCaMP6f.WPRE.SV40	Addgene	Cat# 100837
AAV1-syn-jGCaMP7b-WPRE	Addgene	Cat# 104489
ssAAV-1/2-hEF1a-mCherry-WPRE-bGHp(A)	ETH Zurich VVF – plasmid from addgene	V212-1
ssAAV-1/2-hEF1a-EGFP-WPRE-bGHp(A)	ETH Zurich VVF – plasmid from addgene	V211-1
ssAAV-1/2-hEF1a-EBFP2-WPRE-bGHp(A)	ETH Zurich VVF – plasmid from addgene	V213-1
Chemicals, peptides, and recombinant proteins		
DiI	Thermo-Fisher	Cat# D3911
DiO	Thermo-Fisher	Cat# V22886
CTB-488	Thermo-Fisher	Cat# C34775
CTB-594	Thermo-Fisher	Cat# C34777
Experimental models: Organisms/strains		
C57BL/6JRj	Janvier Labs or Charles River	N/A
B6;129P2-Pvalbtm1(cre)Arbr/J	The Jackson Laboratory	JAX: 008069
Slc17a6tm2(cre)Lowl/J	The Jackson Laboratory	JAX: 016963
Software and algorithms		
ScanImage	Vidrio Technologies, LLC	Pologruto et al., 2003
Allen Common Coordinate Framework	https://atlas.brain-map.org/	Wang et al., 2020
Kilosort	https://github.com/MouseLand/Kilosort	Pachitariu et al., 2016
Elastix	https://elastix.lumc.nl/	Shamonin et al., 2014
Phy	https://github.com/cortex-lab/phy	
BakingTray	https://github.com/SainsburyWellcomeCentre/BakingTray	Campbell, 2020
Cellfinder	https://github.com/brainglobe/cellfinder	Tyson et al., 2020
scikit-image	https://scikit-image.org/	Walt et al., 2014
matplotlib	https://matplotlib.org/3.1.0/index.html	Caswell et al., 2019
Fiji	https://imagej.net/Fiji	Schindelin et al., 2012
GPflow	https://github.com/GPflow/GPflow	Matthews et al., 2017
CircStat	http://www.mathworks.com/matlabcentral/fileexchange/10676-circular-statistics-toolbox-directional-statistics	Berens, 2009
TimeSeriesAnalysis	https://bitbucket.org/DylanMuir/timeseriesanalysis/	Muir et al., 2020

RESOURCE AVAILABILITY

Lead contact

Further information and requests for resources and reagents should be directed to and will be fulfilled by the lead contact, Sonja B. Hofer (s.hofer@ucl.ac.uk).

Materials availability

This study did not generate new unique reagents.

Data and code availability

The datasets supporting the current study have not been deposited in a public repository because of their large size but are available from the lead contact on request.

EXPERIMENTAL MODEL AND SUBJECT DETAILS

All experiments were conducted in accordance with institutional animal welfare guidelines and licensed by the UK Home Office and the Swiss cantonal veterinary office. Mice used in this study were of either gender and were at least 6 weeks old at the start of the experiments. Mice were of the following genotype: C57BL/6j (Charles River, 46 mice for rabies tracing and two-photon imaging experiments); vGlut2-ires-cre (Vong et al., 2011, 2 mice for rabies tracing control experiments); PV-Cre (Hippenmeyer et al., 2005, 30 mice for optogenetic manipulation experiments). For more details, refer to the key resources table.

METHOD DETAILS

Surgical procedures and virus injections

Prior to surgery, mice were injected with dexamethasone (2–3 mg/kg), atropine (0.05–0.1 mg/kg) and analgesics (carprofen; 5 mg/kg). General anesthesia was induced either with a mixture of fentanyl (0.05 mg/kg), midazolam (5 mg/kg) and medetomidine (0.5 mg/kg) or with isoflurane (1%–5%). All injections were made in the right hemisphere and were performed using glass pipettes and a pressure injection system (Picospritzer III, Parker). For experiments that necessitated injections into visual cortical areas AL or PM, a customized head holder was implanted using dental cement (Heraeus Sulzer or C&B), and the skull above the posterior cortex was carefully thinned and sealed with a thin layer of light-cured dental composite (Tetric EvoFlow, Ivoclar Vivadent). Intrinsic imaging maps of visual cortical areas (see Intrinsic signal imaging) were obtained several days later to identify AL and/or PM prior to injections.

For mono-synaptic rabies tracing from specific LP projection neurons, we injected a retrograde AAV-Cre (Tervo et al., 2016, ssAAV-retro/2-hSyn1-chI-iCre-WPRE-SV40p(A), 90 - 100 nl, 7.90 × 10^{12} vg/mL; Viral Vector Facility Zurich; Addgene plasmid # 24593 from Patrick Aebischer) into either AL or PM through a small craniotomy. To mark the injection site, the pipette was coated with DiO. One week later, AAV1-Flex-nGFP-2A-G (G, 30 nl, 1.9 × 10^{13} vg/mL; Addgene plasmid # 26198 from Edward Callaway; Reardon et al., 2016) and AAV8-flex-GT (TVA, 1 × 10^{14} vg/mL, gift from AJ. Murray, Wall et al., 2010) were stereotaxically injected into LP in the right hemisphere (−2.2 mm posterior to bregma, −1.6 mm lateral to bregma, −2.60 mm below the cortical surface to target AL-projecting LP neurons and −2.1 mm posterior to bregma, −1.5 mm lateral to bregma, −2.6 mm below the cortical surface to target PM-projecting LP neurons). Three days later, EnvA-pseudotyped G-deleted rabies virus (Reardon et al., 2016, CVS-N2c$^{\Delta G}$-mCherry, 60 nl, > 1 × 10^{8} vg/mL) was injected at the same LP coordinates. The craniotomies were sealed with Tetric Evoflow light-cured dental composite. Ten to twelve days after the last injection, mice were perfused for histology (see Histology). Rabies tracing control experiments (Figures S2A–S2D) followed the same protocol, but no retroAAV-Cre was injected. For control experiments to estimate the injection size of the retroAAV-Cre of the rabies experiments (Figures S1E and S1F), 90 or 100nl of ssAAV-1/2-hEF1a-mCherry-WPRE-bGHp(A) (5.7 × 10^{12} vg/mL; Viral Vector Facility Zurich), ssAAV-1/2-hEF1a-EGFP-WPRE-bGHp(A) (3.60 × 10^{12} vg/mL; Viral Vector Facility Zurich) and ssAAV-1/2-hEF1a-EBFP2-WPRE-bGHp(A) (5.40 × 10^{12} vg/mL; Viral Vector Facility Zurich) were injected at different locations of the right cortical hemisphere. After 8 days, mice were perfused for histology. For retrograde tracing data presented in Figure S3, fluorescent conjugate cholera toxin B (CTB; recombinant cholera toxin subunit B conjugated with Alexa fluorophores: 0.2% CTB-488 and CTB-594; Life Technologies) was injected into AL and PM.

For experiments involving two-photon calcium imaging, AAV1.hSyn.GCaMP6f.WPRE.SV40 (120 nl, 2 × 10^{13} vg/mL Penn Vector Core/Addgene; diluted 1:2 to 1:10 in saline) or AAV1.hSyn.GCaMP7b.WPRE (for experiments with V1 silencing, 2 × 10^{13} vg/mL Penn Vector Core/Addgene; diluted 1:5 in saline; Addgene viral prep # 100837-AAV1 and Addgene viral prep # 104489-AAV1 from Douglas Kim & GENIE Project, Chen et al., 2013; Dana et al., 2019) was injected either into V1, AL or PM guided by intrinsic imaging maps (see Intrinsic signal imaging) or into LP (60 nl) using stereotaxic coordinates ranging from −1.45 to −2.2 mm posterior to bregma, 1.4 to 1.6 mm lateral to bregma and 2.55 to 2.7 mm below the cortical surface.

For the experiments involving optogenetic manipulations (Figures 3, S4, and S6), AAV1.Syn.DIO.ChrimsonR.tdTomato (120 nl, 3.9 × 10^{12} vg/mL, 1:5 dilution in saline solution, UNC vector core, Addgene plasmid # 62723, from Edward Boyden) or AAV1.CAG.DIO.tdTomato (control, 120 nl, 2.6 × 10^{13} vg/mL, diluted 1:5 in saline, Addgene viral prep # 28306-AAV1, from Edward Boyden) was injected into AL, PM (single injection) or V1 (5 to 7 injections). A craniotomy of 4–5 mm diameter was made over the right hemisphere to include V1 and higher visual areas. The craniotomy was sealed with a glass coverslip and cyanoacrylate glue (UltraGel; Pattex). If not already in place from intrinsic signal imaging, a head holder was attached to the skull using dental cement (Heraeus Sulzer or C&B). Animals were given analgesics (buprenorphine 0.1 mg/kg) at the end of surgery and repeatedly during recovery. Some animals additionally received antibiotics after the surgery (enrofloxacin 5 mg/kg). Imaging started approximately 2 to 3 weeks after the virus injection.

Intrinsic signal imaging

To determine the location of cortical visual areas AL and PM, mice underwent optical imaging of intrinsic signals (Kalatsky and Stryker, 2003; Schuett et al., 2002). Two to three days after the implantation of a head holder and thinning of the skull (see Surgical procedures), mice were initially sedated (chlorprothixene, 0.7 mg/kg) and then lightly anesthetized with isoflurane (0.5%–1% in O_2) delivered via a nose cone. Visual cortex was illuminated with 700-nm light split from a LED source into two light guides. Imaging was performed with a tandem lens macroscope focused 500 μm below the cortical surface and a bandpass filter centered at 700 nm with 10 nm bandwidth (67905; Edmund Optics). Images were acquired with a rate of 6.25 Hz with a 12-bit CCD camera (1300QF; VDS Vosskühler), a frame grabber (PCI-1422; National Instruments) and custom software written in Labview (Texas Instruments). The visual stimulus was generated using the open-source Psychophysics Toolbox (Kleiner et al., 2007) based on MATLAB (MathWorks) and consisted of a 25° large square-wave grating, (0.08 degrees per cycle) drifting at 4 Hz, presented on a gray background alternatively at two positions, centered at 10° elevation and either 60° or 90° azimuth. Frames in the second following stimulus onset were averaged across 16 to 32 grating presentations to generate intrinsic maps.

Histology

Mice were euthanized with a dose of pentobarbital (80 mg/kg) and transcardially perfused with 4% paraformaldehyde. Brains were extracted and post-fixed overnight in 4% paraformaldehyde. For animals that had undergone two-photon imaging, brains were embedded in 4% agarose (A9539, Sigma), cut in 200 μm sagittal slices on a vibratome (HM650V; Microm) and imaged on a slide scanner (Zeiss AxioScan). For animals used for anatomical tracing experiments, brains were embedded in 5% agarose and imaged using a custom-built serial-section two-photon microscope (Mayerich et al., 2008; Ragan et al., 2012). Coronal slices were cut at a thickness of 50 μm using a vibratome (Leica VT1000), and optical sections were acquired every 8 μm for rabies experiments and every 25 μm for CTB experiments. Scanning and image acquisition were controlled by ScanImage v5.6 (Vidrio Technologies, USA) using a custom software wrapper for defining the imaging parameters (Campbell, 2020). For better identification of rabies virus starter cells expressing rabies, G and TVA (Figure S1G), a subset of slices were mounted in a hard-set mounting medium (2.5% DABCO (D27802; Sigma), 10% polyvinyl alcohol (P8136; Sigma), 5% glycerol, 25 mM Tris buffer pH 8.4) and imaged at higher resolution on a confocal microscope (Leica SP8).

Two-photon calcium imaging

In vivo imaging experiments were performed as previously described (Roth et al., 2016). Mice were housed with an inverted light-dark cycle starting at least 5 days before the first imaging experiments. All experiments were performed during the dark phase. Animals were handled and accustomed to head restraint for 3 - 5 days. Mice were free to run on a 20-cm-diameter Styrofoam cylinder. Their running speed was measured using either an optical mouse (Logitech G700) or a rotary encoder (Kubler Encoder 1000 ppr). Imaging was performed using a commercial resonance scanning two-photon microscope (B-Scope; Thorlabs) and a Mai Tai DeepSee laser (SpectraPhysics) at 960 nm with a 16 × water immersion objective (0.8 NA; Nikon). Images of 512 × 512 pixels with fields of view ranging from 180 × 180 μm to 100 × 100 μm were acquired at a frame rate of 15 or 30 Hz using ScanImage (Pologruto et al., 2003). Axonal bouton calcium measurements were performed in cortical layer 1 (62 ± 54 μm below the cortical surface). Somatic recordings were performed in layer 2/3 (166 ± 13 μm below the cortical surface). The laser power under the objective never exceeded 30 mW. The surface blood vessel pattern above imaging sites was compared with the blood vessel pattern from intrinsic signal imaging maps to confirm that imaged neurons or boutons were located within a particular cortical area.

Visual stimulation

During presentation of visual stimuli, the power supply of the monitor backlight was controlled using a custom-built circuit to present visual stimuli only at the resonant scanner turnaround points in between two subsequent imaging lines (when data were not acquired) (Leinweber et al., 2014).

Visual response characterization

Visual stimuli were generated using the open-source Psychophysics Toolbox (Kleiner et al., 2007) based on MATLAB (MathWorks) and were presented full-field on one monitor at approximately 20 cm from the left eye of the mouse, covering 110° degrees of visual space. Visual stimuli consisted of sinusoidal gratings of all combinations of 5 different spatial frequencies (0.02, 0.04, 0.08, 0.16 or 0.32 cycles per degree) and 5 different temporal frequencies (0.5, 1, 2, 4, 8 cycles per second), presented at 4 orientations, drifting in 8 directions (0 to 360 degrees in 45 degrees increment). To avoid onset responses that would compromise the measure of temporal frequency preferences, gratings remained static for 1.2 s, before drifting for 2.15 s before the next static grating appeared. This set of 200 stimuli was randomized and presented 6 to 8 times.

Visuo-motor response characterization

A virtual environment consisting of a linear corridor with varying wall patterns as described previously (Poort et al., 2015; Roth et al., 2016, gratings and black and white circles on a gray background), was created in a game engine (Unity) and presented on two monitors (U2312HM; Dell) in front of the animal. The instantaneous running speed of the animal was used by a custom software written in Labview (National Instruments) to control the speed at which the animal moved through the virtual environment. Mice were

habituated to this configuration for at least 2 - 3 days with 1 to 2 sessions per day. The length of the experimental session increased gradually from ~15 min to 1 hour. Mice were encouraged to run by giving them soy milk rewards through a spout either at random times or in particular corridor positions. For two-photon imaging, the optic flow was ‘uncoupled’ from the running speed, such that the animal’s locomotion did not control its movement through the virtual corridor. Instead, a movie of the virtual corridor with optic flow generated by the animal in a previous session was replayed to the mouse. Sessions in which mice showed signs that they had learned to anticipate the reward by slowing down before reward delivery, and sessions in which the median running speed was below 3 cm/s were excluded from analysis. These two criteria ensured that only recordings were included for further analysis in which animals were familiar with the virtual environment, showed a wide distribution of running speeds, and did not display stereotypical behavior.

Optogenetic manipulation

To silence neuronal activity in a cortical area, we used a 637-nm laser (Coherent) connected to a 200- (for AL, PM) and 200-400 μm (for V1) optical fiber (CFMLC22 or CFMLC14, Thorlabs). The fiber was placed above the cortex (AL, PM or V1), in between the objective used for two-photon imaging and the glass coverslip covering the craniotomy. To combine two-photon imaging and optogenetic stimulation, the laser for optogenetic stimulation was synchronized to the resonant scanner turnaround points (when data were not acquired) (Attinger et al., 2017). The laser power was set to an average of 10 mW during stimulation. Visual stimulation was performed similarly as described above, but oblique grating orientations were excluded. Each stimulus was presented with and without laser activation and the 200 stimuli (5 spatial frequencies * 5 temporal frequencies * 4 directions * 2 laser conditions) were randomly interleaved and presented 6 to 8 times. Gratings were static for 1.2 s, before drifting for 2.3 s. When present, the laser was active for 2 s starting 0.5 s after the beginning of the static grating and 0.7 s before the onset of the drifting grating. To prevent the optogenetic manipulation during one stimulus from affecting activity in the following trial, a gray screen was displayed between stimuli for 500 ms.

Electrophysiology

To estimate the effect of optogenetic manipulation on cortical activity, electrophysiological recordings were performed after two-photon calcium imaging in a subset of the PV-Cre mice injected with AAV-flex-Chrimson in AL or PM (Figures S4B and S4C). On the day of the recording, mice were anaesthetized under 1%–2% isoflurane, the glass coverslip covering the craniotomy was removed and the exposed cortical surface was covered with Kwik-Cast sealant (World Precision Instruments). Mice recovered from surgery for 1-2 h before the recording and were then head-fixed on a Styrofoam cylinder. The craniotomy was bathed in cortex buffer containing (in mM) 125 NaCl, 5 KCl, 10 Glucose monohydrate, 10 HEPES, 2 MgSO4 heptahydrate, 2 CaCl2 adjusted to pH 7.4 with NaOH. A silver wire was placed in the bath for referencing. One or two NeuroNexus silicon probes (A2x32-5mm-25-200-177-A64), labeled with DiI, were lowered to 600-1000 μm below the cortical surface using a micromanipulator (Sensapex). The craniotomy was then covered with 1.5% agarose in cortex buffer. Voltages from 64 or 128 channels were acquired through amplifier boards (RHD2132, Intan Technologies) at 30 kHz per channel, serially digitized and sent to an Open Ephys acquisition board via a SPI interface cable (Siegle et al., 2017). Photoactivation and visual stimulation were then performed as described above (Visual stimulation and Optogenetic manipulation).

QUANTIFICATION AND STATISTICAL ANALYSIS

CTB and mono-synaptic rabies tracing

Full resolution datasets (voxels of 2x2x8 μm for rabies experiments and 2x2x25 μm for CTB experiments) were rescaled to isometric voxels of 10 μm^3 and registered to the Allen Mouse Common Coordinate Framework version 3 (Lein et al., 2007; Wang et al., 2020), using Elastix (Klein et al., 2010; Shamonin et al., 2014). CTB positive cells were manually counted using the cell counter plugin of Fiji (Rueden et al., 2017; Schindelin et al., 2012). For analysis of rabies virus tracing experiments, only brains were included in which we could locate starter cells within LP borders, in which G positive cells were found exclusively in LP and in which the retroAAV injection (labeled with DiO and targeted using intrinsic signal imaging) was located in AL or PM as defined by the Allen Mouse Common Coordinate Framework (Lein et al., 2007).

Fluorescent, rabies-positive cells were automatically detected using cellfinder (Tyson et al., 2020) (commit 9ccc641a). Cell candidates were detected as threshold crossings on filtered images and classified as cell or non-cell by a deep neural network. The deep neural network was trained using a large dataset of manually identified cells and non-cells. Running the same automatic cell detection on control brains from Figures S2A–S2D yielded a low number of false-positive cells (19 and 53 cells, i.e., 1.1 ± 1.2% of the total number of cells detected in experimental brains), mostly corresponding to bright particles at the surface of the brain. The location of detected cells was analyzed using custom scripts in Python and figures were generated using matplotlib (Caswell et al., 2019). In the cortex, a fraction of cells was detected in the white matter just below layer 6b (see examples in Figures 1B, 1C, and S1H–S1J). To account for these, any cell detected in the white matter less than 50 μm from the cortical border assigned by the common coordinate framework was allocated to layer 6b of the closest cortical area. The total number of cells per brain varied from animal to animal (see Figure S2D), therefore, cell numbers per brain region are reported as proportion of detected cells per brain. Dorsal views of cortical layers (Figure 2C) are the maximum projection of each layer of interest along the dorso-ventral axis.

Dorsal projections of cell density histograms in different layers across cortical areas (Figures 2D and S2H) were computed in 3D bins of 20 μm x 20 μm (along the antero-posterior and medio-lateral axis) x the thickness of the layer. Values were normalized by the total number of cells per brain and then averaged across brains. To take into account the variable layer thickness at different cortical positions, particularly in the dorsal part of the brain where the layers are tangential to the dorso-ventral axis, each bin was divided by its volume. Rabies-virus positive cell density is therefore expressed as percentage of total cells per cubic millimeter. Transverse views of cell density histograms (Figures S3D–S3F) were computed in 3D bins of 10 μm x 10 μm x 200 μm and upsampled to 5 μm^2 pixels with linear interpolation using scikit resize (Walt et al., 2014).

Two-photon imaging

Image stacks were processed using custom-written scripts in MATLAB (Mathworks) as described in Roth et al. (2016). Briefly, to correct for x-y motion, two-photon imaging frames were registered to a 30-frame average using a phase-correlation algorithm. Frames with large motion were detected by inspecting the registration displacement results and were subsequently discarded from further analysis. Regions of interest (ROIs) were detected semi-automatically using intensity thresholding combined with PCA-ICA refinement and validated and refined manually. All time-series were extracted and analyzed with custom written functions using the TimeSeriesAnalysis package (Muir et al., 2020) (see key resources table). For recordings of neuronal somata, contaminating signals coming from densely labeled neuropil were subtracted using an Asymmetric Student-t model (ast_model available here: https://github.com/BaselLaserMouse/ast_model). ΔF/F calcium transients were obtained by using the 25th percentile over the entire fluorescence distribution as F0. Firing rates per imaging frame were then inferred from ΔF/F using a compressive sensing technique (Dyer et al., 2013; Roth et al., 2016).

Electrophysiology

Spikes were sorted with Kilosort (Pachitariu et al., 2016) and Phy (Rossant et al., 2016) using procedures previously described (Chabrol et al., 2019). Each unit was attributed to the channel on which the extracellular waveform had the highest amplitude. Recording depth was estimated based on the DiI track and only channels in close proximity (< 100 μm) to the Chrimson injection site were included for analysis. Single units with average firing rate significantly higher in laser trials than in controls trials (putative PV+ interneurons expressing chrimson) were excluded from the analysis.

Analysis of visual responses

The response to each stimulus was measured as the inferred firing rate averaged over a window starting 250 ms after the onset of grating movement and ending either at the end of the stimulus presentation (Figure 4) or at the end of the laser stimulation (Figures 3, S4, and S6). Responses were then fitted using a Gaussian process (GP) regression model as previously described (Kim et al., 2018). The GP fit has several advantages compared to more classical parametric methods: (1) it does not assume independence between the different stimulus dimensions (e.g., between spatial and temporal frequency tuning), (2) it does not constrain the shape of the response profile (for instance to be Gaussian) but only assumes that response variations are continuous; (3) it is probabilistic and therefore provides not only an estimate of the average response but also of its variance; (4) it easily allows for integration of other parameters that influence neuronal activity but are harder to include in parametric fits, such as the running speed of the animal. GP predictions were made from five predictors: the spatial frequency, the temporal frequency and the direction of the stimulus, the average running speed of the animal during stimulus presentation and the presence of the laser (0 for control trials and 1 for laser trials).

As previously described (Kim et al., 2018), the GP regression model is fitted by estimating the parameters of the kernel function (x_i, x_j), which defines the covariance of the neuronal activity as a function of the similarity of stimuli x_i and x_j, defined by their respective spatial frequencies SF, temporal frequencies TF, directions θ, running speed s and the presence of laser L. We used a product of a squared exponential (SE) kernel for spatial and temporal frequencies and a periodic kernel for direction:

$$\kappa(x_i, x_j) = \sigma_\kappa^2 \exp\left(-\left(\frac{(SF_i - SF_j)^2}{2l_{SF}^2} + \frac{(TF_i - TF_i)^2}{2l_{TF}^2} + \frac{(\theta_i - \theta_j)^2}{2l_\theta^2} + \frac{(s_i - s_j)^2}{2l_s^2} + \frac{(L_i - L_j)^2}{2l_L^2}\right)\right) + \sigma_\varepsilon^2 \delta_{ij}$$

Length scale parameters l_{SF}, l_{TF}, l_θ, ls, and l_L determine how quickly (x_i, x_j) declines with changes of that stimulus dimension. Variance parameters σ_κ^2 and σ_ε^2 correspond to the stimulus-dependent and stimulus-independent (i.e., noise) components of the response. δ_{ij} is Kronecker delta and is one if i = j and zero otherwise. Optimization is accomplished by maximizing the likelihood p(r|X) of observed responses r given the set of stimuli X.

The GP model was implemented in Python using the gpflow library (Matthews et al., 2017). We used Gamma(2,1) as a prior for length scale parameters l_{SF}, l_{TF}, l_θ, l_s, and l_L. In addition, to avoid overfitting, we constrained l_{SF}, l_{TF}, $l_s \geq 0.25$. l_L was unconstrained. After optimizing the kernel parameters, we searched for the stimulus that evoked the maximum response using the Nelder-Mead method of the scipy minimize function. We then defined a signal to noise ratio (SNR) as:

$SNR = \frac{\widehat{r_{best}}}{\sqrt{\widehat{var_{best}}}}$ where r_{best} and var_{best} are the predicted mean and variance of the response to the best stimulus. ROIs were considered responsive if the signal to noise ratio was above two and the R^2 of the fit, defined as $R^2 = 1 - \Sigma_i((r_i-p_i)^2) / \Sigma_i((r_i - r)^2)$ where r_i is the

response at stimulus *i* and p_i is the prediction of the GP fit for the same stimulus, was above 0.1. All results presented in the paper could be qualitatively reproduced using a parametric fit instead of the GP fit.

Determining a preferred stimulus feature of a neuron (e.g., preferred spatial frequency) is only meaningful if its response is significantly modulated across this stimulus dimension. When reporting preferred visual stimuli (Figures 4, S5, and S6), we therefore only included ROIs for which the predicted response to the preferred stimulus, r_{best}, was at least 1.33 standard deviations above that to the stimulus evoking the smallest response along that dimension r_{worst} (e.g., for responses to different spatial frequencies for stimuli with the same temporal frequency and grating direction,):

$\widehat{r}_{best} - 0.66\sqrt{\widehat{var}}_{best} > \widehat{r}_{worst} + 0.66\sqrt{\widehat{var}}_{worst}$ To estimate the preferred stimulus, the search of the maximum of the GP fit was bounded to the range of presented spatial and temporal frequencies ([0.02 - 0.32] and [0.5 - 8]). For Figure S6 the same search was performed with the laser parameter L fixed to 0 for estimating the preferred stimulus in control trials and fixed to 1 for laser trials. Preferred speed was defined as the ratio between the preferred temporal and spatial frequency. To average across boutons/neurons or display them on the same color scale, responses were z-scored using the mean and standard deviation of the inferred spike rate. Response matrices (Figures 4 and S6) were obtained by averaging the z-scored response amplitude of all visually responsive ROIs for every combination of spatial and temporal frequencies at the preferred direction of each ROI. The similarity between such matrices was then evaluated by computing the Pearson correlation coefficient between the average matrix of each individual imaging session and the average matrix of either all AL neurons (Figures 4I and 4J), or of all PM neurons (Figures 4K and 4L).

To measure response specificity to different visual stimulus features (Figures S5I–S5K), we computed tuning curves of predicted responses to varying spatial or temporal frequencies while keeping all other stimulus parameters fixed to those evoking the peak response. We then measured the full-width half maximum of the resulting curve (examples of such curves for individual neurons can be found in Figure 4D).

To quantify the effect of optogenetic manipulation on LP boutons, we included all visual stimuli that evoked a response with SNR of the GP prediction above 2, and with an amplitude of at least $^2/_3$ of the response to the best stimulus. Responses of individual trials to these stimuli were pooled to test the effect of laser stimulation using a Wilcoxon rank-sum test (trials with and without laser are not paired). Boutons were defined as significantly suppressed if their average response was significantly lower in laser trials than in control trials (alpha < 0.05). To compare tuning curves in response to different visual stimulus properties with and without optogenetic laser stimulation, we included boutons that were significantly responsive in both conditions, defined as SNR of the response to the preferred stimulus above 2. Tuning curves of individual boutons were plotted centered on and relative to the preferred stimulus (Figures S6D and S6J) and included responses at the preferred grating direction and the preferred temporal or spatial frequency for spatial and temporal frequency tuning curves, respectively.

Analysis of visuo-motor responses

To identify responsive boutons or neurons, we measured the skewness of $\Delta F/F$ values of individual ROIs over the recording. ROIs with skewness > 1 were considered to be responsive. For each responsive bouton or neuron, a normalized cross-correlation was computed by obtaining time-dependent Pearson correlation coefficients between its inferred spike rate and a behavioral variable (running speed or optic flow speed resampled at the imaging frame rate) over a range of lags between -1 to 1 s (corresponding to 60 different lags with 30 Hz imaging frame rate). For each behavioral variable and each bouton or neuron, we then determined the lag with the highest absolute correlation coefficient. From these values, we computed the median lag of the neuronal population as m_{Lag} (separately for running speed and optic flow speed; Figure S7). We computed a mean cross-correlation coefficient for each bouton or neuron and each behavioral variable (R_{RS} for running speed and R_{OF} for optic flow speed) by averaging the time-dependent Pearson coefficients over lags in a window of 250 ms centered on the population m_{Lag}. To determine if a bouton or a soma was correlated with a behavioral variable, we defined a circular threshold: the magnitude of the vector |R| composed by [R_{OF}, R_{RS}] was computed as the square root of the sum of squared R_{OF} and squared R_{RS}. Only boutons with $|R| >= 0.1$ were included in the following analysis. The interaction angle θ was determined using the mean cross-correlation coefficients, and was computed as $\theta = \text{atan}(R_{RS}/R_{OF})$. For estimating the circular median interaction angle per session, we computed $\theta_{pop} = \text{atan}(R_{RSpop}/R_{OFpop})$ where R_{RSpop} and R_{OFpop} are the median R_{RS} and R_{OF} across boutons or somata. For each bouton or soma, we calculated a selectivity index as the difference between absolute R_{OF} and absolute R_{RS} divided by the sum between absolute R_{OF} and absolute R_{RS}.

Statistics

We used two-sided Wilcoxon rank-sum tests for independent group comparisons, and two-sided Wilcoxon signed-rank tests for paired tests. We used circular statistics and circular metrics (Berens, 2009) when required (Figures 5J and S8G; Kruskal Wallis for circular data). Raw p values are reported throughout the manuscript, significance thresholds in all figures have been adjusted for multiple comparisons using Bonferonni correction. Tests were performed using either MATLAB or rpy2. No statistical methods were used to pre-determine experimental sample sizes.

Neuron, Volume *109*

Supplemental information

Visual intracortical and transthalamic pathways carry distinct information to cortical areas

Antonin Blot, Morgane M. Roth, Ioana Gasler, Mitra Javadzadeh, Fabia Imhof, and Sonja B. Hofer

Visual intracortical and transthalamic pathways carry distinct information to cortical areas

Supplementary information

Acronyms

ACRONYM	FULL NAME	ACRONYM	FULL NAME
**a.u.**	Arbitrary units	**PM**	Posteromedial visual area
**AAV**	Adeno-associated virus	**POL**	Posterior limitans nucleus of the thalamus
**ACC**	Anterior cingulate cortex	**POR**	Postrhinal area
**AL**	Anterolateral visual area	**PPT**	Posterior pretectal area
**AM**	Anteromedial area	**PV**	Parvalbumin
**APN**	Anterior pretectal nucleus	**RHP**	Retrohippocampal region
**Apo**	Posterior auditory area	**RL**	Rostrolateral visual area
**AUD**	Auditory areas	**RN**	Red nucleus
**CTB**	CholeratoxinB	**RS**	Running speed
**CTX**	Cortex	**RSP**	Retrosplenial cortex
**DAPI**	4′,6-diamidino-2-phenylindole	**RSPagl**	Agranular retrosplenial cortex
**dLGN**	Dorsal lateral geniculate nucleus	**RSPd**	Dorsal part of the retrosplenial cortex
**ECT**	Ectorhinal areas	**RSPv**	Ventral part of the retrosplenial cortex.
**FWHM**	Full width half maximum	**RT**	Reticular thalamic nucleus.
**G**	Rabies virus glycoprotein	**SCdg**	Superior colliculus, deep gray layer
**GP**	Gaussian Process	**SCdw**	Superior colliculus, deep white layer
**HVA**	Higher visual areas	**SCig**	Superior colliculus, intermediate gray layer
**IGL**	Intrageniculate leaflet	**SCiw**	Superior colliculus, intermediate white layer
**INC**	Interstitial nucleus of Cajal	**SCop**	Superior colliculus, optic layer
**LD**	lateral dorsal nucleus of the thalamus	**SCsg**	Superior colliculus, superficial gray layer
**LI**	Laterointermediate area	**SCzo**	Superior colliculus, zonal layer
**LM**	Lateromedial area	**SF**	Spatial frequency
**LP**	Lateral posterior nucleus of the thalamus	**SS**	Somatosensory areas
**M1**	Primary motor cortex	**SubG**	Subgeniculate nucleus
**M2**	Secondary motor cortex	**TEa**	Temporal association area
**MPT**	Medial pretectal area	**TF**	Temporal frequency
**MRN**	Midbrain reticular nucleus	**TVA**	Tumor virus A protein
**ND**	Nucleus of Darkschewitsch	**V1**	Primary visual cortex
**NOT**	Nucleus of the optic tract	**VGluT2**	Vesicular glutamate transporter 2
**NPC**	Nucleus of the posterior commissure	**vLGN**	Ventral lateral geniculate nucleus
**OF**	Optic Flow	**WT**	Wild type
**OP**	Olivary pretectal nucleus	**ZI**	Zona incerta
**PL**	Prelimibic area		

Figure S1

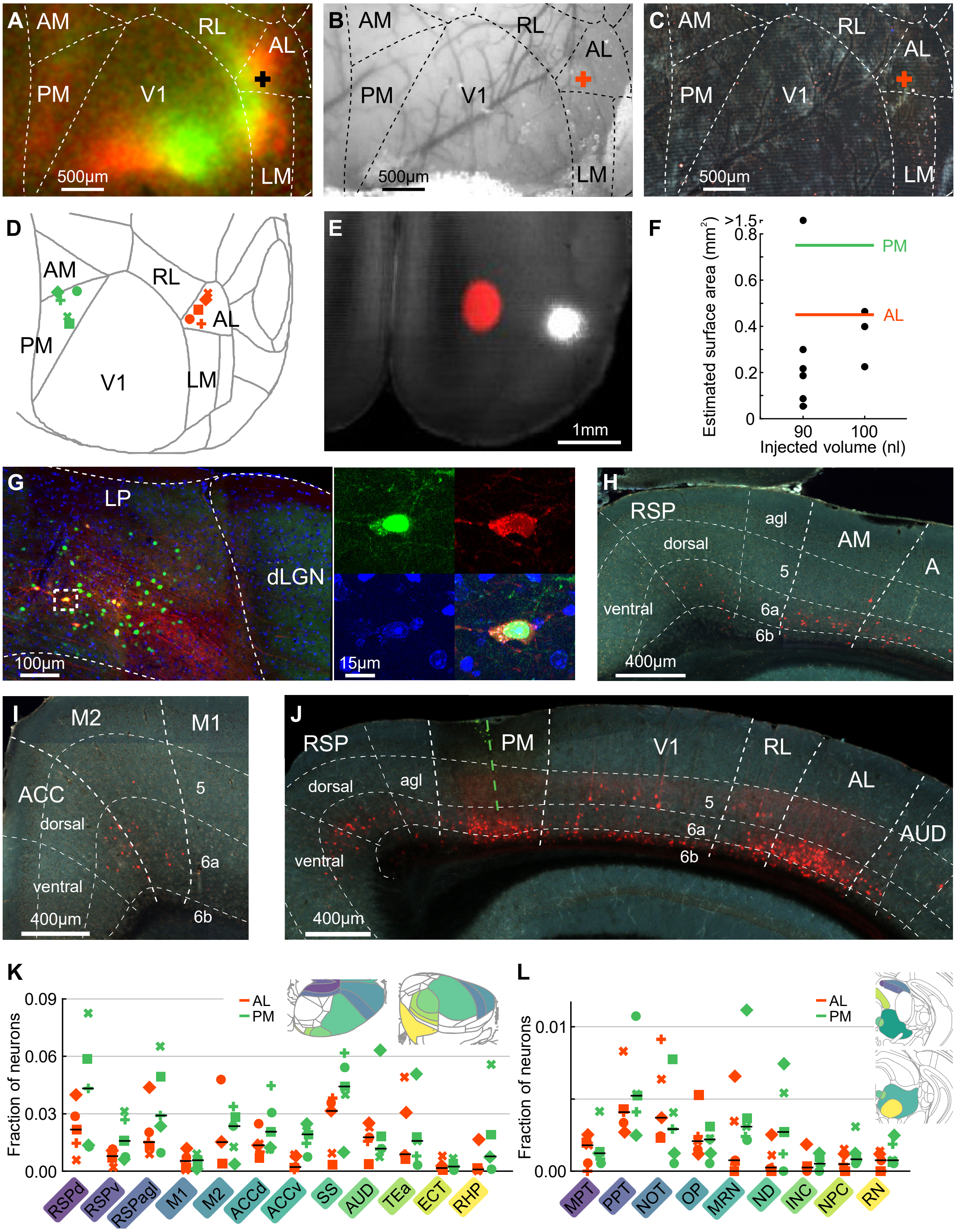
A
AM
RL
AL
PM
V1
LM
500μm
B
C
D
E
1mm
F
>1.5
PM
AL
Estimated surface area (mm²)
Injected volume (nl)
90
100
G
LP
dLGN
100μm
15μm
H
RSP
agl
dorsal
AM
A
ventral
5
6a
6b
400μm
I
M2
M1
ACC
dorsal
ventral
J
PM
V1
RL
AL
AUD
K
L
Fraction of neurons
RSPd
RSPv
RSPagl
M1
M2
ACCd
ACCv
SS
AUD
TEa
ECT
RHP
MPT
PPT
NOT
OP
MRN
ND
INC
NPC
RN

Figure S1. Mono-synaptic rabies tracing: targeting of visual areas and additional data. Related to Figure 1.

(A) Example intrinsic signal imaging map used to identify and target higher visual area AL for injection of retroAAV-Cre. Intrinsic responses evoked by two spatially separated visual stimuli (see STAR Methods) are color-coded in green and red. The cross marks the injection site. AL: anterolateral area, AM: anteromedial area, LM: lateromedial area, PM: posteromedial area, RL: rostro lateral area, V1: primary visual cortex.

(B) Surface blood vessel pattern corresponding to the intrinsic imaging map in (A).

(C) Dorsal view of the same area shown in (A,B) in the perfused brain reconstructed after serial-section two-photon imaging. The brain was registered to the Allen common coordinate framework and area borders were aligned to the intrinsic map in (A) using the blood vessel pattern in (B).

(D) Dorsal view of cortical visual areas with retroAAV-Cre injection locations in AL and PM.

(E) Dorsal view of an example brain showing the extent of fluorescent protein expression after injections of two different AAV constructs (red: AAV-mCherry, injection of 100 nl; white: AAV-eBFP, injection of 90 nl). These experiments were performed to estimate the size of injections of retroAAV-Cre which could not be directly measured since the retroAAV construct was not coupled to a fluorescent protein.

(F) Surface area estimations of fluorescent protein expression after AAV injections of volumes similar to retroAAV-Cre injections. Surface areas are the full length at half maximum on the slice containing the core of the injection. Surface area sizes of AL and PM based on the Allen common coordinate framework are indicated by horizontal lines.

(G) Left: confocal image of a coronal slice through the lateral posterior thalamic nucleus (LP) injection site, showing starter neurons expressing G, TVA and rabies virus (green and red overlap), and neurons expressing G and TVA only (green). Dashed lines indicate the borders of LP and the dorsal lateral geniculate nucleus (dLGN). Right: magnified image of a rabies starter cell in LP. Nuclear green label indicates the presence of G protein, cytosolic green label reflects TVA expression and red label indicates the presence of rabies virus. DAPI staining is shown in blue.

(H,I) Example images of coronal slices showing rabies-labelled cells (red) presynaptic of AL-projecting LP neurons after retroAAV injection into AL. A: anterior visual area, ACC: anterior cingulate cortex, agl: agranular, AM: anteromedial area, M1: primary motor cortex, M2: secondary motor cortex, RSP: retrosplenial cortex.

(J) Example image of a coronal slice showing the cortical injection site of retroAAV-Cre in PM (pipette track marked in green) and rabies-expressing cells (red) presynaptic to PM-projecting LP neurons. AL: anterolateral area, agl: agranular, AUD: auditory areas, PM: postromedial area, RL: rostrolateral area, RSP: retrosplenial cortex, V1: primary visual cortex.

(K) Fraction of rabies-positive cells presynaptic to AL- (orange) and PM-projecting (green) LP neurons across cortical areas not detailed in Figure 1. ACCd: anterior cingulate cortex, dorsal part, ACCv: anterior cingulate cortex, ventral part, AUD: auditory areas, ECT: ectorhinal areas, M1: primary motor cortex, M2: secondary motor cortex, RHP: retrohippocampal region, RSPagl: agranular part of the retrosplenial cortex, RSPd: dorsal part of the retrosplenial cortex, RSPv: ventral part of the retrosplenial cortex, SS: somatosensory areas, TEa: temporal association areas.

(L) Fraction of rabies-positive presynaptic cells across midbrain areas, excluding the anterior pretectal nucleus and the superior colliculus (presented in Figure 1). INC: interstitial nucleus of Cajal, MPT: medial pretectal area, MRN: midbrain reticular nucleus, ND: nucleus of Darkschewitsch, NOT: nucleus of the optic tract, NPC: nucleus of the posterior commissure, OP: olivary pretectal nucleus, PPT: posterior pretectal area, RN; red nucleus.

Figure S2

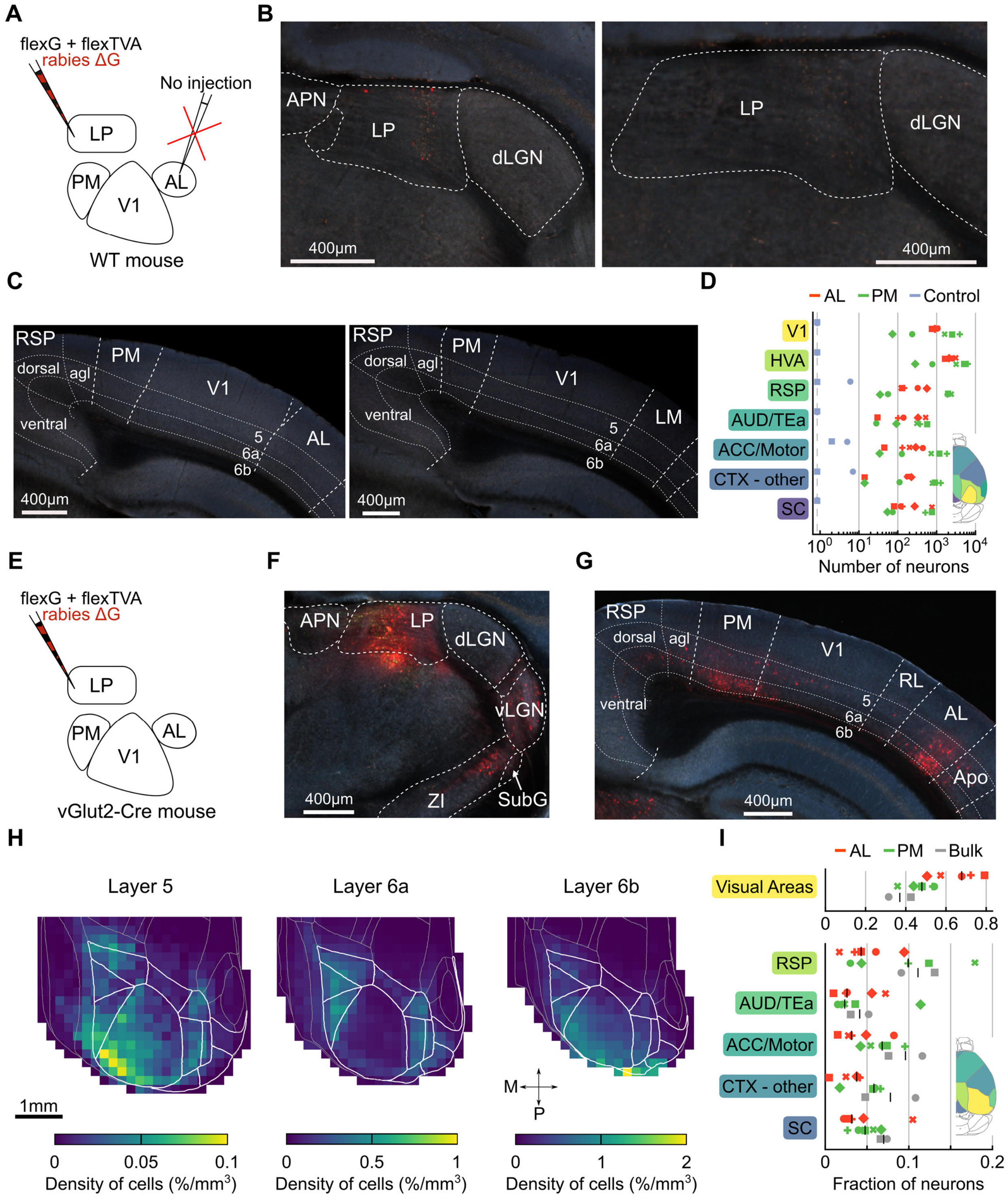

A
flexG + flexTVA
rabies ΔG
No injection
LP
PM
V1
AL
WT mouse
B
APN
LP
dLGN
400μm
C
RSP
dorsal
agl
PM
V1
ventral
5
6a
6b
AL
LM
400μm
D
AL
PM
Control
V1
HVA
RSP
AUD/TEa
ACC/Motor
CTX - other
SC
Number of neurons
E
flexG + flexTVA
rabies ΔG
vGlut2-Cre mouse
F
vLGN
ZI
SubG
G
RL
Apo
H
Layer 5
Layer 6a
Layer 6b
1mm
M
P
Density of cells (%/mm3)
I
Bulk
Visual Areas
Fraction of neurons

Figure S2. Mono-synaptic rabies tracing: control experiments and rabies tracing from LP neurons independent of projection target. Related to Figure 2.

(A) Design of rabies control experiment. The experimental protocol was similar to the one described in Figure 1, but no retroAAV-cre was injected. WT: wild-type

(B) Coronal sections through LP of two different animals showing no G or TVA expression.

(C) Coronal sections through the cortex from the same animals presented in (B) showing the absence of rabies-positive presynaptic cells.

(D) Numbers of rabies-positive cells detected by the automated cell counting software across brain areas in brains injected with retroAAV in AL (orange) in PM (green), and without injection of retroAAV-Cre (blue). Markers represent single animals. Dashed line indicates 0.

(E) Schematic of the experimental design. To label cells presynaptic to LP neurons without specific projection target, we injected AAV-flex-G, AAV-flex-TVA and rabies virus into LP of VGluT2-Cre mice.

(F) Coronal slice through the LP injection site showing rabies-positive cells in subcortical areas, presynaptic to LP neurons without specific projection target.

(G) Coronal slice showing rabies-positive cells presynaptic to LP neurons in cortical areas.

(H) Dorsal view of the average relative density of cells presynaptic to LP neurons per volume (see STAR Methods) in layer 5 (left), layer 6a (middle) and layer 6b (right); 2 mice. White lines indicate the border of cortical areas as in Figure 2A.

(I) Fraction of rabies-positive cells presynaptic to AL-projecting LP neurons (orange), PM-projecting LP neurons (green), and the general LP population (grey). Markers represent single animals. Bottom right corner: dorsal view of color-coded cortical areas.

ACC/Motor: anterior cingulate areas and motor areas, agl: agranular, AL: anterolateral area, APN: anterior pretectal nucleus, Apo: posterior auditory area, AUD/TEa: auditory areas and temporal association areas, CTX: cortex, dLGN: dorsal lateral geniculate nucleus, HVA: higher visual areas, LM: lateromedial area, LP: lateral posterior nucleus, PM: posteromedial area, RL: rostrolateral area, RSP: retrosplenial cortex, SC: superior colliculus, SubG: subgeniculate nucleus, V1: primary visual area, vLGN: ventral lateral geniculate nucleus, WT: wild type. ZI: zona incerta

Figure S3

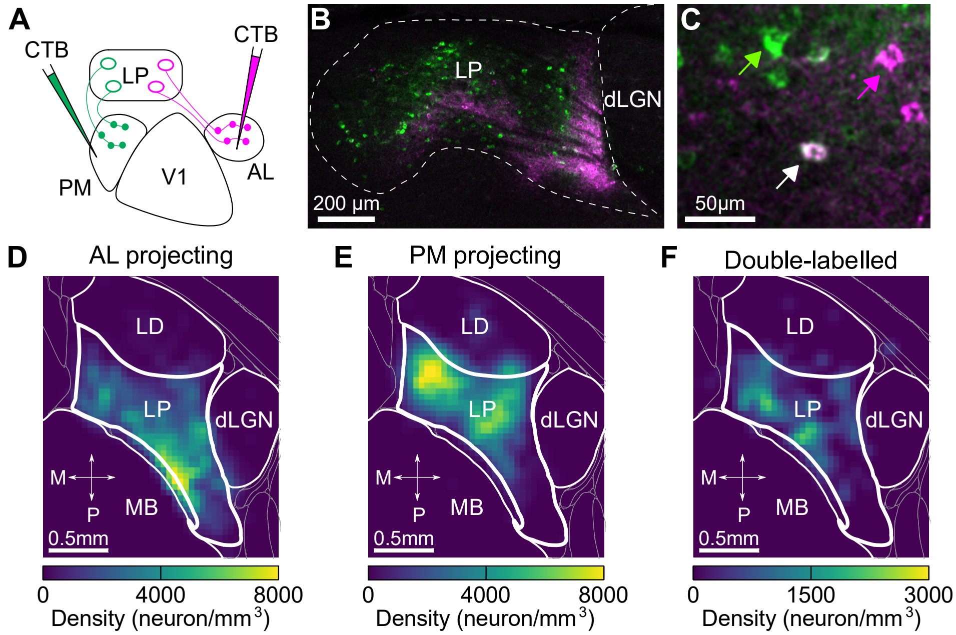

Figure S3. LP neurons projecting to AL and PM form two largely distinct populations. Related to Figures 1 and 2.

(A) Schematic of the experimental design. To determine the extent of overlap between AL and PM-projecting LP neurons, retrograde tracers of different colors (cholera toxin subunit B, CTB) were injected into AL and PM.

(B) Coronal section through LP showing the spatial distribution of PM-projecting (green) and AL-projecting (pink) neurons.

(C) Magnified area from (B). Arrows indicate examples of a PM-projecting LP neuron (green), an AL-projecting neuron (pink), and a neuron that targets both PM and AL (double-labelled, white).

(D-F) Dorsal view of the average relative density of AL-projecting (**D**, 3504 and 2102 neurons), PM-projecting (**E**, 2123 and 2406 neurons), and double-labelled neurons in LP (**F**, 442 and 414 neurons). Data from two mice. AL: anterolateral area, CTB: choleratoxin subunit B, dLGN: dorsal lateral geniculate nucleus, LD: lateral dorsal nucleus, LP: lateral posterior nucleus, M: medial, P: posterior, V1: primary visual cortex.

Figure S4

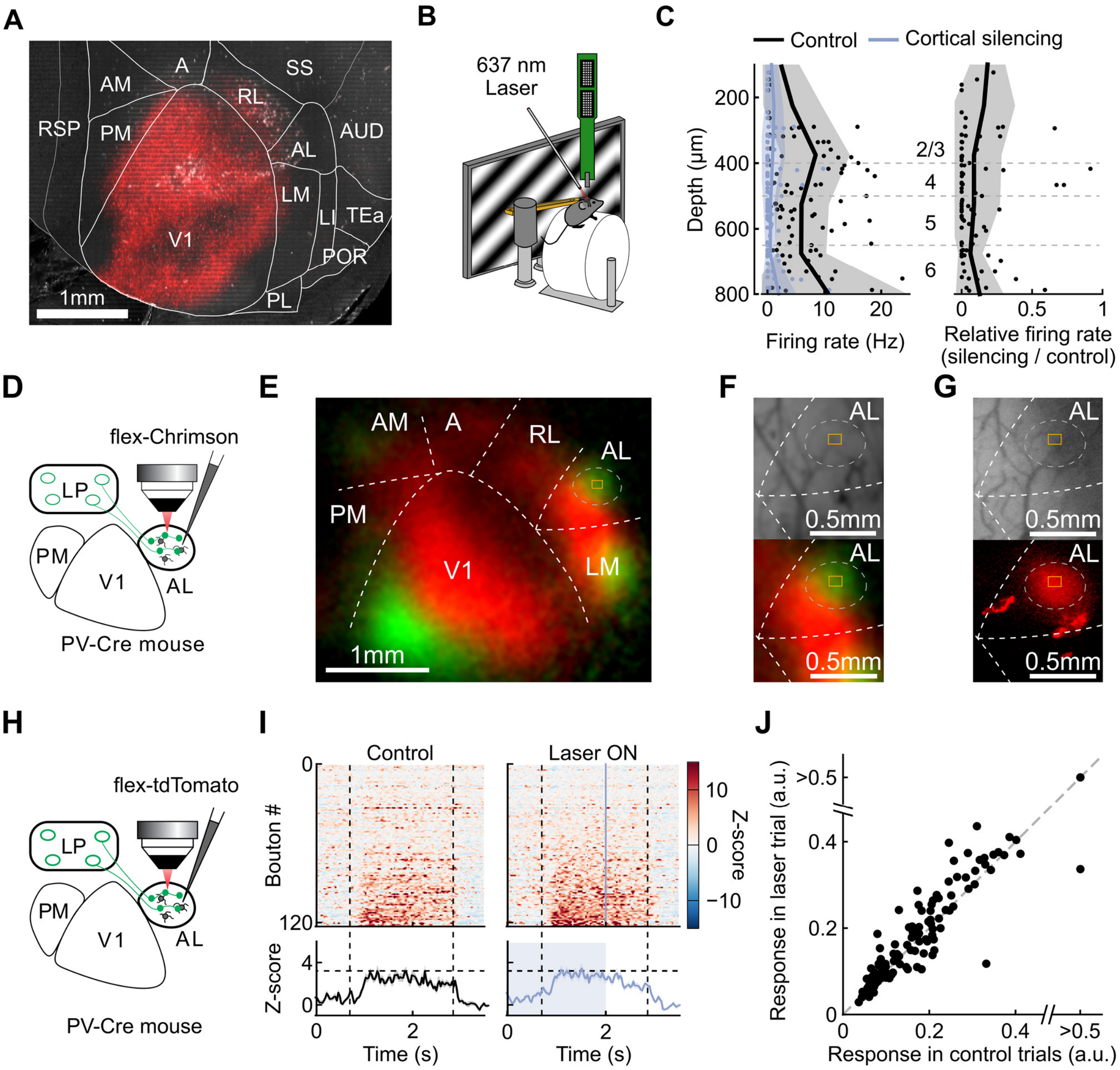

Figure S4. Optogenetic activation of PV neurons silences all cortical layers while laser light without Chrimson expression has no effect on LP responses. Related to Figure 3.

(A) Dorsal view of the cortex reconstructed after serial-section two-photon imaging showing the extent of Chrimson expression (red) in V1. The brain was registered to the Allen common coordinate framework (limits of cortical areas are indicated by white lines).

(B) Schematic of the experimental design for electrophysiological recordings. The cranial window was removed and a multi-channel silicon probe was inserted into a cortical location with Chrimson expression in AL, PM or V1. Awake mice were presented with gratings and a 637-nm laser was used to activate Chrimson-expressing PV neurons to silence cortical activity similar to experiments in Figure 3.

(C) Left: mean firing rate during visual stimulation with (blue) and without (black) laser; Right: proportion of remaining response in laser trials as a function of recording depth for single units not excited by the laser recorded from a silicon probe in a cortical location with Chrimson expression

during optogenetic activation of PV neurons. Dots depict individual units. Shading indicates standard deviation. 42 single units, 10 mice.

(D) Schematic of the experiment: flex-Chrimson was injected in AL of PV-Cre mice. GCaMP6f was expressed in LP and the activity of LP boutons was recorded in AL.

(E) Example intrinsic imaging map obtained to determine the location of cortical area AL for two-photon imaging and injection of AAV-flex-Chrimson showing responses to two spatially separated visual stimuli (red and green). Yellow square indicates the imaging site.

(F) Bottom: zoomed-in cutout from image in (E). Top: corresponding surface blood vessel pattern.

(G) Top: surface blood vessel pattern used to find the imaging site corresponding to (F). Bottom: image showing the extent of Chrimson expression (red) in AL, approximated by the dotted circle. Red specks outside the circle are imaging artifacts. Yellow square indicates the imaging site.

(H) Schematic of the laser control experiment. PV-Cre mice were injected with AAV-flex-tdTomato in AL and GCaMP6f in LP. Visually-evoked activity of LP boutons in AL was imaged with and without laser stimulation over AL.

(I) Top: time course of z-scored neuronal activity of individual boutons. For each bouton, activity was averaged across grating stimuli evoking a response (see STAR Methods) in control trials (left) and laser trials (right). Responses are aligned to the onset of the laser. Blue shading indicates time of laser stimulation. Dashed lines show duration of moving grating. Bottom: Average response across all boutons. Grey shading indicates sem. 123 boutons from 4 sessions in 4 mice

(J) Relationship between the average response of individual boutons with and without laser stimulation.

Figure S5

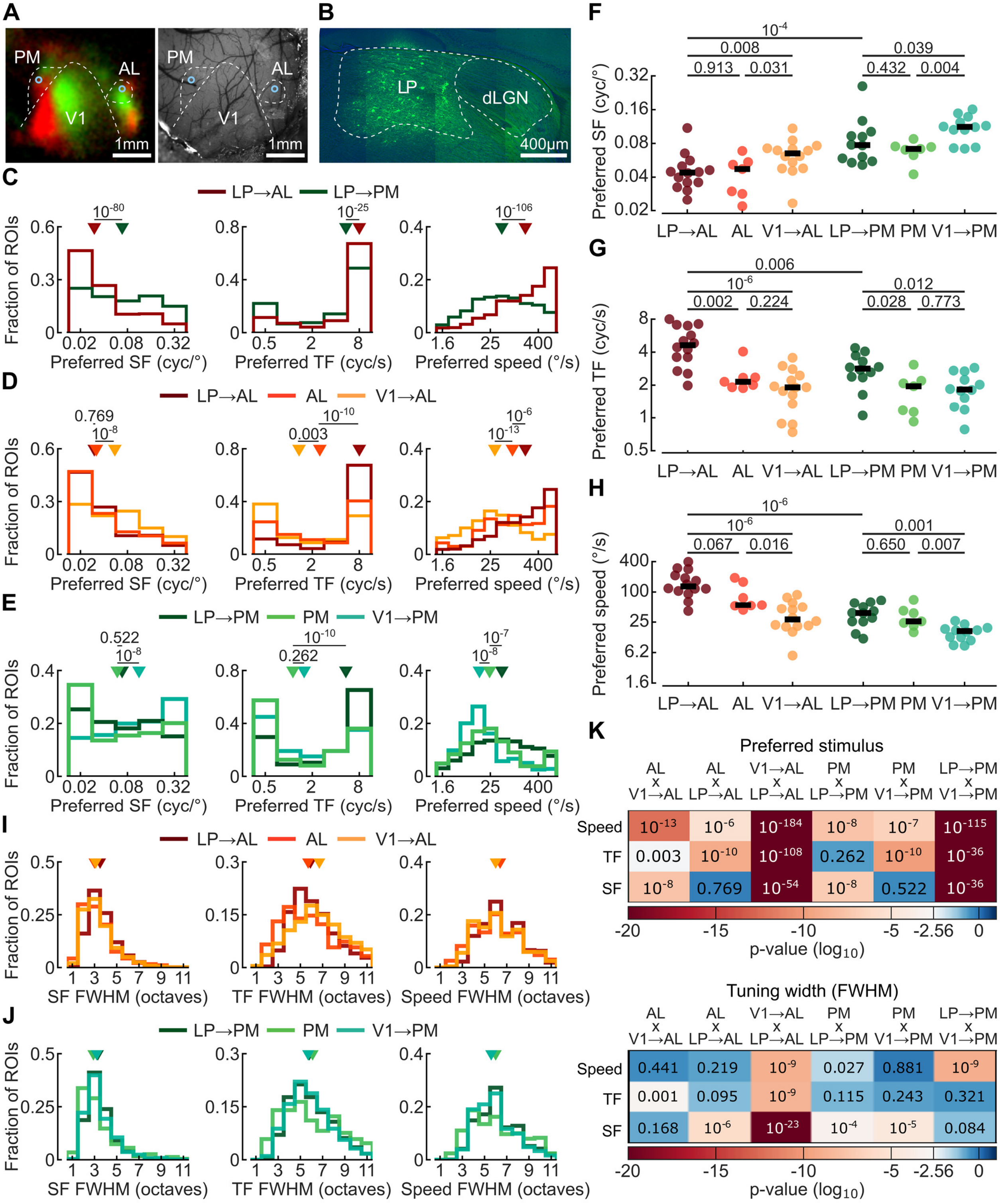

A
PM
AL
V1
1mm
B
LP
dLGN
400μm
C
LP→AL
LP→PM
D
LP→AL
AL
V1→AL
E
LP→PM
PM
V1→PM
Fraction of ROIs
Preferred SF (cyc/°)
Preferred TF (cyc/s)
Preferred speed (°/s)
F
Preferred SF (cyc/°)
G
Preferred TF (cyc/s)
H
Preferred speed (°/s)
LP→AL AL V1→AL LP→PM PM V1→PM
I
LP→AL
AL
V1→AL
J
LP→PM
PM
V1→PM
SF FWHM (octaves)
TF FWHM (octaves)
Speed FWHM (octaves)
K
Preferred stimulus
AL x V1→AL
AL x LP→AL
V1→AL x LP→AL
PM x LP→PM
PM x V1→PM
LP→PM x V1→PM
Speed
TF
SF
p-value (log10)
Tuning width (FWHM)

Figure S5. Further characterization of visual responses of LP inputs, V1 inputs and local neurons recorded in areas AL and PM. Related to Figure 4.

(A) Example intrinsic imaging map (left) showing responses to two spatially separated visual stimuli in green and red, and image of blood vessel pattern of the imaging area under the cranial window (right). Blue circles indicate the two imaging sites recorded in the posteromedial area (PM) and the anterolateral area (AL).

(B) Example image of GCamP6f injection site. dLGN: dorsal lateral geniculate nucleus, LP: lateral posterior nucleus.

(C) Distribution of preferred spatial frequency (left, 2237 LP boutons in AL and 2059 LP boutons in PM), preferred temporal frequency (middle, 1333 and 1128 LP boutons for AL and PM) and preferred speed (right, 2468 and 2342 boutons for AL and PM respectively) of grating stimuli for significantly modulated LP boutons recorded in AL (dark red) and in PM (green). Triangles indicate the median.

(D) Same as (C) for AL neurons (orange, 172, 126, and 231 neurons for SF, TF and speed respectively), LP boutons (dark red) and V1 boutons (yellow, 2555, 1637, and 2928 boutons for SF, TF and speed respectively) recorded in AL.

(E) Same as (C) for PM neurons (green, 214, 181, and 288 neurons for SF, TF and speed respectively), LP boutons (dark green) and V1 boutons (blue, 2327, 1659, and 2535 boutons for SF, TF and speed respectively) recorded in PM.

(F) Median preferred spatial frequencies of grating stimuli per imaging session of LP boutons in AL and PM, AL and PM neurons, and V1 boutons in AL and PM. Circles represent individual sessions. Black dashes indicate the median of all sessions.

(G) Same as (F) for preferred temporal frequencies of grating stimuli.

(H) Same as (F) for preferred grating speed.

(I) Distribution of full width half maxima (FWHM) of GP fit prediction (see STAR Methods) of spatial frequency (SF) tuning curves at the preferred temporal frequency and preferred direction of the grating (left), of temporal frequency (TF) tuning curves at the preferred spatial frequency and preferred direction (middle), and of speed tuning curves at the preferred grating direction (right).

(J) Same as (I) for full width half maxima of tuning curves of LP boutons in PM, PM neurons and V1 boutons in PM.

(K) P-values of Wilcoxon rank-sum test for relevant pairwise comparisons of stimulus preference (top) and tuning width (bottom). Numbers show raw p-values, color code indicates significance level, adjusted for multiple comparison using Bonferroni correction: white is the threshold, shades of red indicate significant p-values, shades of blue non-significant p-values. In all panels data from 7 sessions in 5 mice for AL neurons, 8 sessions in 5 mice for PM neurons, 14 sessions in 14 mice for LP boutons in AL, 12 sessions in 10 mice for LP boutons in PM, 14 sessions in 7 mice for V1 boutons in AL and 12 sessions in 7 mice for V1 boutons in PM.

Figure S6

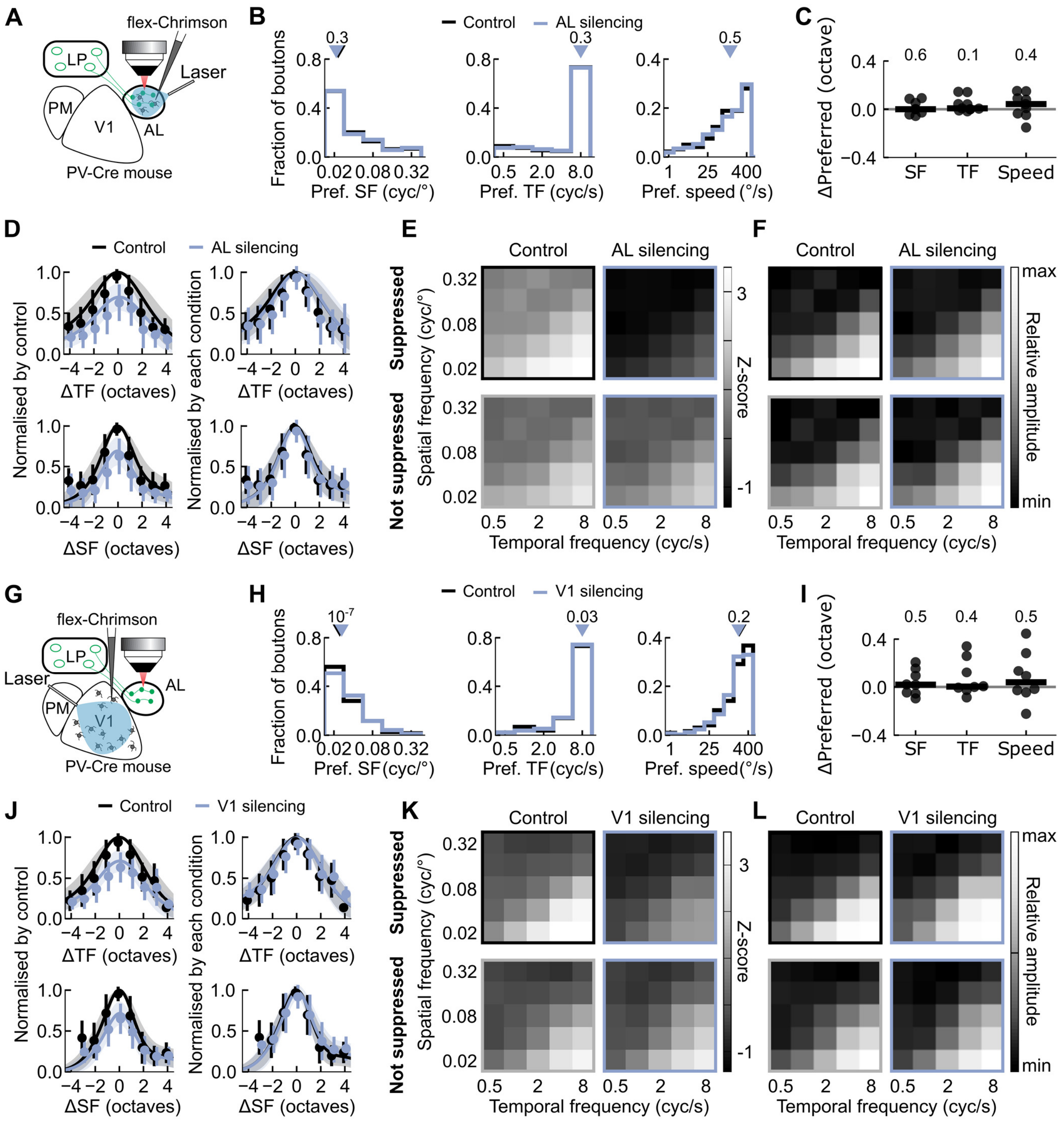

Figure S6. Cortical silencing of AL or V1 has minor effects on the response preferences and selectivity of LP inputs to AL. Related to Figures 3 and 4.

(A) Schematic of the experimental design, as in Figure 3F. Two-photon imaging of LP boutons was performed in AL while PV cells expressing Chrimson in cortical area AL were optogenetically activated to suppress local cortical activity.

(B) Distribution of preferred spatial frequency (left, 631 boutons), preferred temporal frequency (middle, 348 boutons) and preferred speed (right, 758 boutons) for boutons suppressed by AL silencing in control trials and AL silencing trials. Triangles indicate medians. P-values are indicated above. In panels (B-F) data from 9 sessions in 6 mice.

(C) Changes of stimulus preferences (laser trials – control trials) averaged by session. Black dots indicate single sessions, horizontal bar shows the median, p-values are indicated above.

(D) Left: Mean response curves to varying temporal (top) and spatial (bottom) grating frequencies of boutons significantly, but not fully suppressed during AL silencing (see STAR Methods, 181 and 269 boutons for TF and SF) in control trials (black) and laser trials (blue), normalized by the response to the preferred stimulus in control trials, plotted centered on and relative to the preferred frequency. Right: same as on the left, but normalized by the maximum response within each condition. Dots and error bars indicate means and the standard deviation of the raw data across boutons, while the lines and shading indicate means and standard deviation obtained from the prediction of the GP fit (see STAR Methods).

(E) Average spatial and temporal frequency population response matrices as in Figure 4 for LP boutons that were suppressed (top, 1009 boutons) or not suppressed (bottom, 1278 boutons) by AL silencing, in control trials (left) and silencing trials (right). Pearson correlation coefficient between the average response matrix of suppressed boutons in control trials and response matrices of individual sessions: 0.9 +/- 0.2 and 0.8 +/- 0.1 for suppressed and not suppressed boutons respectively, p = 0.3.

(F) Average population response matrices with values normalized to the maximum response amplitude during control trials (left) and AL silencing trials (right) for boutons that were suppressed (top) or not suppressed (bottom) by AL silencing.

(G) Schematic of the experimental design, as in Figure 3A. Two-photon imaging of LP boutons was performed in AL while PV cells expressing Chrimson in cortical area V1 were optogenetically activated to suppress local cortical activity.

(H) Distribution of preferred spatial frequency (left, 386 boutons), preferred temporal frequency (middle, 265 boutons) and preferred speed (right, 421 boutons) for boutons suppressed by V1 silencing in control trials and V1 silencing trials. Triangles indicate medians. In panels H-L data from 8 sessions in 3 mice.

(I) Same as (C) for experiments with V1 silencing.

(J) Same as (D) for experiments with V1 silencing.136 and 176 boutons for TF and SF respectively

(K) Average spatial and temporal frequency population response matrices of LP boutons in AL that were suppressed (top, 477 boutons) or not suppressed (bottom, 455 boutons) by V1 silencing, in control trials (left) and silencing trials (right). Pearson correlation coefficient between the average response matrix of suppressed boutons in control trials and response matrices of individual sessions: 0.9 +/- 0.2 and 0.9 +/- 0.1 for suppressed and not suppressed boutons respectively, p = 0.9.

(L) Average population response matrices for LP boutons in AL with values normalized to the maximum response amplitude during control trials (left) and V1 silencing trials (right) for boutons that were suppressed (top) or not suppressed (bottom) by V1 silencing.

Figure S7

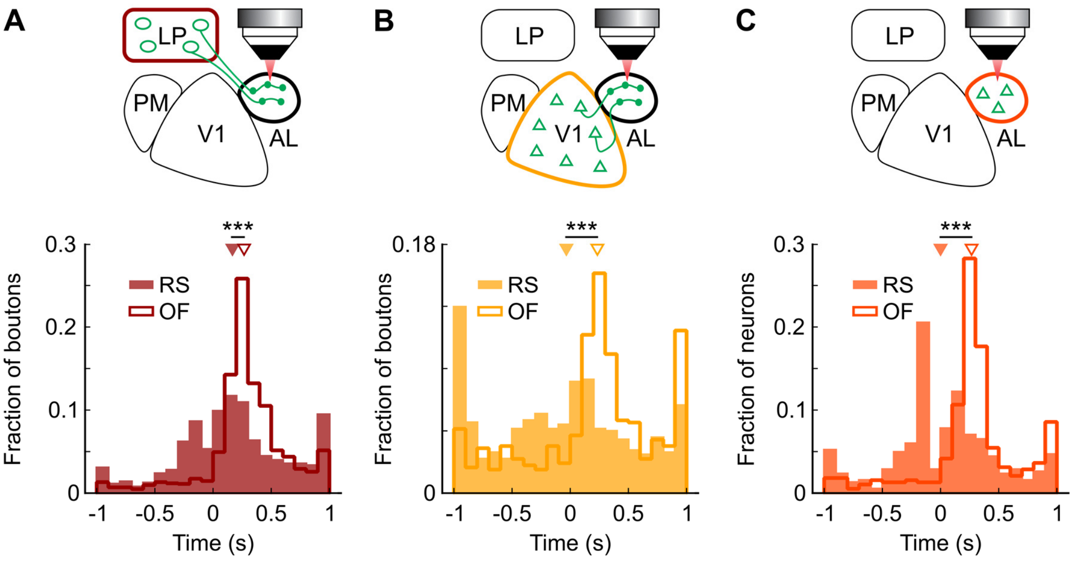

Figure S7. Distribution of lag times with highest absolute correlation coefficients between activity and running speed or optic flow speed. Related to STAR Methods.

(A-C) Top: schematic of the experimental design. Bottom: distribution of lag times between running speed (RS, filled histogram) or optic flow (OF, line) and the activity of LP boutons imaged in AL (A, 1437 boutons with correlation coefficients above the circular threshold of 0.1 from 43 sessions in 18 mice), V1 boutons in AL (B, 647 boutons with a correlation above the circular threshold of 0.1 from 6 sessions in 3 mice) and AL neurons (C, 385 neurons with correlation coefficients above the circular threshold of 0.1 from 15 sessions in 5 mice) for which the absolute cross-correlation was maximal for each neuron.***: $p < 10^{-15}$.

Figure S8

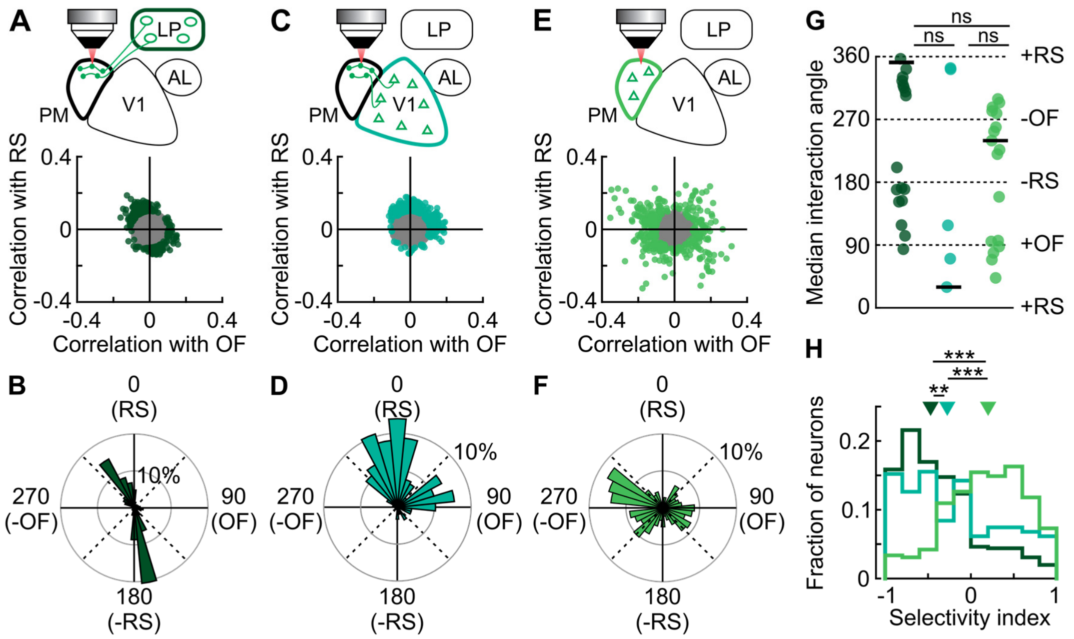

Figure S8. Thalamic and cortical inputs convey distinct visuo-motor information to area PM. Related to Figure 5.

(A) Top: schematic of the recording configuration. GCaMP6f was expressed in LP while calcium activity of LP boutons in cortical area PM was recorded using two-photon imaging. Bottom: relationship between the mean cross-correlation coefficients (see STAR Methods) of neuronal activity with running speed and optic flow speed for all responsive LP boutons (5678 boutons from 19 sessions in 8 mice). Only boutons with mean cross-correlation greater than 0.1 (colored points in scatter plot) were included in the analysis shown in (B), (G) and (H).

(B) Histogram in polar coordinates showing the distribution of interaction angles between the mean cross-correlation of activity with running speed (RS) and optic flow speed (OF) for LP boutons imaged in PM (425 boutons from 19 sessions in 8 mice).

(C) Same as (A) for V1 boutons imaged in PM (1556 boutons from 5 sessions in 3 mice).

(D) Same as (B) for V1 boutons imaged in PM (309 boutons from 5 sessions in 3 mice).

(E) Same as (A) for cortical neurons imaged in PM (976 neurons from 17 sessions in 5 mice).

(F) Same as (B) for cortical neurons imaged in PM (356 neurons from 17 sessions in 5 mice)

(G) Median interaction angles across single sessions for LP boutons in PM (left), V1 boutons in PM (middle) and PM neurons (right). Black horizontal lines represent the circular median across sessions. ns: non-significant, LP vs V1 $p = 0.011$, LP vs PM $p = 0.73$, V1 vs PM $p = 0.19$

(H) Distribution of selectivity indices (difference of the absolute mean cross-correlation of neuronal activity with optic flow and running speed divided by their sum) for individual LP boutons in PM (dark green), PM neurons (green) and V1 boutons in PM (blue). -1 indicates a high correlation only with running speed, 1 a high correlation only with optic flow and 0 indicates equally high correlation with running speed and optic flow of individual boutons/neurons. **: $p < 10^{-4}$; ***: $p < 10^{-16}$.
